# Prediction of resistance to bevacizumab plus FOLFOX in metastatic colorectal cancer—Results of the prospective multicenter PERMAD trial

**DOI:** 10.1371/journal.pone.0304324

**Published:** 2024-06-14

**Authors:** Thomas Seufferlein, Ludwig Lausser, Alexander Stein, Dirk Arnold, Gerald Prager, Stefan Kasper-Virchow, Michael Niedermeier, Lothar Müller, Stefan Kubicka, Alexander König, Petra Büchner-Steudel, Kai Wille, Andreas W. Berger, Angelika M. R. Kestler, Johann M. Kraus, Silke D. Werle, Lukas Perkhofer, Thomas J. Ettrich, Hans A. Kestler

**Affiliations:** 1 Department of Internal Medicine I, Ulm University Hospital, Ulm, Germany; 2 Institute of Medical Systems Biology, Ulm University, Ulm, Germany; 3 Faculty of Computer Science, Technische Hochschule Ingolstadt, Ingolstadt, Germany; 4 Hematology-Oncology Practice Eppendorf, University Cancer Center Hamburg, Hamburg, Germany; 5 Asklepios Cancer Center Hamburg, AK Altona, Hamburg, Germany; 6 Comprehensive Cancer Center Vienna, Medical University of Vienna, Vienna, Austria; 7 Medical Oncology, University Hospital Essen West German Cancer Center, Essen, Germany; 8 Private Practice, Memmingen, Germany; 9 Private Practice, Leer, Germany; 10 Cancer Center Reutlingen, Reutlingen Hospital, Reutlingen, Germany; 11 Department of Gastroenterology, Gastrointestinal Oncology and Endocrinology, University Medical Center Goettingen, Göttingen, Germany; 12 Internal Medicine I, Martin-Luther-University Halle-Wittenberg, Halle, Germany; 13 Hematology, Oncology, University Hospital Ruhr-University-Bochum, Minden, Germany; PLOS: Public Library of Science, UNITED KINGDOM

## Abstract

**Background:**

Anti-vascular endothelial growth factor (VEGF) monoclonal antibodies (mAbs) are widely used for tumor treatment, including metastatic colorectal cancer (mCRC). So far, there are no biomarkers that reliably predict resistance to anti-VEGF mAbs like bevacizumab. A biomarker-guided strategy for early and accurate assessment of resistance could avoid the use of non-effective treatment and improve patient outcomes. We hypothesized that repeated analysis of multiple cytokines and angiogenic growth factors (CAFs) before and during treatment using machine learning could provide an accurate and earlier, i.e., 100 days before conventional radiologic staging, prediction of resistance to first-line mCRC treatment with FOLFOX plus bevacizumab.

**Patients and methods:**

15 German and Austrian centers prospectively recruited 50 mCRC patients receiving FOLFOX plus bevacizumab as first-line treatment. Plasma samples were collected every two weeks until radiologic progression (RECIST 1.1) as determined by CT scans performed every 2 months. 102 pre-selected CAFs were centrally analyzed using a cytokine multiplex assay (Luminex, Myriad RBM).

**Results:**

Using random forests, we developed a predictive machine learning model that discriminated between the situations of “no progress within 100 days before radiological progress” and “progress within 100 days before radiological progress”. We could further identify a combination of ten out of the 102 CAF markers, which fulfilled this task with 78.2% accuracy, 71.8% sensitivity, and 82.5% specificity.

**Conclusions:**

We identified a CAF marker combination that indicates treatment resistance to FOLFOX plus bevacizumab in patients with mCRC within 100 days prior to radiologic progress.

## Introduction

Angiogenesis is a hallmark of cancer. Vascular endothelial growth factor (VEGF) is a major driver of tumor angiogenesis and monoclonal antibodies (mAbs) against VEGF are widely used in tumor treatment including metastatic colorectal cancer (mCRC) [1]. The addition of the anti-VEGF mAb bevacizumab to first-line combination chemotherapy has been shown to improve progression free survival (PFS) and overall survival (OS) of mCRC patients [2, 3]. Bevacizumab in combination with cytotoxic chemotherapy doublets such as 5-fluorouracil/folinic acid (5-FU/FA) plus oxaliplatin is recommended as first-line treatment for mCRC by numerous national and international guidelines [4–6]. Furthermore, there is evidence that Bevacizumab can be used beyond radiographic disease progression in certain circumstances [7]. Some patients with mCRC initially benefit from anti-VEGF mAbs but develop resistance to these drugs, even though they do not target the tumor but the non-transformed endothelial cells of the host [8]. To date, there are no biomarkers to predict resistance to anti-VEGF mAbs in patients. However, several resistance mechanisms have been identified in preclinical research [8]. One of them is the adaptive response of the tumor upon prolonged inhibition of VEGF in the presence of persistent hypoxia resulting in the upregulation of other proangiogenic cytokines and angiogenic growth factors (CAF) in the tumor including basic fibroblast growth factor (bFGF), platelet-derived growth factor (PDGF) and matrix metallopeptidase 9 (MMP-9) [9], a process termed evasive resistance [10]. Furthermore, distinct myeloid cell populations including macrophages, immature monocytic and granulocytic myeloid derived suppressor cells are recruited to the tumor by specific cytokines and contribute to the resistance to VEGF inhibition via the production of alternative CAF such as angiopoietin-2 (Ang2), granulocyte colony-stimulating factor (GCSF) and placental growth factor (PlGF) [9, 11]. A retrospective analysis of the VELOUR trial that examined the addition of the antiangiogenic agent aflibercept to fluorouracil, leucovorin, and irinotecan in advanced mCRC patients suggested that high VEGF-A and placental growth factor (PLGF) levels are associated with a resistance to bevacizumab [12]. These different CAFs can dynamically change their absolute values over time and potentially respond to both the antiangiogenic treatment itself as well as tumor progression. However, there is a remarkable intra- and interpersonal variation in the absolute values of specific CAFs [9]. This makes it difficult to assess absolute CAF values and their changes over time, even if the analysis is based on samples collected at many different times during treatment. In addition, there is no consistent pattern of CAFs over the course of treatment. The latter varies between different cytokines and situations such as treatment with anti-angiogenic agents, tumor response, and imminent or confirmed tumor progression [9]. This extremely high complexity hinders the straightforward establishment of CAF signatures that allow accurate prediction of resistance to anti-VEGF treatment, in particular if such a signature is to meet the requirement of predicting tumor progression significantly earlier than conventional staging by radiological imaging. Such prediction may be useful given the availability of other anti-angiogenic agents that target a broader spectrum of pro-angiogenic factors.

Machine learning and pattern recognition play an increasing role in biomarker discovery in particular due to the exponential complexity of the underlying selection task. E.g. plasma TIE-2 derived from the tumor vasculature has been described as a potential tumor vascular response biomarker for VEGF inhibitors in mCRC using bioinformatics [13]. Marker selection strategies and supervised classification experiments are required to ensure both the univariate quality of the selected markers and their synergistic effects on the prediction of the diagnostic classes. We hypothesized that a combination of modern CAF profiling by analyzing prospectively many biomarkers collected at defined time intervals may enable a more accurate prediction of anti-VEGF resistance during first-line treatment of mCRC patients with mFOLFOX6 plus bevacizumab. These considerations gave the rationale for the PERMAD trial aiming at establishing a CAF marker combination (CAFmC) to predict treatment resistance approximately three months prior to conventional radiologic staging.

## Materials and methods

### Study design

**Fig 1:**
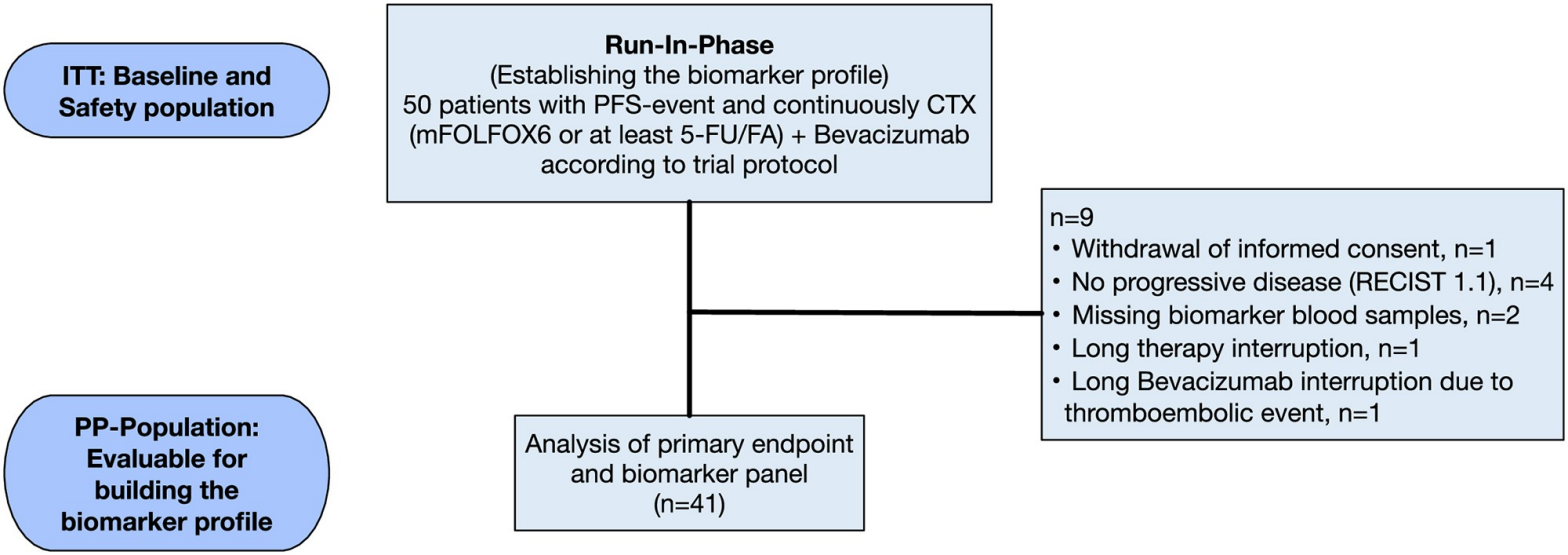
CONSORT participant flow diagram of the PERMAD trial. Abbreviations: components of mFOLFOX6: Fluorouracil, folinic acid, oxaliplatin.

PERMAD is a phase II, prospective, non-randomized, single arm, multicenter biomarker trial. Here, we report the results of the first exploratory run-in-phase that serves to identify a marker combination for predicting resistance to chemotherapy plus bevacizumab treatment. Between March 2015 and March 2020, 50 patients with treatment naive mCRC were recruited in 15 centers in Germany and Austria. All enrolled patients signed a written informed consent. For evaluation, patients had to receive continuously mFOLFOX6 plus bevacizumab or at least 5-FU plus bevacizumab as first-line systemic therapy until disease progression. As stipulated in the study protocol, patients with discontinuation of bevacizumab and/or 5-FU treatment (due to drug holidays, adverse events, or local ablative treatments) were not considered for the analysis, disregarding the reasons for discontinuation since their CAF values were likely to be affected by the treatment discontinuation. 41 out of these 50 patients were available for CAF analysis (Fig 1). Blood samples were prospectively collected prior to start of treatment (screening and baseline to allow identification of treatment unrelated individual variability) and subsequently every 14 days immediately before the administration of the next treatment cycle. Blood samples were examined centrally by Luminex^®^ technology (Myriad RBM, Austin, Texas, USA). 102 different cytokines with predicted or established involvement in angiogenesis were analyzed (S1 Table) in two cohorts differing only in the period of their recruitment (S1 and S2 Data). The first cohort comprised 26 patients with 48 blood samples obtained before and 287 samples during treatment (range of sampling time points per patient 4–27). The second cohort comprised 15 patients with 20 samples obtained before start of treatment and 297 during treatment (range of sampling time points per patient 4–46). The total number of sampling time points in both cohorts was 652 (68 before and 584 during treatment). Sampling was done until radiologic progress as determined by CT scans every two months. Due to the short time elapsed between progression CT and sample collection, the samples with the Myriad ID 10 8 and 21 7 were excluded from analysis (see Supplementary Information). 41 patients with CAF analyzed were evaluable for progression free survival (PFS), the primary clinical endpoint of the analysis, and 31 of these patients for overall survival (OS) analysis. The remaining patients were lost to follow-up in the recruiting centers.

The final selection of biomarkers was made according to the following criteria: A set of CAF that enables to predict disease progress within three months/100 days prior to radiologic progress as determined by CT using RECIST 1.1.

#### Sample size estimation

As a basis for the run-in-phase, the required sample size for the adjustment of classifier accuracy was estimated using Wald confidence intervals. Assuming an observed classification performance of 0.85 and a 95% confidence interval of 0.75–0.95, this results in a sample size of n = 49 (S1 Fig). A number of 49 patients who fulfil the inclusion criteria and give their consent for participation was considered to be a realistic size based on the consortium’s known treatment figures.

#### Ethics statement

The trial was conducted in compliance with the Declaration of Helsinki and the protocol approved by the respective ethics committees of the participating centers. All patients provided written informed consent prior to trial entry. The trial is registered with ClinicalTrials.gov (NCT02331927) and was funded by an unrestricted grant from SANOFI-Genzyme to Ulm University Hospital.

### Hierarchical clustering

An explorative unsupervised data analysis with hierarchical clustering (Ward method) was performed in order to identify a time span for a group of early and late disease progression. We analyzed all samples during treatment, normalized to treatment initialization [14, 15].

### Classification

**Fig 2:**
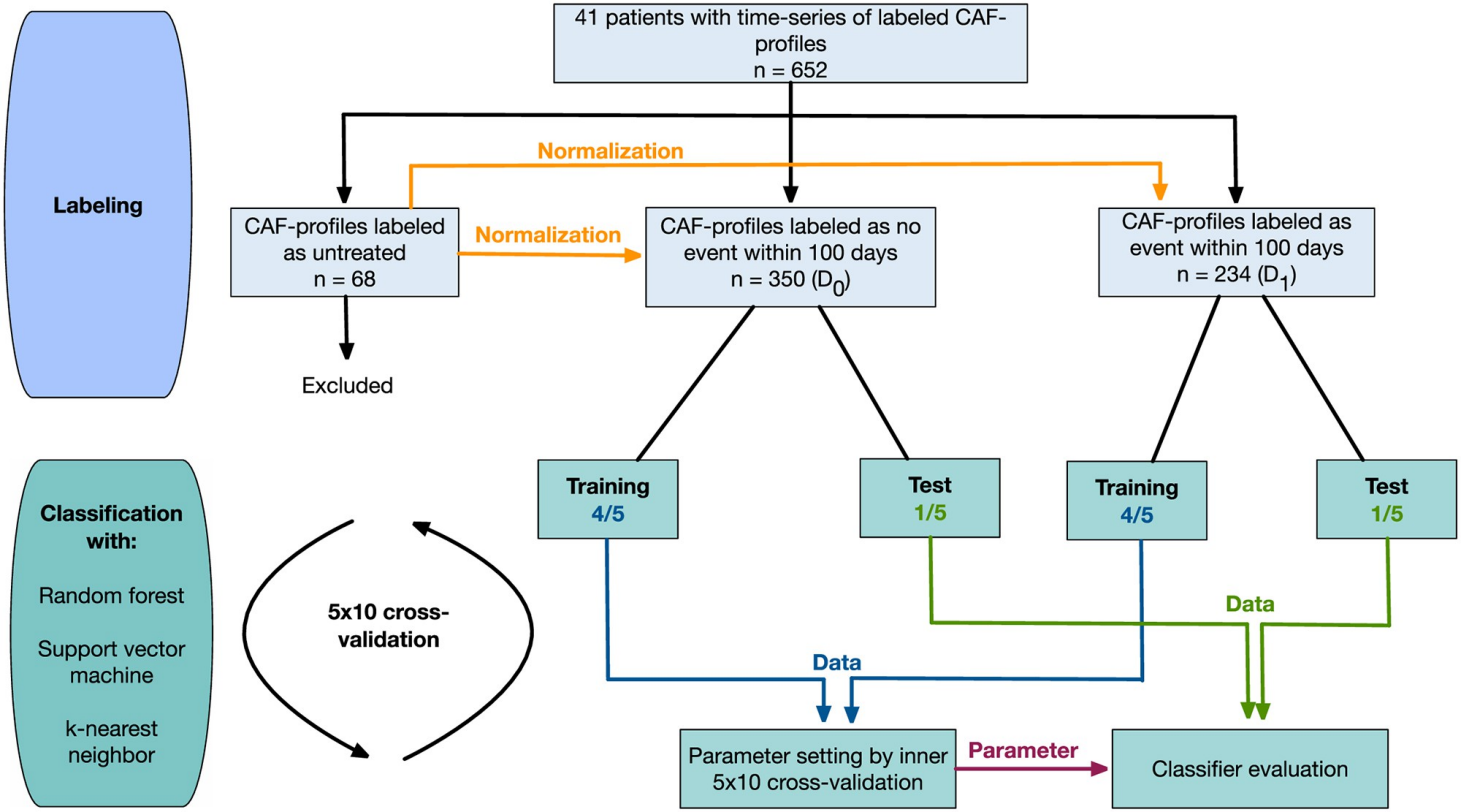
Classification workflow. CAF signatures collected before or during treatment until disease progression were normalized and designated as no event or event, respectively. For classification, we only considered the CAF signatures (n) during treatment until disease progression and performed classification experiments with the classifiers random forest, support vector machine, and k-nearest neighbor. To adjust the meta-parameters of the classifiers, we performed a nested crossvalidation (CV) solely on the training data (see Methods). Afterwards, the obtained parameters and the performance of the classifier were evaluated on the test data (outer CV).

The workflow for the biomarker identification is depicted in Fig 2. It is divided in a data preparation step, including data labeling and normlization followed by prediction. In the following, we briefly describe the workflow from data labeling to the classification. A detailed mathematical description of the prediction procedure can be found in the Supporting Information (S1 Text) together with the accompanying code (S1 Code, GitHub https://github.com/sysbio-bioinf/permad).

#### Labeling and normalization

CAF signatures collected before or during treatment until disease progression were normalized and labeled as untreated, or disease progression earlier than 100 days, or later than 100 days (S2 Table). In this analysis we are mainly interested in the performance of the prediction of an event during treatment based on an individual measurement profile. Measurement profiles assigned with a class label *untreated* were therefore excluded from further analyses. For normalization, the starting point of the untreated samples of each patient was subtracted from the other measurement profiles.

#### Evaluation measures

We analyzed the available data by training static binary classification models that receive a single measurement profile of an individual patient and return a categorial prediction, progression within 100 days or no progression within 100 days. A classification model in general is data driven and requires a set of labeled training examples for adapting the internal parameters of the model. To keep this process semi-automatic we conducted inner crossvalidation for their selection. The most important characteristic of a trained classifier is its generalization performance in correctly prediction the categories of samples that were not involved into the training process. In this work we used the following quality measures: accurarcy (Acc), sensitivity (Sen), and specificity (Spe). We also assessed their mean (SS2), which can be seen as a class-balanced version of the accuracy.

#### Parameter optimization with nested crossvalidation

In order to utilize the available dataset in an efficient way the experiments were organized in a nested crossvalidation [16] (S3 Fig). Here, crossvalidation (CV) is used for two different purposes:

1. Outer CV: Training and test sets and for classifier evaluation.
2. Inner CV: Internal parameter selection for classifier training.

Crossvalidation is a standard resampling technique generating independent training and test sets systematically. It depends on the number of runs *r* and the number of folds *f*. The overall dataset is split into *f* folds of approximately equal size and class distribution. One of these folds is used as independent test set. The remaining ones are used as a training set for the classification model. The procedure is repeated for each individual fold leading to *f* independent evaluations. It is also repeated for *r* independent permutations of the sample set in order to avoid sampling effects. Overall a set of *r* × *f* evaluations is created and reported. For our evaluation, we have chosen the values *r* = 5 and *f* = 10.

#### Classification models

We utilized as classification models random forests (RF) [17], (linear) Support Vector Machines (SVM) [18], and *k*-Nearest Neighbor Classifiers (*k*-NN) [19]. The parameter ranges are given in S3 Table. All experiments were conducted with R and the TunePareto package [20].

#### Importance ranking

In contrast to SVM and *k*-NN, RFs are feature selecting classifiers that can additionally provide an importance score for each of the 102 measurements of the overall CAF profile. The RF classifier is an ensemble classifier that aggregates the predictions of multiple decision trees (DT) for the classes (here: progress within 100 days vs. no-progress within 100 days) via a majority vote. It also aggregates the DT’s internal importance scores for each cytokine via averaging. This internal structure can be used for calculating an importance score for characterizing the individual CAFs. It is calculated as the total decrease in node impurities from splitting on the cytokine (Gini-Index). For our analysis, we calculated the importance score for each model of the 5 × 10 CV. For each of the 5 × 10 experiments the importance scores were ranked. For the overall 5 × 10 CV the CAFs were sorted according to their median rank.

## Results

### Patient characteristics

To identify biomarkers for a prediction of anti-VEGF resistance during first-line treatment of mCRC patients with mFOLFOX6 plus bevacizumab, we analyzed 50 patients with disease progress according to RECIST 1.1 during continuous first-line chemotherapy with mFOLFOX6 and bevacizumab or at least 5-FU/LV plus bevacizumab, for safety and baseline features. The baseline characteristics of these 50 patients are presented, 41 out of these 50 patients were also eligible for CAF analysis (Fig 1). The baseline characteristics of these patients are shown in Table 1.

**Table 1:** Baseline characteristics (safety set population).

Baseline characteristic	mFOLFOX6 + Bevacizumab (n = 50)
**Gender**, n (%)	
Male	28 (56.0)
Female	22 (44.0)
**Age**, mean, (range), years	61.8 (33–80)
**ECOG performance status**, n (%)	
0	26 (52.0)
1	20 (40.0)
2	2 (4.0)
**Body mass index**, median, (range), (kg/m2)	24.7 (16.0–36.3)
**Prior adjuvant Chemo- or Radiochemotherapy**, n (%)	
Yes	6 (12.0)
No	44 (88.0)
**Primary tumor resected**, n (%)	
Yes	30 (60.0)
No	20 (40.0)
**Metastasis**, n (%)	
Synchronous	38 (76.0)
Metachronous	12 (24.0)
**Differentiation grade of the tumor**, n (%)	
Well differentiated (G1)	1 (2.0)
Moderately differentiated (G2)	27 (54.0)
Poorly differentiated (G3)	14 (28.0)
Not specified (Gx)	8 (16.0)
**Localization of primary tumor**, n (%)	
Right-sided (Caecum, asc. and transv. colon)	24 (48.0)
Left-sided CRC (desc. and sigmoid colon, rectum)	26 (52.0)
**Ras-mutational status**, n (%)	
KRas/NRas mutation	34 (68.0)
KRas/NRas wild type	11 (22.0)
KRas/NRas status unknown	5 (10.0)
**B-Raf (V600E) -mutational status**, n (%)	
B-Raf V600E mutation	2 (4.0)
B-Raf wild type	15 (30.0)
B-Raf status unknown	33 (66.0)

Abbreviations: ECOG: eastern cooperative oncology group

The mean age of patients was 61.8 years. Gender was well balanced in the trial. The majority of patients had ECOG 0 (52.0%) or 1 (40.0%), respectively. Tumor grading was mostly G2 (54.0%) and G3 (28.0%). In about 60%, the primary tumor was resected. The proportion of right sided (48.0%) and left sided (52.0%) primary tumors was similar. Most patients had synchronous metastases (76.0%), a group of patients with a more unfavorable prognosis [21]. 68.0% of patients had a Ras mutated mCRC; in 10% Ras status was unknown, detection of which was not a study inclusion criterion. Systemic therapy was well tolerated, and no unexpected toxicities occurred, comparable to existing data on the combination of 5-FU/FA and oxaliplatin plus bevacizumab in the first-line treatment of mCRC. Among the 50 patients there was at least 1 adverse event of any grade in all patients: 68% experienced CTC-AEs ≥ grade 3, in particular neutropenia, leukopenia, diarrhea and stomatitis, polyneuropathy, hypertension and thromboembolic events (S4 Table). One patient died due to esophageal variceal bleeding.

**Fig 3:**
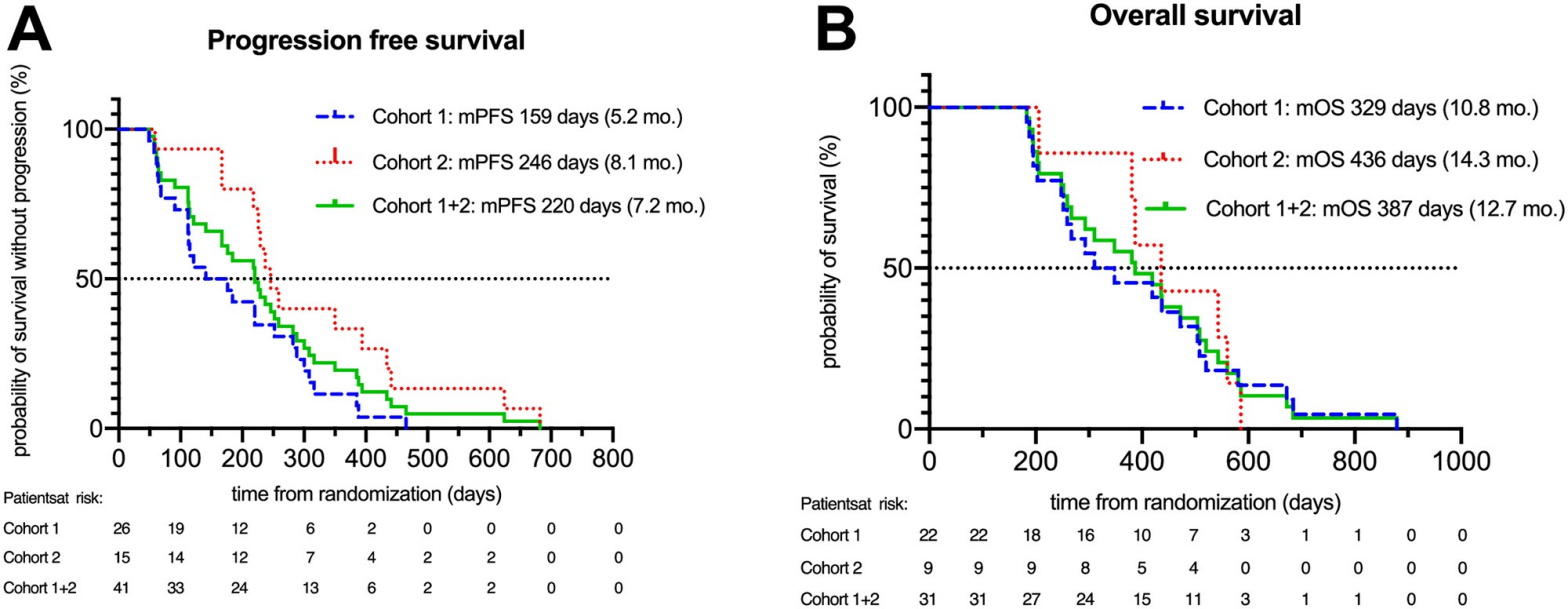
Patient characterization. Kaplan-Meier curves of progression free survival (PFS) (A) and overall survival (OS) in cohort 1, cohort 2, and both cohorts together.

Data for progression free survival (PFS, n = 41) and overall survival (OS, n = 31) (Fig 3) were evaluated in two subsequent cohorts, comprising 26 and 15 patients, respectively to expediate biomarkers analysis (PFS cohort 1: 26 patients, cohort 2: 15 patients; OS: cohort 1: 22 patients, cohort 2: 9 patients). The median progression free survival (PFS) was 220 days (7.2 months) in the whole group. Median PFS in the first cohort of 26 patients was 159 days (5.2 months) and in the second cohort of 15 patients 246 days (8.1 months). The overall response rate (complete or partial remission according to the RECIST 1.1 criteria) was 51.2% and disease control rate (stable disease, partial or complete remission according to RECIST 1.1 criteria) was 85.3% in the whole group of patients (Fig 3 and S5 Table). In the first cohort, median OS was 329 days (10.8 months) and 436 days (14.3 months) in the second cohort, respectively (Fig 3 and S5 Table).

#### Prediction model

**Fig 4:**
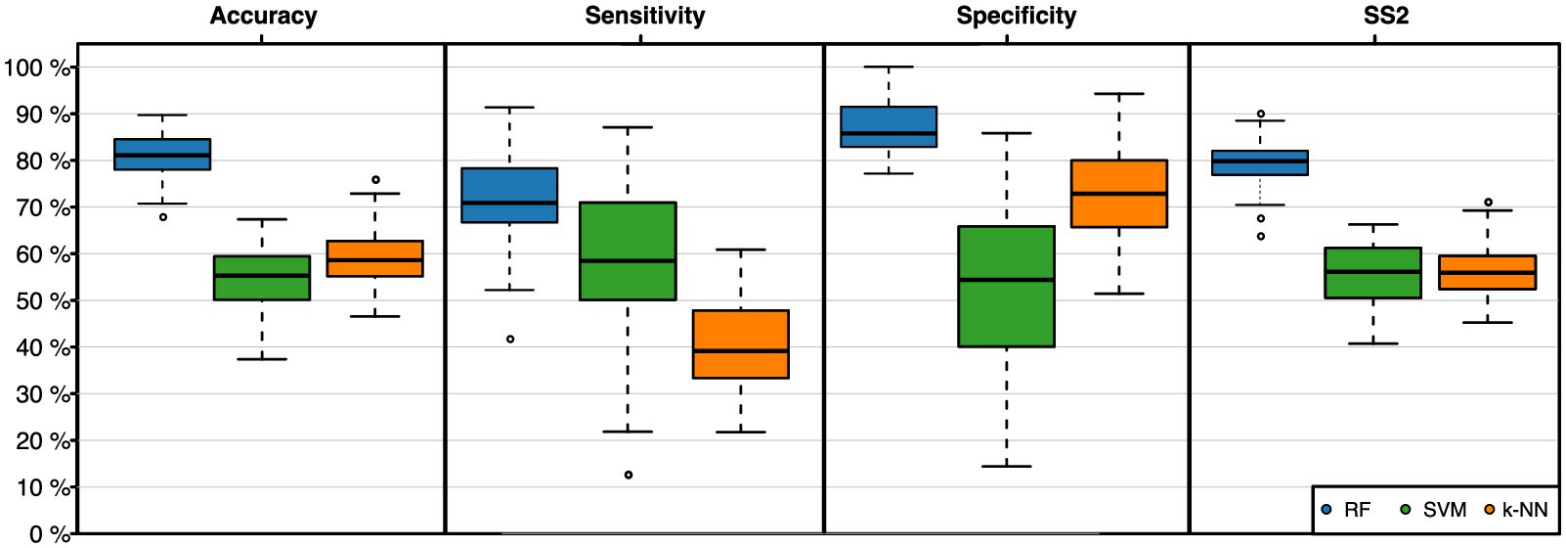
Performance measures of the classification models. Data was analyzed in 5 × 10 crossvalidation (CV) experiments. The figure shows the accuracy, sensitivity (progress within 100 days), specificity (no progress within 100 days), and SS2 achieved in the test phases of the 5 × 10 CV for the classifiers random forest (RF, blue), support vector machine (SVM, green), and k-nearest neighbor(k-NN, orange).

CT staging was performed every 60 days. Hierarchical clustering analyses indicated that our CAF markers can indeed differentiate progress from no progress within 100 days (S2 Fig). To identify biomarkers for a prediction of resistance during first-line treatment of mCRC patients with mFOLFOX6 plus bevacizumab, we performed multiple prediction experiments (Fig 2). The results are given in Table 2 and Fig 4. Within the test phase the highest mean accuracy was achieved by the RF classifier (80.8%), together with a mean sensitivity of 71.0% and a mean specificity of 87.4%. SVM (54.6%) and *k*-NN (59.3%) resulted in lower mean accuracies.

**Table 2:** Prediction performance. Performance (in %) results of the 5 × 10 crossvalidation (CV) experiment. The mean performance and the interquartile range of the training and test phases are reported.

**Training**			
**Classifier**	RF	SVM	k-NN
**Accuracy**	81.0 [80.5;81.3]	55.7 [54.9;56.3]	61.0 [60.1;62.0]
**Sensitivity**	70.6 [69.6;72.1]	56.6 [55.0;58.6]	43.3 [38.9;47.7]
**Specificity**	87.8 [87.1;88.6]	55.1 [53.4;56.9]	72.9 [69.1;77.1]
**SS2**	79.3 [78.7;79.8]	55.8 [55.1;56.4]	58.1 [57.5;58.5]

**Test**			
**Classifier**	RF	SVM	k-NN
**Accuracy**	80.8 [78.3;84.5]	54.6 [50.0;59.3]	59.3 [55.2;62.7]
**Sensitivity**	71.0 [66.7;78.3]	57.8 [50.5;70.8]	40.3 [33.3;47.8]
**Specificity**	87.4 [82.9;91.4]	52.5 [40.0;65.0]	72.0 [65.7;80.0]
**SS2**	79.2 [76.9;82.0]	55.1 [50.5;61.0]	56.1 [52.4;59.5]

**Fig 5:**
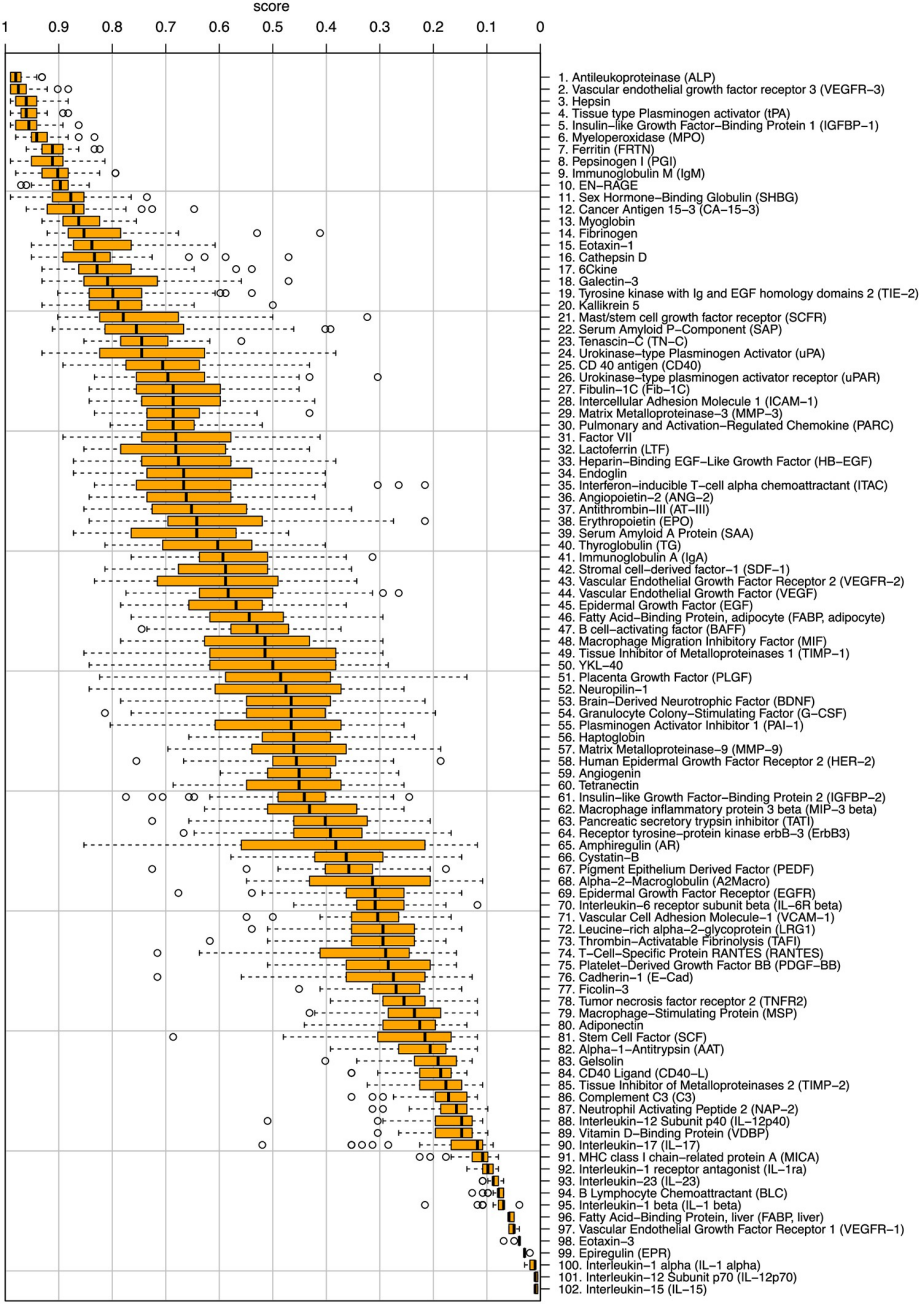
Importance ranking. The figure shows the results of the crossvalidation (CV) with the random forest (RF) classifier for the individual features within the 5 × 10 CV. The features with the highest importance can be found at the top of the figure.

**Fig 6:**
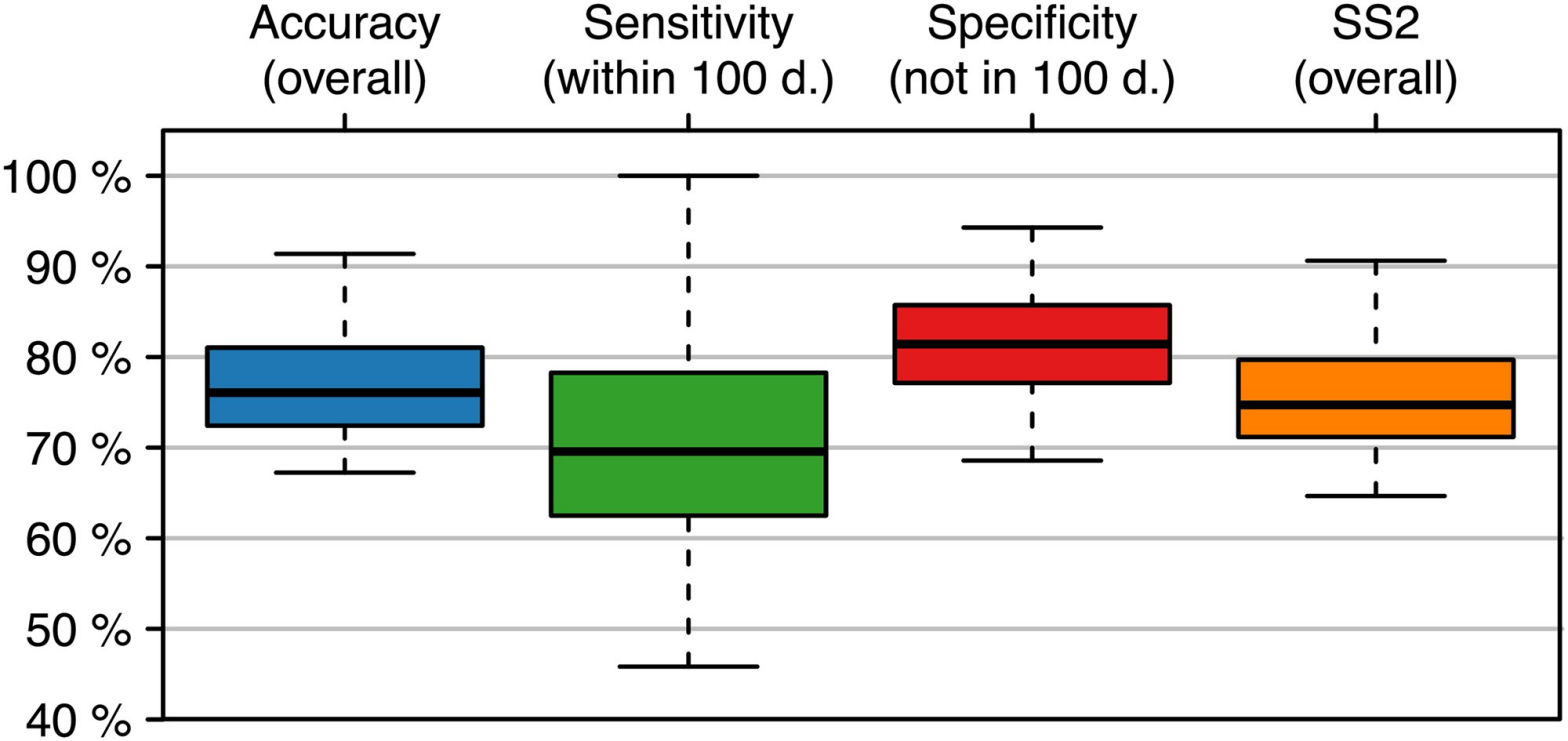
Evaluation of the top-ten signature with RFs. Accuracy, sensitivity (progression within 100 days), specificity (no progression within 100 days) and SS2 in the test phases of the 5 × 10 CV on the top-ten signature. Reclassification accuracy, i.e. training and test on the data, resulted in an accuracy of 100% for a minimum of 41 trees.

Based on these results, the random forest classifier was chosen for further analysis since it achieved the highest accuracies in the classification study. Another advantage of RF is that this ensemble classifier aggregates the predictions of multiple decision trees for the classes via majority vote. It can provide an importance ranking of the individual CAFs (Fig 5). This importance ranking highlights that only few markers seem to be relevant. This is significant as repeated analysis of a small set of markers is clinically feasible. Among the top ten markers, all achieving a mean score ≥0.9, we identified antileukoproteinase (ALP), vascular endothelial growth factor receptor 3 (VEGFR-3), hepsin, tissue type plasminogen activator (tPA), insuline-like growth factor-binding protein 1 (IGFBP-1), myeloperoxidase (MPO), pepsinogen I (PGI), immunoglobulin M (IgM), and extracellular newly receptor for advanced glycation end-products (EN-RAGE). To strengthen our initial finding and in order to define a potentially clinically useful dataset, we next analyzed the signature of the top ten markers to characterize their standalone predictive performance without additional information from the remaining features. To do so, we repeated the classification experiments described above, this time on the restricted ten feature dataset (Table 3 and Fig 6). In comparison to a classification on the entire 102 markers set, the RF classifier resulted in an accuracy of 76.7% (Sen.: 70%, Spe.: 81.2%). This demonstrates that a set of ten CAFs might be enough to indicate treatment resistance of FOLFOX plus bevacizumab within 100 days prior to radiological progress.

**Table 3:** Prediction performance based on top-10 signature. Performance of the top-10 signature (in %) in the 5 × 10 crossvalidation (CV) experiment, the mean performance and interquartile range are given.

	Training	Test
**Accuracy**	78.2 [77.6;78.7]	76.7 [72.5;80.7]
**Sensitivity**	71.8 [70.4;73.0]	70.0 [62.5;77.5]
**Specificity**	82.5 [81.7;83.7]	81.2 [77.1;85.7]
**SS2**	77.1 [76.6;77.6]	75.6 [71.2;79.7]

## Discussion

Anti-VEGF mAbs are widely used for the treatment of various cancers, including mCRC. Despite their widespread use, there are no established biomarkers that enable to predict resistance. Acting on endothelial cells and not on the tumor cells itself, it has been hypothesized that there may be no resistance to anti-VEGF treatments. Indeed, in some patients this seems to be the case at least over a long period of systemic treatment enabling a continuation of treatment beyond progression that has been demonstrated for example in the ML18147 trial [7]. Nevertheless, there are well described escape mechanisms that can occur when VEGF is blocked by mAbs and at the same time hypoxia is present leading to an increase in pro-angiogenic factors such as PLGF, PDGF, and others that substitute for VEGF in the angiogenic process [9, 11, 12]. Thus, markers that can predict resistance to an anti-VEGF treatment in an individual patient could enable a timely initiation of treatments with broader acting antiangiogenic agents and potentially restore anti-angiogenic treatment efficacy or other targeting strategies. Various approaches have been used to identify such biomarkers, but so far, the results were not convincing enough to be put into clinical practice [9, 11, 12]. Here, we used an approach that appreciates the complexity of the biomarker analysis in the field of anti-angiogenic treatment by employing random forest machine learning to identify a biomarker class that allow an early prediction of progression. From a theoretical perspective, the design of a predictive model based on monitoring of a low-dimensional panel is a non-trivial task. Although our analysis was guided by a preselected panel of CAF-markers, the corresponding measurements might be influenced by personal or temporal differences as they occur during the longitudinal monitoring of different patients [9]. However, the possibilities of isolating these foreign signals are limited due to PERMAD being a real world trial with rather broad inclusion criteria. Thus, the conditional distributions of individual patients might not be perfectly aligned leading to a disrupted and disconnected patterns. As stipulated in the protocol, 50 patients had to fulfill the main criteria of having received at least 5-FU plus bevacizumab continuously until disease progression. Patients with extended periods without systemic/anti-angiogenic treatment due to drug holidays, local interventions, metastasis surgery or drug induced adverse events prohibiting further treatment were not considered for the biomarker analysis. This may constitute a certain bias. CAF determination was done in two cohorts. Progression free survival was shorter in the first cohort (5.2 months) than in the second cohort (8.1 months) which is explained by the fact that patients with the shortest PFS were analyzed in the first cohort. The median PFS of both cohorts was 7.2 months which is shorter compared to other studies such as TRIBE2 with a PFS in the dual combination plus bevacizumab arm of 9.2 months [22]. However, this could be explained by the high dropout rate of patients with favorable treatment results, e.g. receiving drug holidays or interventions which is usually a more favorable population of patients with mCRC [7, 23].

To establish a predictive biomarker categorization, we used a 102-marker panel (S1 Table) that was repeatedly assessed in patients prior to and during the course of a combination of mFOLFOX6 and bevacizumab until disease progression and divided the patients in two groups according to the time of disease progression. The 100-day period is supported by an initial unsupervised analysis of the data (S2 Fig) and also being a clinically meaningful period of time for an early change of treatment.

Using three different approaches, we identified markers that allow to predict disease progress during treatment of 5-FU plus bevacizumab within 100 days prior to radiological progress (Table 2). While the random forests achieved a mean accuracy of 80.8%, the linear support vector machines (54.6%) and the *k*-nearest neighbor classifiers (59.3%) showed an inferior performance that did not pass the baseline accuracy (59.9%) of the experimental setup (for a detailed discussion about the performance of the classifiers see S2 Text). This could be due to the highly individual disease progression which is less likely to be captured by the SVM or the kNN.

Since a prediction tool with few markers is more manageable in clinical routine, we next tested if a smaller set of CAF markers can still differentiate between progression or no progression within 100 days during treatment of 5-FU plus bevacizumab. This focus on a specific subset of CAFs is motivated by an evaluation of the inherent importance ranking of the random forests classifiers (Fig 5). Here, a preference for certain CAFs is observed. A re-evaluation of the random forest classifiers restricted to the top-10 CAFs showed that those markers resulted in an accuracy of 78.2% (Table 3). Among the top ten CAFs, antileukoproteinase (ALP) and vascular endothelial growth factor receptor 3 (VEGFR-3), are both known to regulate NF-*κ*B signaling thereby affecting immune responses and growth of CRC [24, 25]. In a previous study, high ALP was associated with shorter overall survival of mCRC patients [24]. Thus, both might play a role in early disease progression. Similarly, high levels of tissue type plasminogen activator (tPA) as well as high expression of hepsin (HPN) have previously been associated with an increased risk of CRC recurrence and shortened PFS [26, 27]. In a similar setting to our trial with aflibercept plus 5‐fluorouracil/levofolinate/irinotecan (FOLFIRI) treated mCRC patients, Hamaguchi et al. [28] also identified EN-RAGE as potential biomarker with lower EN-RAGE plasma concentration than the median as favorable for the overall survival. In contrast, IgM autoantibodies were associated with improved CRC patient survival as well as an early-stage detection of CRC [29]. Interestingly, despite the relative gender balance in our cohort, insulin-like growth factor-binding protein 1 (IGFBP-1) was previously associated with increased CRC risk in women [30]. One reason for these different findings might be the age differences between the study cohorts. In line with disease progression under treatment with mFOLFOX6 plus bevacizumab, high myeloperoxidase (MPO) levels have been correlated with malignant progression and survival of CRC patients also by others [31]. To sum up, the biological role of our identified top ten CAF markers further support their role as potential indicators for treatment resistance.

## Conclusion

Numerous national and international guidelines recommend first-line treatment of mCRC patients with FOLFOX plus bevacizumab. While some patients initially benefit from this treatment, resistances can develop. However, to date there are no biomarkers available predicting this resistance. Here, we describe the establishment of a prediction model and a CAF-signature that indicates treatment resistance to mFOLFOX6 plus bevacizumab in mCRC patients within 100 days prior to conventional staging by radiological imaging. It will be important to test prospectively whether an early switch to another anti-angiogenic treatment based on this marker combination can improve the outcome of mCRC patients, particularly those with early disease progression.

## Supporting information

S1 Fig Sample size estimation. Assuming an observed classification performance of 0.85 and a 95% confidence interval of 0.75–0.95, a sample size of n = 49 was calculated using the Wald confidence interval.
(PDF)

S2 Fig Hierarchical clustering. Hierarchical cluster analysis (Ward method) of the time course of the cytokine profiles from one patient demonstrating that the time series of cytokine profiles can be split into “early” and “late” profiles according to a patient specific threshold about 3–4 months before radiological progress. The depicted case example shows a threshold of about 100 days.
(PDF)

S3 FigNested crossvalidation scheme for parameter optimization.The figure outlines the resampling strategy for the use of the samples in datasets D. On the outer level D is split into 5 folds $F_{1},\ldots ,F_{5}$. Each of these folds is used once as a test set $D_{te}$ while the other are jointly used as a training set $D_{tr}$. The procedure is repeated on 10 permutations of D. On an inner level the same procedure is applied to the samples of the current training set $D_{tr}$ resulting in inner folds $I_{1},\ldots ,I_{5}$. This split is used for internal parameter selection.(PDF)

S1 Table Biomarkers analyzed. (PDF)

S2 TableLabel distribution and baseline classification.The table provides the amount of samples per class and the baseline accuracy of the labeled dataset D.(PDF)

S3 Table Parameter ranges for classifier training. The table lists the parameters that were optimized during the training of a classification model. Additionally the parameter ranges are shown.
(PDF)

S4 Table Adverse events of interest (safety population) according to CTC-AE V 4.03. (PDF)

S5 Table Efficacy and best confirmed overall tumor response according to RECIST 1.1. (PDF)

S1 Text Mathematical methodology description. (PDF)

S2 Text Performance of the classifier discussion. (PDF)

S1 Data CAF signatures in blood samples of cohort 1. (XLSX)

S2 Data CAF signatures in blood samples of cohort 2. (XLSX)

S1 Code The R code used to perform the experiments. (R)

S1 Checklist TREND statement checklist. (PDF)

S1 Protocol (PDF)
