# Supplementary figures and images for: Prediction of resistance to bevacizumab plus FOLFOX in metastatic colorectal cancer—Results of the prospective multicenter PERMAD trial
Source: PLoS One. 2024 Jun 14;19(6):e0304324. doi: 10.1371/journal.pone.0304324 (PMC11178165; doi:10.1371/journal.pone.0304324)

# Wald-type confidence intervals for p

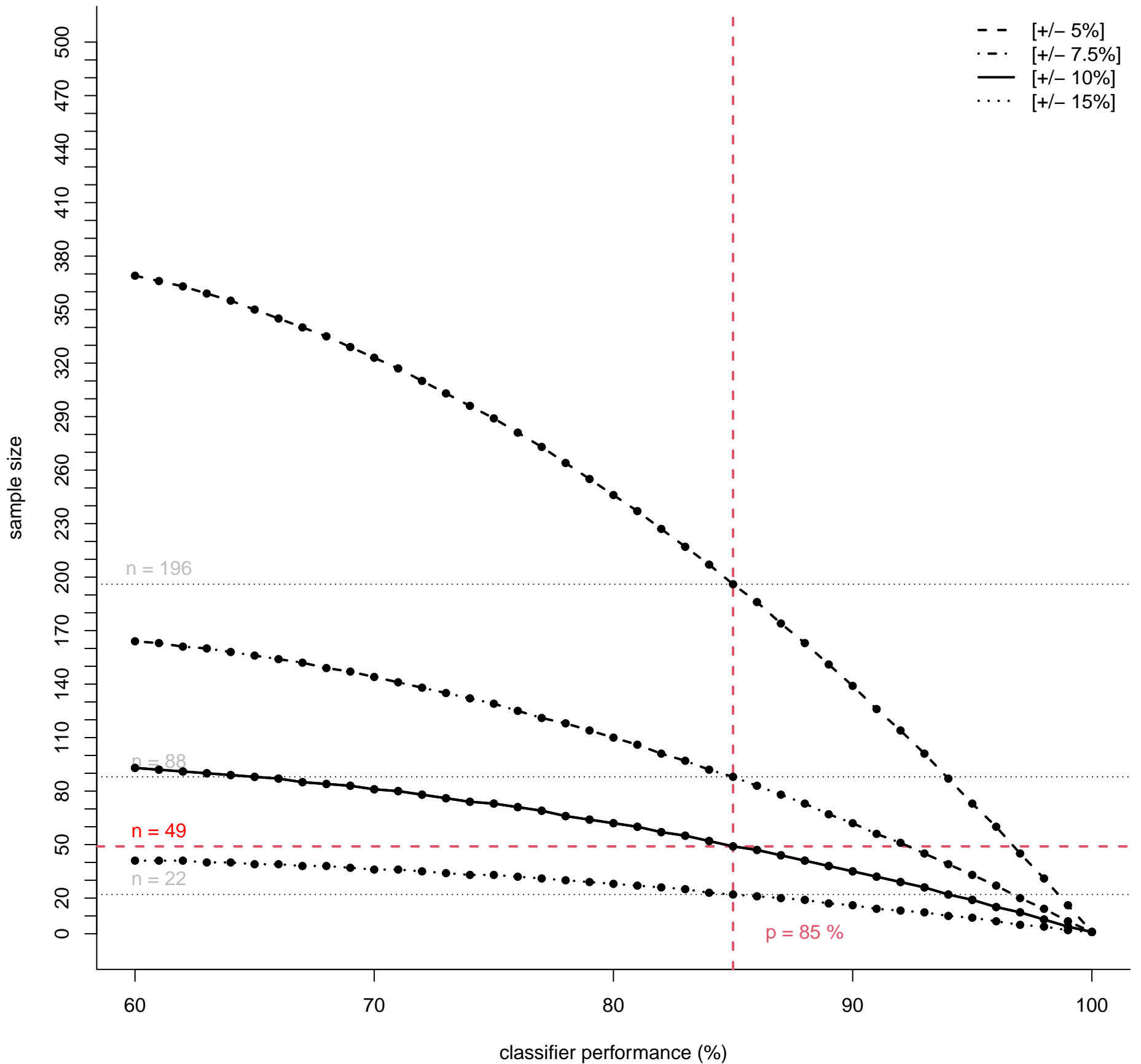

Supplement: S1 Fig — Assuming an observed classification performance of 0.85 and a 95% confidence interval of 0.75–0.95, a sample size of n = 49 was calculated using the Wald confidence interval. (PDF) [file pone.0304324.s001.pdf]

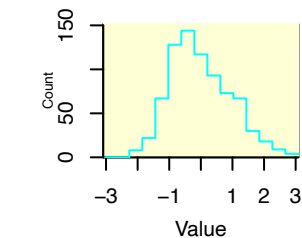

# patient 19

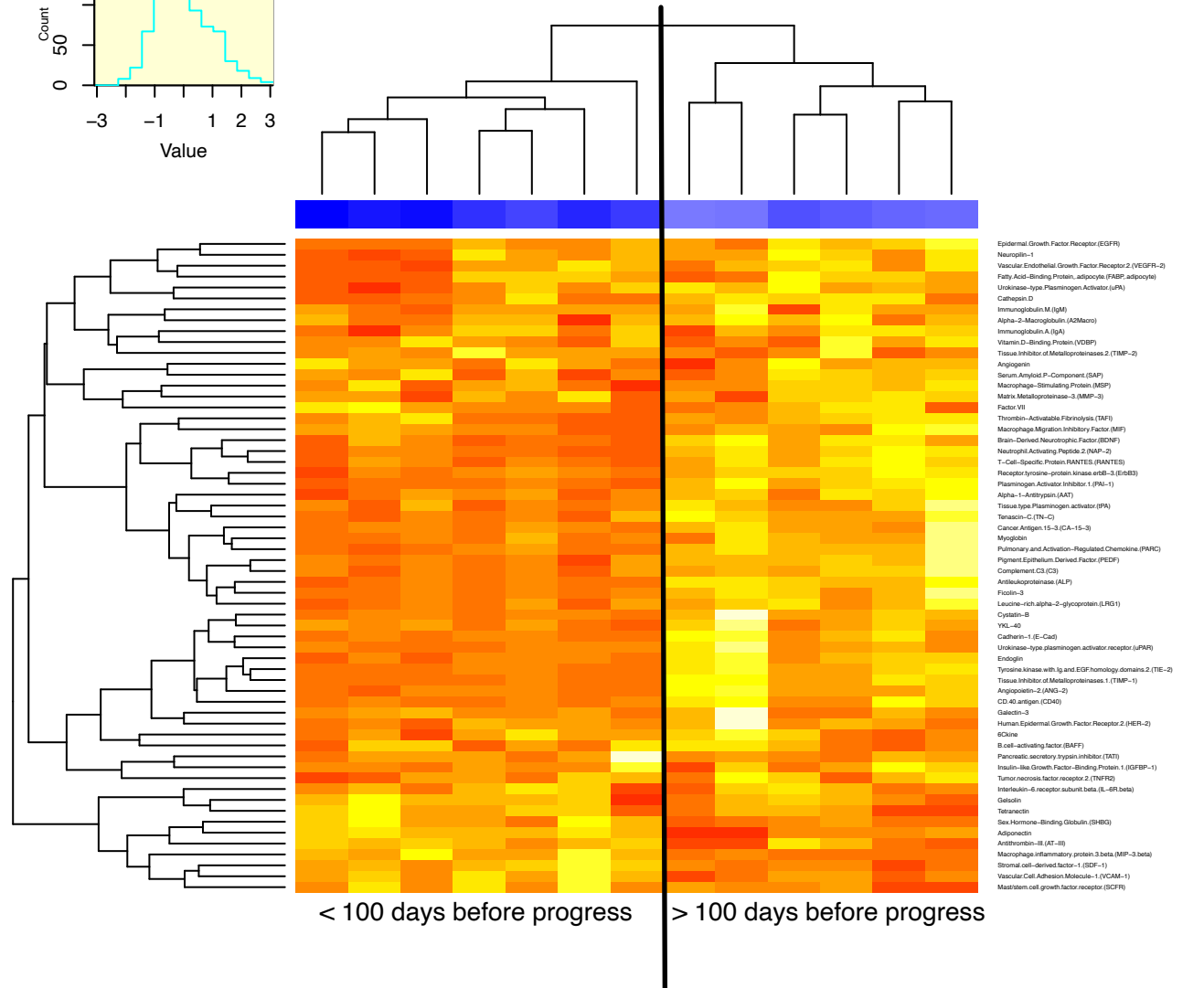

Supplement: S2 Fig — Hierarchical cluster analysis (Ward method) of the time course of the cytokine profiles from one patient demonstrating that the time series of cytokine profiles can be split into “early” and “late” profiles according to a patient specific threshold about 3–4 months before radiological progress. The depicted case example shows a threshold of about 100 days. (PDF) [file pone.0304324.s002.pdf]
