# Supplementary material for: Prediction of resistance to bevacizumab plus FOLFOX in metastatic colorectal cancer—Results of the prospective multicenter PERMAD trial
Source: PLoS One. 2024 Jun 14;19(6):e0304324. doi: 10.1371/journal.pone.0304324 (PMC11178165; doi:10.1371/journal.pone.0304324)
Supplement: S3 Fig — The figure outlines the resampling strategy for the use of the samples in datasets D. On the outer level D is split into 5 folds F1,…,F5. Each of these folds is used once as a test set Dte while the other are jointly used as a training set Dtr. The procedure is repeated on 10 permutations of D. On an inner level the same procedure is applied to the samples of the current training set Dtr resulting in inner folds I1,…,I5. This split is used for internal parameter selection. (PDF) [file pone.0304324.s003.pdf]

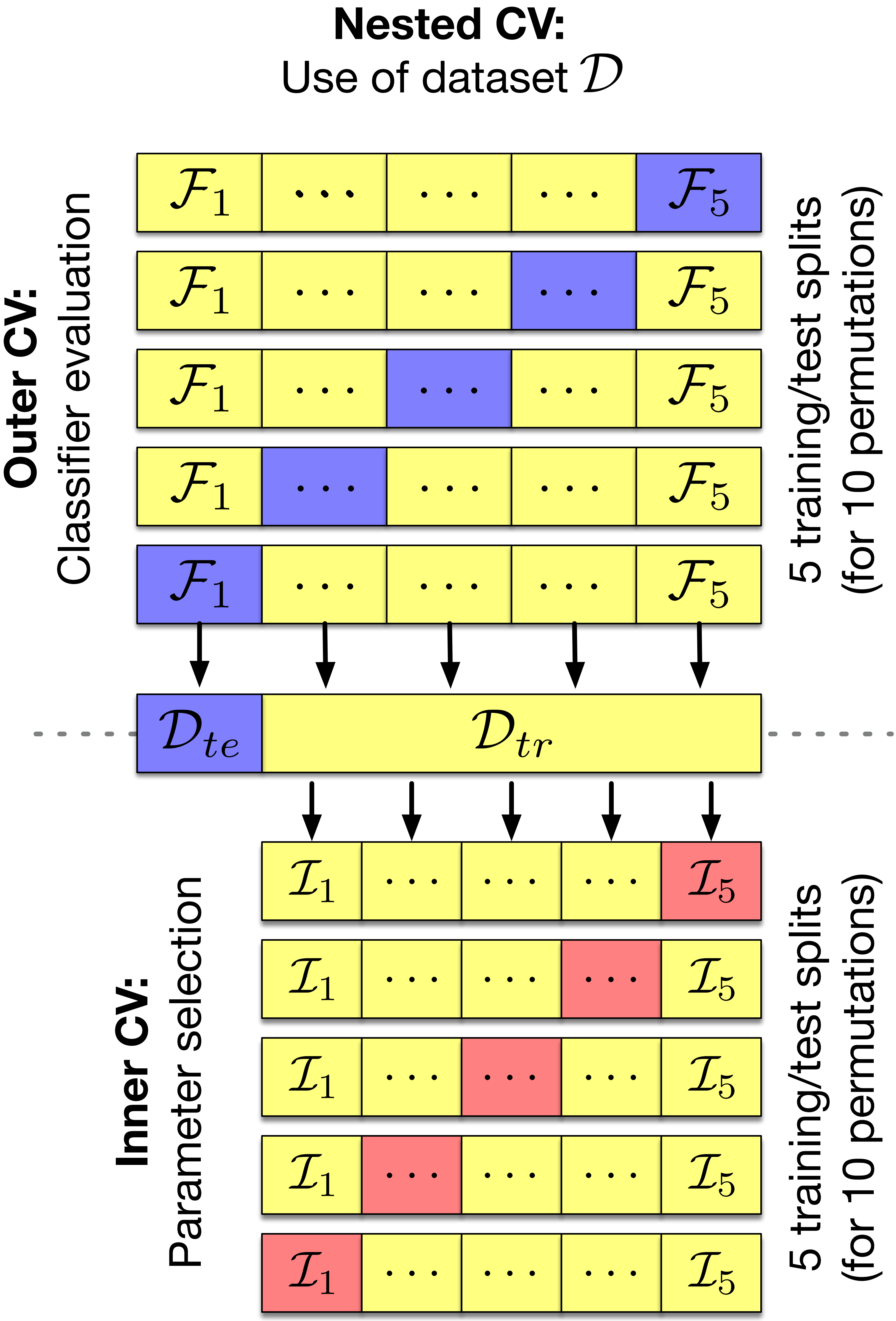

Nested CV:
Use of dataset $\mathcal{D}$
Outer CV:
Classifier evaluation
$\mathcal{F}_1$ ... ... $\mathcal{F}_5$
5 training/test splits
(for 10 permutations)
$\mathcal{D}_{te}$
$\mathcal{D}_{tr}$
Inner CV:
Parameter selection
$\mathcal{I}_1$ ... ... $\mathcal{I}_5$
5 training/test splits
(for 10 permutations)
