# Supplementary material for: Prediction of resistance to bevacizumab plus FOLFOX in metastatic colorectal cancer—Results of the prospective multicenter PERMAD trial
Source: PLoS One. 2024 Jun 14;19(6):e0304324. doi: 10.1371/journal.pone.0304324 (PMC11178165; doi:10.1371/journal.pone.0304324)
Supplement: S1 Table — (PDF) [file pone.0304324.s004.pdf]

Biomarker	
1. 6Ckine (CCL21)	52. Interleukin-12 Subunit p40 (IL-12p40)
2. Adiponectin	53. Interleukin-12 Subunit p70 (IL-12p70)
3. Alpha-1-Antitrypsin (AAT)	54. Interleukin-15 (IL-15)
4. Alpha-2-Macroglobulin (A2Macro)	55. Interleukin-17 (IL-17)
5. Amphiregulin (AR)	56. Interleukin-23 (IL-23)
6. Angiogenin	57. Kallikrein 5
7. Angiopoietin-2 (ANG-2)	58. Lactoferrin (LTF)
8. Antileukoproteinase (ALP)	59. Leucine-rich alpha-2-glycoprotein (LRG1)
9. Antithrombin-III (AT-III)	60. Macrophage inflammatory protein 3 beta (MIP-3 beta)
10. B cell-activating factor (BAFF)	61. Macrophage Migration Inhibitory Factor (MIF)
11. B Lymphocyte Chemoattractant (BLC)	62. Macrophage-Stimulating Protein (MSP)
12. Brain-Derived Neurotrophic Factor (BDNF)	63. Mast/stem cell growth factor receptor (SCFR)
13. Cadherin-1 (E-Cad)	64. Matrix Metalloproteinase-3 (MMP-3)
14. Cancer Antigen 15-3 (CA-15-3)	65. Matrix Metalloproteinase-9 (MMP-9)
15. Cathepsin D	66. MHC class I chain-related protein A (MICA)
16. CD 40 antigen (CD40)	67. Myeloperoxidase (MPO)
17. CD40 Ligand (CD40-L)	68. Myoglobin
18. Complement C3 (C3)	69. Neuropilin-1
19. Cystatin-B	70. Neutrophil Activating Peptide 2 (NAP-2)
20. EN-RAGE	71. Pancreatic secretory trypsin inhibitor (TATI)
21. Endoglin	72. Pepsinogen I (PGI)
22. Eotaxin-1	73. Pigment Epithelium Derived Factor (PEDF)
23. Eotaxin-3	74. Placenta Growth Factor (PLGF)
24. Epidermal Growth Factor (EGF)	75. Plasminogen Activator Inhibitor 1 (PAI-1)
25. Epidermal Growth Factor Receptor (EGFR)	76. Platelet-Derived Growth Factor BB (PDGF-BB)
26. Epiregulin (EPR)	77. Pulmonary and Activation-Regulated Chemokine (PARC)
27. Erythropoietin (EPO)	78. Receptor tyrosine-protein kinase erbB-3 (ErbB3)
28. Factor VII	79. Serum Amyloid A Protein (SAA)
29. Fatty Acid-Binding Protein, adipocyte (FABP, adipocyte)	80. Serum Amyloid P-Component (SAP)
30. Fatty Acid-Binding Protein, liver (FABP, liver)	81. Sex Hormone-Binding Globulin (SHBG)
31. Ferritin (FRTN)	82. Stem Cell Factor (SCF)
32. Fibrinogen	83. Stromal cell-derived factor-1 (SDF-1)
33. Fibulin-1C (Fib-1C)	84. T-Cell-Specific Protein RANTES (RANTES)
34. Ficolin-3	85. Tenascin-C (TN-C)
35. Galectin-3	86. Tetranectin
36. Gelsolin	87. Thrombin-Activatable Fibrinolysis (TAFI)
37. Granulocyte Colony-Stimulating Factor (G-CSF)	88. Thyroglobulin (TG)
38. Haptoglobin	89. Tissue Inhibitor of Metalloproteinases 1 (TIMP-1)
39. Heparin-Binding EGF-Like Growth Factor (HB-EGF)	90. Tissue Inhibitor of Metalloproteinases 2 (TIMP-2)
40. Hepsin	91. Tissue type Plasminogen activator (tPA)
41. Human Epidermal Growth Factor Receptor 2 (HER-2)	92. Tumor necrosis factor receptor 2 (TNFR2)
42. Immunoglobulin A (IgA)	93. Tyrosine kinase with Ig and EGF homology domains 2 (TIE-2)
43. Immunoglobulin M (IgM)	94. Urokinase-type Plasminogen Activator (uPA)
44. Insulin-like Growth Factor-Binding Protein 1 (IGFBP-1)	95. Urokinase-type plasminogen activator receptor (uPAR)
45. Insulin-like Growth Factor-Binding Protein 2 (IGFBP-2)	96. Vascular Cell Adhesion Molecule-1 (VCAM-1)
46. Intercellular Adhesion Molecule 1 (ICAM-1)	97. Vascular Endothelial Growth Factor (VEGF)
47. Interferon-inducible T-cell alpha chemoattractant (ITAC)	98. Vascular Endothelial Growth Factor Receptor 1 (VEGFR-1)
48. Interleukin-1 alpha (IL-1 alpha)	99. Vascular Endothelial Growth Factor Receptor 2 (VEGFR-2)
49. Interleukin-1 beta (IL-1 beta)	100. Vascular endothelial growth factor receptor 3 (VEGFR-3)
50. Interleukin-1 receptor antagonist (IL-1ra)	101. Vitamin D-Binding Protein (VDBP)
51. Interleukin-6 receptor subunit beta (IL-6R beta)	102. YKL-40
