# Supplementary material for: Prediction of resistance to bevacizumab plus FOLFOX in metastatic colorectal cancer—Results of the prospective multicenter PERMAD trial
Source: PLoS One. 2024 Jun 14;19(6):e0304324. doi: 10.1371/journal.pone.0304324 (PMC11178165; doi:10.1371/journal.pone.0304324)
Supplement: S2 Table — The table provides the amount of samples per class and the baseline accuracy of the labeled dataset D. (PDF) [file pone.0304324.s005.pdf]

classes	label y	dataset $\mathcal{D}_y$	samples $\lvert\mathcal{D}\rvert$
event	1	$\mathcal{D}_1$	234
no event	0	$\mathcal{D}_0$	350
total	$\{0,1\}$	$\mathcal{D}$	584
baseline (accuracy)			350/584 = 59.9%
