# Supplementary material for: Prediction of resistance to bevacizumab plus FOLFOX in metastatic colorectal cancer—Results of the prospective multicenter PERMAD trial
Source: PLoS One. 2024 Jun 14;19(6):e0304324. doi: 10.1371/journal.pone.0304324 (PMC11178165; doi:10.1371/journal.pone.0304324)
Supplement: S3 Table — The table lists the parameters that were optimized during the training of a classification model. Additionally the parameter ranges are shown. (PDF) [file pone.0304324.s006.pdf]

algorithm	parameters	ranges
Random Forest (RF)	ntree	25, 50, . . ., 2000
	node size	1, 2, . . ., 5
	class weight	0.05, 0.1, . . ., 0.95
k-Nearest Neighbor (k-NN)	number of neighbors	1, 3, . . ., 7
Support Vector Machine (SVM)	cost	0.01, 0.1, . . ., 100
