# Supplementary material for: Prediction of resistance to bevacizumab plus FOLFOX in metastatic colorectal cancer—Results of the prospective multicenter PERMAD trial
Source: PLoS One. 2024 Jun 14;19(6):e0304324. doi: 10.1371/journal.pone.0304324 (PMC11178165; doi:10.1371/journal.pone.0304324)
Supplement: S4 Table — (PDF) [file pone.0304324.s007.pdf]

Event (%)	mFOLFOX6 + Bevacizumab (n=50)	
**CTC-AE V 4.03**	**Any grade**	**Grade ≥3**
**Any adverse event**	**100.0**	**68.0**
**Blood system disorders**	**100.0**	**94.0**
Neutropenia	62.0	56.0
Thrombocytopenia	26.0	2.0
Leukopenia	28.0	16.0
Anaemia	24.0	4.0
Febrile neutropenia	6.0	4.0
**Cardiac disorders**	**2.0**	**0.0**
**Ear and labyrinth disorders**	**8.0**	**2.0**
Vertigo	8.0	2.0
**Eye disorders**	**4.0**	**2.0**
**Gastrointestinal disorders**	**92.0**	**48.0**
Nausea	62.0	0.0
Diarrhoea	60.0	16.0
Stomatitis	30.0	12.0
Vomiting	14.0	0.0
Colon perforation	2.0	2.0
Lower GI-bleeding	2.0	2.0
Ileus	2.0	2.0
Esophageal varices bleeding	2.0	2.0*
**General disorders**	**74.0**	**26.0**
Fatigue	54.0	8.0
Pyrexia	16.0	0.0
Pain	10.0	6.0
**Hepatobiliary disorders**	**4.0**	**0.0**
**Immune system disorders**	**4.0**	**2.0**
Drug hypersensitivity	4.0	0.0
**Infections and infestations**	**46.0**	**6.0**
Urinary tract infection	10.0	0.0
Pneumonia	8.0	4.0
**Investigations**	**58.0**	**10.0**
ALT increased	2.0	0.0
AST increased	2.0	0.0
GGT increased	2.0	2.0
Blood bilirubin increased	2.0	2.0
Weight decreased	36.0	2.0
Weight increased	2.0	2.0
False positive investigation results**	2.0	2.0
**Nervous system disorders**	**96.0**	**18.0**
Polyneuropathy	82.0	16.0
**Renal and urinary disorders**	**20.0**	**10.0**
Akute kidney injury	6.0	6.0
Proteinuria	6.0	2.0
**Respiratory disorders**	**36.0**	**2.0**
Dyspnoea	12.0	0.0
Epistaxis	12.0	0.0
Dysphonia	8.0	0.0
**Skin disorders**	**34.0**	**2.0**
Alopecia	20.0	2.0
Dry Skin	4.0	0.0
**Vascular disorders**	**50.0**	**30.0**
Hypertension	34.0	14.0
Thromboembolic event	6.0	14.0

Adverse event terms were derived from the case report forms.

All events reported irrespective of whether they were reported as related to study-treatment: *grade 5 event; ** Pseudothrombocytopenia; Abbreviations: ALT: Alanine aminotransferase, AST: Aspartate aminotransferase, GGT: Gamma-glutamyltransferase
