# Supplementary material for: Prediction of resistance to bevacizumab plus FOLFOX in metastatic colorectal cancer—Results of the prospective multicenter PERMAD trial
Source: PLoS One. 2024 Jun 14;19(6):e0304324. doi: 10.1371/journal.pone.0304324 (PMC11178165; doi:10.1371/journal.pone.0304324)
Supplement: S5 Table — (PDF) [file pone.0304324.s008.pdf]

**Results**	**Cohort 1** mFOLFOX6 + Bevacizumab	**Cohort 2** mFOLFOX6 + Bevacizumab	**Cohort 1+2** mFOLFOX6 + Bevacizumab
**Median PFS** (months)	5.2 (n=26)	8.2 (n=15)	7.2 (n=41)
**Median OS** (months)	10.8 (n=22)	14.3 (n=9)	12.7 (n=31)
**Overall RR** (%)	42.3	66.7	51.2
**Disease control rate** (%)	80.8	93.4	85.3
**Stable disease**, n (%)	10 (38.5)	4 (26.7)	14 (34.1)
**Partial remission**, n (%)	10 (38.5)	10 (66.7)	20 (48.8)
**Complete remission**, n (%)	1 (3.8)	0 (0.0)	1 (2.4)
**Progressive disease**, n (%)	5 (19.2)	1 (6.7)	6 (14.6)

Abbreviations: PFS: progression free survival, OS: overall survival, RR: response rate, mFOLFOX6: fluorouracil, folinic acid and oxaliplatin
