# Supplementary material for: Prediction of resistance to bevacizumab plus FOLFOX in metastatic colorectal cancer—Results of the prospective multicenter PERMAD trial
Source: PLoS One. 2024 Jun 14;19(6):e0304324. doi: 10.1371/journal.pone.0304324 (PMC11178165; doi:10.1371/journal.pone.0304324)
Supplement: S1 Text — (PDF) [file pone.0304324.s009.pdf]

S1 Text. Mathematical methodology description

The following section describes the setup for the conducted (static) classification and biomarker selection experiments in detail. It comprises the data preparation and normalization steps applied for transforming time series data into static data. After that the evaluation measures and the overall evaluation strategy are provided. The section ends with an overview on the applied classification models and biomarker selection algorithms. As we do not assume an effect of the inclusion period, all experiments were conducted on the combined dataset of both cohorts.

Data preparation and normalization

The subsequent section provides the preparation steps applied to transform the labeled time series data ($\mathcal{S}$) into a labeled dataset ($\mathcal{D}$) for static classification experiments and biomarker selection.

Let $\mathbf{x} = (x^{(1)}, \ldots, x^{(n)})^T \in \mathcal{X}$ denote a single measurement profile for an individual patient $p \in \mathcal{P}$ and an individual time point t. The original recordings for patient p are organized in form of a labeled sequence S_p of length l_p. More precisely

$$S_p = \big((\mathbf{x}_t, y_t)\big)_{t=1}^{l_p} \quad \text{with labels } y_t = \begin{cases} -1 & \text{untreated} \\ 0 & \text{no event within 100 days} \\ +1 & \text{event within 100 days} \end{cases}$$

and $y_t \leq y_{t+1}$ for all t. The original dataset is therefore given by collection of labeled time series $\mathcal{S} = \{S_p\}_{p \in \mathcal{P}}$.

In this analysis we are mainly interested in the performance of the prediction of an event during treatment ($\mathcal{Y} = \{0, 1\}$) based on an individual measurement profile $\mathbf{x} \in \mathcal{X}$. Measurement profiles assigned with a class label $y = -1$ are therefore excluded as direct training samples. However, they are used for normalization. Let $\mathbf{m}_p$ denote the centroid of the untreated samples

of a patient p

$$\mathbf{m}_p = \frac{1}{|\mathcal{M}_p|} \sum_{\mathbf{x} \in \mathcal{M}_p} \mathbf{x} \quad \text{, where} \quad \mathcal{M}_p = \{\mathbf{x} | (\mathbf{x}, y) \in S_p, y = -1\} \,. \tag{1}$$

The labeled dataset $\mathcal{D}$ for static classification algorithms is constructed as follows

$$\mathcal{D} = \big\{(\mathbf{x} - \mathbf{m}_p, y) | p \in \mathcal{P}, (\mathbf{x}, y) \in S_p, y \neq -1\big\}. \tag{2}$$

For simplification we will use $\mathcal{D} = \{(\mathbf{x}_i, y_i)\}_{i=1}^{|\mathcal{D}|}$ to denote the normalized samples. An overview on the samples in $\mathcal{D}$ is given in Table S2.

Evaluation measures

We analyzed the available data by training static binary classification models of type

$$c : \mathcal{X} \rightarrow \mathcal{Y} \tag{3}$$

that receive a single measurement profile $\mathbf{x} \in \mathcal{X}$ (of an individual patient) and return a categorial prediction $y \in \mathcal{Y} = \{0, 1\}$. In our context a prediction of an event in 100 days is encoded as $y = 1$ and a prediction of no event in 100 days was encoded as $y = 0$.

The exact training process of a classification model depends on the chosen type of classifier. It is in general data driven and requires a set of labeled training examples $\mathcal{D}_{tr} = \{(\mathbf{x}_i, y_i)\}_{i=0}^{m}$ for adapting the internal parameters of the model. However most classification algorithms also provide a set of meta-parameters that have to be specified by the user. To keep this process semi-automatic we conducted inner crossvalidation experiments for their selection.

The most important characteristic of a trained classifier is its generalization performance in correctly prediction the categories of samples $\mathcal{D}_{te}$ that were not involved into the training process. It is typically estimated in form of rates of type

$$\mathrm{Emp}(c, \mathcal{D}) = \frac{1}{|\mathcal{D}|} \sum_{(\mathbf{x}, y) \in \mathcal{D}} \mathbb{I}_{[c(\mathbf{x}) = y]} \,. \tag{4}$$

In this work we evaluate following quality measures:

$$\text{Accuracy: } \mathrm{Acc}(c, \mathcal{D}) = \mathrm{Emp}(c, \mathcal{D}) \quad (5)$$

$$\text{Sensitivity: } \mathrm{Sen}(c, \mathcal{D}) = \mathrm{Emp}(c, \mathcal{D}_1) \quad (6)$$

$$\text{Specificity: } \mathrm{Spe}(c, \mathcal{D}) = \mathrm{Emp}(c, \mathcal{D}_0) \quad (7)$$

$$\text{Mean between Sen and Spe (SS2): } \mathrm{SS2}(c, \mathcal{D}) = (\mathrm{Sen}(c, \mathcal{D}) + \mathrm{Spe}(c, \mathcal{D}))/2 \quad (8)$$

Here $\mathcal{D}_y \subseteq \mathcal{D}$ denotes a subset of $\mathcal{D}$ that comprises only samples of class y

$$\mathcal{D}_y = \{x \mid (\mathbf{x}, y') \in \mathcal{D}, y' = y\}. \quad (9)$$

Since sensitivity and specificity are competing objectives, we also assessed their mean (SS2), which can be seen as a class-balanced version of the accuracy.

Parameter optimization with nested crossvalidation

In order to utilize the available dataset $\mathcal{D} = \{(\mathbf{x}_i, y_i)\}_{i=1}^{m}$ in an efficient way the experiments were organized in a nested crossvalidation [?]. Here, crossvalidation (CV) is used for two different purposes (Fig S3):

1. Outer CV (on $\mathcal{D}$): Training and test sets ($\mathcal{D}_{tr}$ and $\mathcal{D}_{te}$) for classifier evaluation.

2. Inner CV (on $\mathcal{D}_{tr}$): Internal parameter selection for classifier training.

A crossvalidation ($r \times f$ CV) is a standard resampling scheme generating independent training and test set ($\mathcal{D}_{tr}$ and $\mathcal{D}_{te}$) systematically. It depends on the number of runs r and the number of folds f. The overall dataset is split into f folds of approximatelly equal size and class distribution $\mathcal{D} = \bigcup_{i=1}^{f} \mathcal{F}_i$. One of these folds is used as independent test set $\mathcal{D}_{te} = \mathcal{F}_i$. The remaining ones are used as a training set $\mathcal{D}_{tr} = \mathcal{D} \setminus \mathcal{F}_i$ for the classification model. The procedure is repeated for each individual fold leading to f independent evaluations. It is also repeated for r independent permutations of the sample set $\mathcal{D}$ in order to avoid sampling effects. Overall

a set of $r \times f$ evaluations is created and reported. For our evaluation, we have chosen the values $r = 5$ and $f = 10$.

Classification models

As classification models the random forest (RF) [?], the (linear) Support Vector Machine (SVM) [?], and the k-Nearest Neighbor Classifier (k-NN) [?] were chosen. The allowed parameter ranges are given in Table S3. All experiments were conducted in R with the TunePareto package [?].

Importance ranking

In contrast to SVM and k-NN, RFs are feature selecting classifiers that can additionally provide an importance score for each of the 102 measurements of the overall CAF profile. The random forest classifier is an ensemble classifier that aggregates the predictions of multiple decision trees (DT) for the classes (here: progress within 100 days vs. no-progress within 100 days) via a majority vote. It also aggregates the DT's internal importance scores for each cytokine via averaging. This internal structure can be used for calculating an importance score for characterizing the individual CAFs. It is calculated as the total decrease in node impurities from splitting on the cytokine (Gini-Index). For our analysis, we calculated the importance score for each model of the 5×10 CV. For each of the 5×10 experiments the importance scores were ranked. For the overall 5×10 CV the CAFs were sorted according to their median rank.

References

1. Hanahan D, Weinberg RA. Hallmarks of Cancer: The Next Generation. Cell. 2011;144(5):646–674. doi:10.1016/j.cell.2011.02.013.

2. Baraniskin A, Buchberger B, Pox C, Graeven U, Holch JW, Schmiegel W, et al. Efficacy of bevacizumab in first-line treatment of metastatic colorectal cancer: A sys-

tematic review and meta-analysis. European Journal of Cancer. 2019;106:37–44. doi:10.1016/j.ejca.2018.10.009.

3. Hurwitz H, Fehrenbacher L, Novotny W, Cartwright T, Hainsworth J, Heim W, et al. Bevacizumab plus Irinotecan, Fluorouracil, and Leucovorin for Metastatic Colorectal Cancer. New England Journal of Medicine. 2004;350(23):2335–2342. doi:10.1056/NEJMoa032691.

4. Van Cutsem E, Cervantes A, Nordlinger B, Arnold D. Metastatic colorectal cancer: ESMO Clinical Practice Guidelines for diagnosis, treatment and follow-up. Annals of Oncology. 2014;25:iii1–iii9. doi:10.1093/annonc/mdu260.

5. Chiorean EG, Nandakumar G, Fadelu T, Temin S, Alarcon-Rozas AE, Bejarano S, et al. Treatment of Patients With Late-Stage Colorectal Cancer: ASCO Resource-Stratified Guideline. JCO Global Oncology. 2020;6:414–438. doi:10.1200/JGO.19.00367.
