# Supplementary material for: Prediction of resistance to bevacizumab plus FOLFOX in metastatic colorectal cancer—Results of the prospective multicenter PERMAD trial
Source: PLoS One. 2024 Jun 14;19(6):e0304324. doi: 10.1371/journal.pone.0304324 (PMC11178165; doi:10.1371/journal.pone.0304324)
Supplement: S2 Text — (PDF) [file pone.0304324.s010.pdf]

S2 Text. Performance of the classifier discussion

As a linear classifier, a linear support vector machine constructs a linear combinations of CAFs as a unique decision criterion which is applied for every patient [*1*]. Consequently, the single connected decision region cannot take personal or temporal differences into account. A slightly better performance was observed for the k-nearest neighbor classifiers which construct their decision regions according to a prototype-based strategy. In contrast to linear classifiers, the prototype-based ones provide multiple disconnected decision regions for each class allowing to take into various disruptions. However, they are coupled to distance-based decision criteria, which typically leads to a uniform evaluation of biomarkers [*2*]. As a consequence individual predictive biomarkers might be covered a vast majority of non-informative ones. Finally the random forests [*3*], which can be seen as tree-based architectures, allow for both disconnected decision regions and an individual nonlinear analysis of each CAF. As a consequence a random forest is able to select predictive marker combinations that can be evaluated by various shifted copies of the same decision criterion.

References

1. Cortes C, Vapnik V. Support-vector networks. Machine Learning. 1995;20(3):273–297.
2. Fix E, Hodges JL. Discriminatory Analysis: Nonparametric Discrimination: Consistency Properties. USAF School of Aviation Medicine, Randolf Field, Texas; 1951. Project 21-49-004, Report Number 4.
3. Breiman L. Random forests. Machine Learning. 2001;45(1):5–32.
