# Supplementary material for: Prediction of resistance to bevacizumab plus FOLFOX in metastatic colorectal cancer—Results of the prospective multicenter PERMAD trial
Source: PLoS One. 2024 Jun 14;19(6):e0304324. doi: 10.1371/journal.pone.0304324 (PMC11178165; doi:10.1371/journal.pone.0304324)
Supplement: S1 Protocol — (PDF) [file pone.0304324.s015.pdf]

Investigational Plan & Clinical Study Protocol

PERMAD-Trial

Personalized marker-driven early switch to aflibercept in patients with metastatic colorectal cancer

A run in marker determination phase

followed by a marker-driven randomized part

- a multicenter, multinational, two-part, phase II trial.

Sponsor: University Hospital of Ulm

EudraCT Nr: 2012-005657-24

Protocol identification number: PERMAD01

Version: final v4 15-05-2014

Contact

Principal Investigator:

Thomas Seufferlein, University of Ulm, thomas.seufferlein@uniklinik-ulm.de

Steering Committee:

Dirk Arnold, Tumor Biology Center, Freiburg, arnold@tumorbio.uni-freiburg.de
Thomas Ettrich, University of Ulm thomas.ettrich@uniklinik-ulm.de
Armin Gerger, University of Graz armin.gerger@gmx.at
Stefan Kasper, University of Essen Kasper.Stefan@web.de
Nick Pavlakis, University of Sydney, nick.pavlakis@sydney.edu.au
Gerald Prager, General Hospital Vienna, gerald.prager@meduniwien.ac.at
Anke Reinacher-Schick, University St. Josef-Hospital Bochum Anke.Reinacher@rub.de
Holger Rumpold, BHS Linz Holger.Rumpold@bhs.at
Werner.Scheithauer, General Hospital Vienna, werner.scheithauer@meduniwien.ac.at
Eva Segelov, University of New South Wales, Sydney, e.segelov@unsw.edu.au
Thomas Seufferlein, University of Ulm thomas.seufferlein@uniklinik-ulm.de
Jeremy Shapiro, Monash University, Victoria, jeremy.shapiro@monash.edu
Alexander Stein, University Medical Center Hamburg-Eppendorf a.stein@uke.de

Study coordinator:

Alexander Stein, University Medical Center Hamburg-Eppendorf a.stein@uke.de

Legal sponsor:

University Hospital of Ulm

Biostatistics:

Hans Armin Kestler, University of Ulm (CAF part)
Anton Klingler, Assign DMB GmbH (Clinical part) anton.klingler@assigngroup.com

Data management, monitoring:

Assign DMB GmbH anton.klingler@assigngroup.com

Assign International GmbH immo.reinhardt@assigngroup.com

Laboratories:

Universitätsklinikum Ulm, Klinik für Innere Medizin I thomas.ettrich@uniklinik-ulm.de

Universitätsklinik für Innere Medizin I gerald.prager@meduniwien.ac.at
Medizininische Universität Wien
Comprehensive Cancer Center Vienna

Myriad RBM, Inc., Austin, Texas, USA www.myriadrbm.com

Approval of the Protocol

Signature

Date (DD Month YYYY)

Name of the Representative of the Sponsor

Signature

Date (DD Month YYYY)

Name of Coordinating Investigator

Coordinating Investigator's Institution

Signature

Date (DD Month YYYY)

Name of Statistician

Investigator's Agreement

I have read the attached protocol entitled *"Personalized marker-driven early switch to aflibercept in patients with metastatic colorectal cancer"* Version 4 dated 15.05.2014 and agree to abide by all provisions set forth therein.

I agree to comply with the International Conference on Harmonisation Tripartite Guideline on Good Clinical Practice.

I agree to ensure that the confidential information contained in this document will not be used for any purpose other than the evaluation or conduct of the clinical investigation without the prior written consent of the study sponsor.

Signature

Principal Investigator

Date (DD Month YYYY)

Investigator's Institution

Study Glossary

Abbreviation/Acronym	Definition
5-FU	5-Fluorouracil
ADCC	Antibody dependent cellular cytotoxicity
AE	Adverse event
ANC	Absolute neutrophil count
ANG-2	Angiopoietin 2
ALT (SGPT)	alanine aminotransferase (serum glutamic-pyruvic transaminase)
ASS	acetylsalicyclic acid
AST (SGOT)	Aspartate aminotransferase (serum glutamic-oxaloacetic transaminase)
bFGF	basic fibroblast growth factor
BSA	body surface area
BRAF	Serine/threonine-protein kinase B-Raf
BSC	best supportive care
BUN	blood urea nitrogen
BW	Body weight
CAF	cytokines and angiogenic factors
CAIX	carbonic anhydrase isozyme IX
CBC	complete blood count
CDC	Complement-dependent cytotoxicity
CEA	carcinoembryonic antigen
CHF	Congestive Heart Failure
CHO	Chinese hamster ovaries
CI	confidence interval
CLM	colorectal liver metastasis
CNS	central nervous system
CR	Complete response
CRC	colorectal cancer
CrCl	creatinine clearance
CRF	Case Report Form
CT	computerized tomography
CTCAE	Common Terminology Criteria for Adverse Events
CTx	Chemotherapy
CVA	Cerebrovascular accident
CXR	chest x-ray
DCR	Disease control rate

DPD	Dihydropypimidine Dehydrogenase
ECG	electrocardiogram
ECOG	Eastern Cooperative Oncology Group
FDA	Food and Drug Administration (U.S. government agency)
EGFR	Epidermal growth factor receptor
GCP	Good Clinical Practice
GCP-V	Verordnung über die Anwendung der Guten Klinischen Praxis bei der Durchführung von klinischen Prüfungen mit Arzneimitteln zur Anwendung am Menschen
GCSF	Granulocyte colony-stimulating factor
HGF	Hepatocyte growth factor
HIF1α	hypoxia-inducible factor-1α
IEC	Independent ethics committee
IDMC	Independent Data Monitoring Committee
IMP	investigational medicinal product
INR	International Normalized Ratio
ITT	Intention-to-treat
IV	intravenous
KRAS	Kirsten rat sarcoma
LDH	lactate dehydrogenase
LV	Leucovorin
mCRC	metastatic colorectal cancer
MMP-9	Matrix Metallopeptidase 9
moAB	Monoclonal antibody
MRI	Magnetic resonance imaging
NCI	National Cancer Institute
NRAS	Neuroblastoma RAS viral oncogene homolog
NSAID	Non-steroidal anti-inflammatory drug
NSCLC	Non Small Cell Lung Cancer
NYHA	New York Heart Association
ORR	Objective response rate
OS	overall survival
PD	progressive disease
PFS	progression free survival
PlGF	placenta like growth factor
PR	partial response
PTT	Partial Thromboplastin Time
QoL	Quality of life
RBC	red blood cell
RCT	radiochemotherapy

RDE	Remote data entry
RECIST	Response Evaluation Criteria in Solid Tumors
RR	response rate
RT	radiotherapy
SADR	serious adverse drug reaction
SAE	serious adverse event
SAS	statistic software
SD	stable disease
SLD	sum of the longest diameters
SmPC	Summary of Product Characteristics
sVEGFR2	soluble VEGF receptor 2
TTP	time to progression
ULN	upper limit of normal
UICC	Union internationale contre le cancer
VEGF	Vascular endothelial growth factor
WBC	white blood cells

Table of Contents

1. INTRODUCTION AND BACKGROUND 21
1.1 FIRST-LINE THERAPY IN PREVIOUSLY UNTREATED METASTATIC COLORECTAL CANCER 21
1.2 BEVACIZUMAB BASED FIRST LINE THERAPY 21
1.3 AFLIBERCEPT IN METASTATIC COLORECTAL CANCER 23
1.3.1 Aflibercept background 23
1.3.2 Preclinical data 25
1.3.3 Pharmacokinetics 28
1.3.4 Pharmacodynamics 28
1.3.5 Summary of clinical data 29
1.3.5.1 Phase I 29
1.3.5.2 Phase II 29
1.3.5.3 Phase III 30
1.3.6 Safety 30
1.3.6.1 Single agent studies 30
1.3.6.2 Combination studies 31
1.4 CYTOKINES AND ANGIOGENIC FACTORS (CAF) IN METASTATIC COLORECTAL CANCER 34
1.5 RATIONALE FOR THE EARLY SWITCH REGIMEN 34
1.6 RATIONALE FOR THE ANALYSIS OF CYTOKINES AND ANGIOGENIC FACTORS AND OTHER EVALUATIONS 36
1.7 RISK-BENEFIT ASSESSMENT 37
2. STUDY OBJECTIVE 37
3. STUDY DESIGN 38
3.1 PRIMARY ENDPOINT 38
3.2 SECONDARY ENDPOINTS 38
4. STUDY POPULATION 39
4.1 NUMBER OF PATIENTS 39
4.2 SELECTION CRITERIA 39
4.2.1 Inclusion criteria 39
4.2.2 Exclusion criteria 39
5. STUDY PROCEDURES AND METHODOLOGY 40
5.1 STUDY SCHEDULE OVERVIEW 40
5.1.1 Treatment 42
5.1.1.1 Treatment schedules 42
5.1.2 Treatment duration 42
5.1.3 Randomization 43
5.1.3.1 Arm A (conventional switch of chemotherapy together with the anti-angiogenic treatment) 43
5.1.3.2 Arm B (early marker-driven switch of anti-angiogenic agent and maintenance of chemotherapy) 43
5.1.4 Definite change of treatment (second line treatment) 43
5.1.5 Concomitant medication 43
5.2 ASSESSMENTS AND GUIDELINES FOR VISITS 45
5.2.1 Baseline Assessment 46
5.2.2 Assessments during treatment period 46
5.2.3 Randomization and/or change of treatment 47
5.2.4 End of Treatment 47
5.2.5 Follow-up Period 48
5.3 STUDY DURATION 48
5.4 RULES FOR CONTRACEPTION AND PREGNANCY REPORTING 48
5.5 STUDY TERMINATION 49
5.5.1 Patient Withdrawal 49
5.5.2 Study Completion 49
6. INVESTIGATIONAL MEDICAL PRODUCT: AFLIBERCEPT 49

6.1 STRUCTURE, MOLECULAR MASS 50
6.2 PREPARATION AND ADMINISTRATION OF AFLIBERCEPT 51
6.2.1 Preparation 51
6.2.2 Infusion conditions 52
6.2.3 Storage period of premix and infusion solution 52
6.2.4 Storage conditions and shelf life, destruction of used IMP 53

7. DOSE MODIFICATIONS 53

7.1 BEVACIZUMAB 53
7.1.1 Surgical procedures / wound healing complications 54
7.1.2 Hypertension 54
7.1.3 Proteinuria 54
7.1.4 First occurrence of proteinuria during treatment with bevacizumab 55
7.1.5 Thrombosis / embolism 56
7.1.6 Haemorrhage 56
7.1.7 Congestive Heart Failure 56
7.1.8 Tracheo-oesophageal fistula 57
7.1.9 Hypersensitivity reactions/infusion reactions 57
7.2 AFLIBERCEPT 57
7.2.1 Hypertension therapy recommendations: 59
7.2.2 Proteinuria 59
7.2.3 Reversible posterior leuko-encephalopathy (RPLS) or clinical symptoms related to vasogenic edema of the white matter. 60
7.2.4 Gastro-intestinal perforation: 60
7.2.5 Hypersensitivity reaction: 60
7.2.6 Wound healing complications/surgery 61
7.3 DOSE MODIFICATIONS FOR CHEMOTHERAPY 61
7.3.1 General notes regarding dose modifications for chemotherapy-related toxicity 61
7.3.2 Guidelines for dose modifications 62
7.3.3 Toxicity at the start of the following cycle 62
7.3.4 Toxicity and specific dose modification: oxaliplatin 64
7.3.5 Toxicity of irinotecan and guidelines for treatment of diarrhea 64

8. CRITERIA OF EVALUATION 66

8.1 PROGRESSION FREE SURVIVAL 66
8.1.1 PFS of first line treatment (PFS 1) 66
8.1.2 PFS after first cycle after randomization (PFS r) 66
8.1.3 PFS of second line treatment 66
8.2 TIME TO RANDOMIZATION 66
8.3 RESPONSE RATE 66
8.3.1 RECIST Criteria 66
8.4 SECONDARY RESECTION RATE 68
8.5 OVERALL SURVIVAL 68
8.6 SAFETY ENDPOINTS 68
8.7 QUALITY OF LIFE (QOL) 68
8.8 FURTHER EVALUATIONS 68

9. ANALYSES OF CYTOKINES AND ANGIOGENIC FACTORS 68

9.1 TECHNICAL ASPECTS 68

10. ASSESSMENT OF ADVERSE EVENTS 69

10.1 DEFINITIONS 69
10.2 REPORTING PROCEDURE FOR ALL ADVERSE EVENTS 70
10.2.1 Assessment of Causality of Adverse Events 71
10.2.2 Assessment of Severity of Adverse Events 71
10.3 SERIOUS ADVERSE EVENTS REPORTING 72

11. DATA ANALYSIS AND STATISTICAL CONSIDERATIONS 72
11.1 RUN IN PHASE FOR DETERMINATION OF CAF PROFILE BASED ON PLGF AND VEGF-B FOR EARLY DETECTION OF PROGRESSION . 72
11.1.1 Prognostic value of baseline values 72
11.1.2 Determination of the association of repeated measurement of CAF levels particularly PlGF and VEGF-B on PFS/response status 73
11.2 INDEPENDENT DATA MONITORING COMMITTEE (IDMC) 73
11.3 RANDOMIZED PHASE - SAMPLE SIZE CALCULATION 73
11.4 POPULATIONS FOR ANALYSIS 74
11.5 PATIENT DEMOGRAPHICS/OTHER BASELINE CHARACTERISTICS 74
11.6 TREATMENTS (STUDY TREATMENTS) 74
11.7 EFFICACY ANALYSIS 74
11.7.1 Primary Efficacy Endpoint 74
11.7.2 Secondary Efficacy Endpoints 75
12. DATA MANAGEMENT 75
12.1 RANDOMIZATION PROCEDURE 75
12.2 DATA CAPTURE 76
13. QUALITY ASSURANCE 76
13.1 STANDARDIZATION 76
13.2 DATA ACCESS 76
13.3 MONITORING/ SOURCE DATA VERIFICATION (SDV) 76
13.4 AUDITS AND INSPECTIONS 76
14. REGULATORY AND LEGAL OBLIGATIONS 77
14.1 GENERAL PROVISIONS/DECLARATION OF HELSINKI 77
14.2 PATIENT PROTECTION 77
14.3 COMPETENT AUTHORITY 77
14.4 INDEPENDENT ETHICS COMMITTEE 77
14.5 AMENDMENTS 78
14.6 STUDY REPORTS 78
14.7 INFORMED CONSENT 78
14.8 SUBJECT CONFIDENTIALITY 79
14.9 STUDY DOCUMENTATION AND ARCHIVE 79
14.10 COMPENSATION 80
15. TRIAL SPONSORSHIP AND FINANCING 80
16. TRIAL INSURANCE 80
17. PUBLICATION POLICY 81

Table of appendices

Appendix A: Bibliography
Appendix B: ECOG Performance Status Scale
Appendix C: Common Terminology Criteria for Adverse Events v4.0
Appendix D: RECIST v1.1
Appendix E: TNM Clinical Classification
Appendix F: EORTC QLQ-C30 and the module CR29

List of Tables and Figures

Tables:

1. In vivo antitumor activity of aflibercept in combination with chemotherapy
2. Most frequent AEs in the VELOUR phase III trial (excluding anti VEGF class effects)
3. Anti-VEGF associated events in the VELOUR trial (overall population)
4. Anti-VEGF associated events by prior use of bevacizumab in patients receiving aflibercept
5. Assessments overview
6. Management of Hypertension
7. Aflibercept dose reduction level
8. Dose modifications for aflibercept
9. Acute infusion reaction management
10. Dose modification for chemotherapy-induced toxicity
11. Dose modification for oxaliplatin-induced neuropathy
12. Response evaluation according to RECIST

Figures:

1. Aflibercept schematic representation (VEGF Trap)
2. Patterns of PlGF levels during bevacizumab and chemotherapy
3. Patterns of bFGF, HGF and MMP-9 levels during bevacizumab and chemotherapy
4. Study schedule overview (run in phase with conventional switch of chemotherapy together with the anti-angiogenic agent)
5. Study schedule overview (randomized part with marker-driven switch of anti-angiogenic agent and maintenance of chemotherapy)
6. Overview time-points and patient accrual
7. Aflibercept/$VEGF_{R1R2}$ structure
8. Algorithm of Bevacizumab Dose Interruption in Case of Dipstick 2+ Proteinuria
9. Algorithm of Bevacizumab Dose Interruption in Case of Dipstick ≥ 3+ Proteinuria

Synopsis

**Title**	**Personalized marker-driven early switch to aflibercept in patients with metastatic colorectal cancer (PERMAD-Trial) - a multicenter, multinational, two-part, phase II trial**
**Design**	Multicenter, multinational two part, phase II trial with a run-in marker determination phase followed by a marker-driven randomized part
**EudraCT No**	2012-005657-24
**Sponsor**	University of Ulm
**Indication**	Patients with metastatic or recurrent colorectal cancer
**Planned sample size**	Overall 200 patients to be enrolled<br>• 50 patients for run-in phase<br>• 150 patients for marker-driven part with 86 patients to be randomized
**Number of centers**	Approx. 28 sites in Germany, Austria and Australia
**Rationale**	Treatment of metastatic colorectal cancer (mCRC) is based on a variety of available patient-, tumor- and drug-related factors,e.g. co-morbidity, tumor related symptoms, potential resectability. Despite the proven negative predictive value of KRAS mutation for EGFR antibody treatment, no marker is currently available to guide upfront treatment decision, especially for antiangiogenic treatment.<br>Recent research indicated the potentially predictive value of cytokines and angiogenic factors (CAF) for early detection of progression during treatment with chemotherapy and bevacizumab (e.g. PlGF, bFGF, HGF, MMP-9). The impact of these results on clinical management of patients is not yet determined, although early treatment modification based on a biomarker might be a meaningful approach. In regard of the available data changes in a single CAF does not seem to reliably predict disease progression. Thus, evaluation of a CAF profile enabling early prediction of progression (before radiologic progression) is of high interest and potential clinical value.<br>Currently, results of two large second line phase III trials (VELOUR and TML) have challenged angiogenic treatment strategies in mCRC. Both trials demonstrated the beneficial impact on survival for continuation of antiangiogenic treatment, with either aflibercept after bevacizumab or continuation of bevacizumab beyond progression with a changed chemotherapy backbone. Aflibercept targets VEGF isoforms A and B and PlGF and might thus have a stronger antiangiogenic effect compared to

	bevacizumab. The concept of maintaining VEGF inhibition and change chemotherapy in case of radiologic progression has been proven in the TML trial. However, early change of bevacizumab to the potentially more effective angiogenesis inhibitor aflibercept in case of forthcoming progression, albeit not yet radiologically visible, maintaining chemotherapy might be beneficial in terms of progression free survival and thus delay change to second line chemotherapy. The impact of this marker-driven treatment modification on efficacy and CAF is unknown and will therefore be evaluated in the current trial.
**Objectives**	Overall objective of the two phase PERMAD trial is the evaluation of the impact of a personalized marker-driven treatment approach with early detection of progression and modification of treatment on cytokines and angiogenic factors (CAF) and efficacy.<br>**Run-in phase with conventional switch of chemotherapy together with the anti-angiogenic agent**<br>Primary objective of the run-in phase is the determination of a distinct cytokines and angiogenic factor (CAF) profile based on PIGF and VEGF-b during treatment with FOLFOX and bevacizumab, which allows early detection/prediction of progressive disease.<br>**Randomized part with marker-driven switch of anti-angiogenic agent and maintenance of chemotherapy.**<br>Primary objective of the marker-driven randomized part is the evaluation of the efficacy of an early marker-driven switch of anti-angiogenic treatment (bevacizumab to aflibercept) maintaining the chemotherapy backbone until definite radiological progression compared to a conventional treatment approach of changing chemotherapy and antiangiogenic agent at time of radiologic progression.<br>Secondary objectives of both parts are prognostic and predictive value of CAF at baseline and the changes during treatment, efficacy, tolerability and patient related outcome.
**Endpoints**	**Run-in phase with conventional switch of chemotherapy together with the anti-angiogenic agent.**<br>Primary endpoint:<br>• Progression free survival (PFS1) of first line treatment<br>**Randomized part with marker-driven switch of anti-angiogenic agent and maintenance of chemotherapy.**<br>Primary endpoint:<br>• PFS rate at 6 months (PFSR@6) after first cycle after randomization<br>Secondary endpoints:

	• Predictive value of CAF particularly PIGF and VEGF-B for early detection of progression during treatment with chemotherapy and bevacizumab<br>• Determination and validation of a CAF profile based on PIGF and VEGF-B predicting tumor progression before radiologic progression<br>• PFS1, after first cycle after randomization (PFSr) and of second line treatment (PFS2)<br>• Time to randomization (TTR)<br>• Overall survival (OS)<br>• Overall response rate (RR) and Secondary resection rate (sRR)<br>• Toxicity, Quality of life (QoL)<br>• Changes in CAF during early marker-driven switch and conventional treatment approach<br>• Prognostic value of CAF at baseline and/or during treatment

Overview run-in phase with conventional switch of chemotherapy together with the anti-angiogenic agent

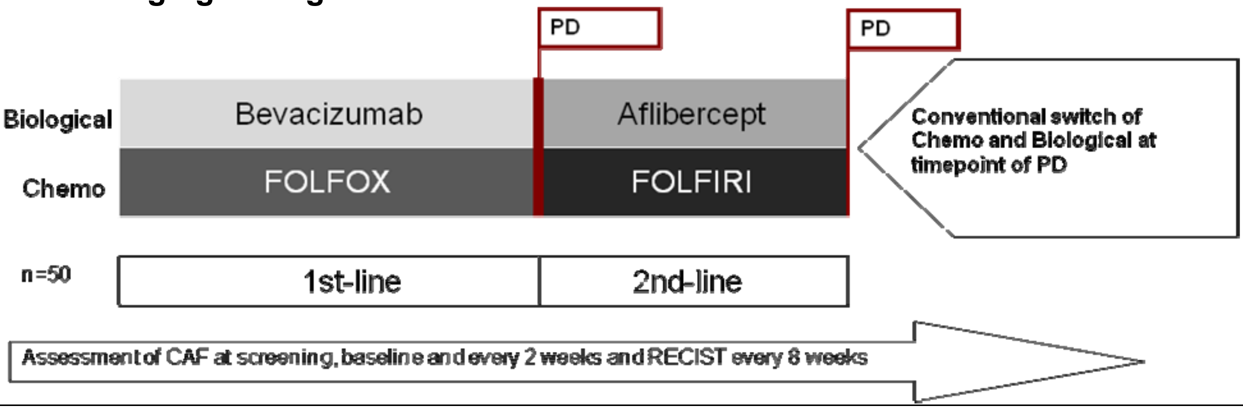

After completing the run in phase of the study, with at least 30 patients completing their first line treatment (progression, secondary resection, toxicity) and being evaluable for CAF analyses, the results will be reviewed by an Independent Data Monitoring Committee (IDMC). Based on that review the decision to continue with, modify or cancel the randomized part will be made. If no CAF profile predictive for radiographic progression can be detected, the randomized study part will not be initiated (see 5.1).

Overview randomized part with marker-driven switch of anti-angiogenic agent and maintenance of chemotherapy.

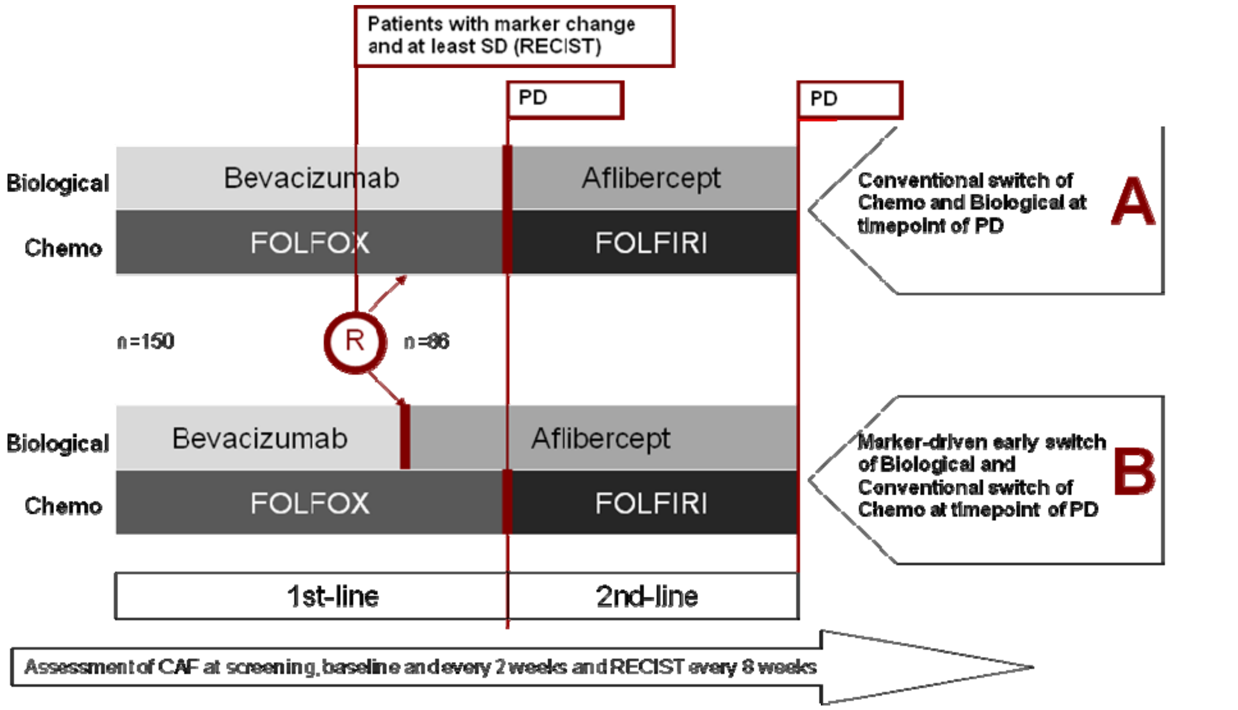

**Main selection criteria**	▪ Patients with histologically confirmed diagnosis of unresectable stage IV (UICC) colorectal cancer (primary tumor may be present)<br>▪ Patients with at least one measurable lesion, with size > 1 cm (RECIST v1.1)<br>▪ ECOG Performance status ≤ 2<br>▪ Life expectancy > 3 months<br>▪ Age ≥ 18 years.<br>▪ Haematologic function as follows: ANC ≥ 1.5 x 10^9/L, platelets ≥ 100 x10^9/L, hemoglobin ≥ 9 g/dl or 5.59 mmol/l<br>▪ In patients not receiving therapeutic anticoagulation INR <1.5 and aPTT < 1.5 x ULN within 7 days prior to starting study treatment.<br>▪ Adequate liver function as measured by serum transaminases (AST & ALT) ≤ 2.5 x ULN (in case of liver metastases < 5 x ULN) and total bilirubin ≤ 1.5 x ULN<br>▪ Adequate renal function: Serum creatinine ≤ 1.5 x ULN<br>▪ Signed, written informed consent<br>▪ At least 6 months after completion of adjuvant chemotherapy.
**Treatment, Dosage and Administration**	All enrolled patients will be treated 1st line with bevacizumab and mFOLFOX6 every two weeks<br>**Bevacizumab**

	bevacizumab 5 mg/kg iv over 30 to 90 min (day 1)<br>**mFOLFOX6**<br>oxaliplatin at a dose of 85 mg/m^2 iv over two hours (day 1)<br>5-FU 400 mg/m^2 iv bolus (day 1)<br>LV at a dose of 400 mg/m^2 iv over two hours (day 1)<br>5-FU at a dose of 2400 mg/m^2 iv over 46 hours (day 1-3)<br>**Treatment duration**<br>Treatment with bevacizumab and mFOLFOX6 will be administered until progression (according to RECIST v1.1), intolerable toxicity or secondary resection in the run in phase. In the marker driven part bevacizumab and mFOLFOX6 will be administered until change of the CAF-profile to be determined in the run in phase and at least stable disease according to RECIST v1.1, or progression, intolerable toxicity or secondary resection.<br>**Randomization**<br>All patients in the marker driven part with change of respective marker profile and at least stable disease according to RECIST v1.1 will be randomized to<br>**Arm A (conventional switch of chemotherapy together with the anti-angiogenic agent)**<br>Bevacizumab and mFOLFOX6 (continuation of same regimen until progressive disease (PD) according to RECIST v1.1, switch to aflibercept and FOLFIRI after PD)<br>**Arm B (early marker-driven switch of anti-angiogenic agent and maintenance of chemotherapy)**<br>Aflibercept and mFOLFOX6 (change of bevacizumab to aflibercept and continuation of mFOLFOX6 until PD according to RECIST v1.1, followed by change to FOLFIRI after PD)<br>All enrolled patients will be treated 2nd line with aflibercept and FOLFIRI every two weeks.<br>**Aflibercept**<br>aflibercept 4mg/kg iv over one hour (day 1)<br>**FOLFIRI**<br>irinotecan 180 mg/m^2 iv over one hour (day 1)<br>5-FU 400 mg/m^2 iv bolus (day 1)<br>LV at a dose of 400 mg/m^2 iv over two hours (day 1)<br>5-FU at a dose of 2400 mg/m^2 iv over 46 hours (day 1-3)<br>**Definite change of treatment (second line treatment)**<br>In case of radiological progression at any time point treatment with bevacizumab/aflibercept and mFOLFOX6 will be changed to aflibercept and FOLFIRI (change of chemo-backbone)

**IMP Definition**	Bevacizumab, 5FU, leucovorin, irinotecan and oxaliplatin are approved for the treatment of metastatic colorectal cancer. Aflibercept will be supplied and defined as IMP.
**Statistical Considerations (Randomized Part)**	The majority of patients continuing treatment with bevacizumab and mFOLFOX6 after change of CAF-profile compared baseline are expected to progress at the next scheduled staging (within two months). Thus, PFSR@6 months after first cycle after randomization will be 20% for arm A (conventional switch). The early marker-driven switch is expected to result in a PFSR@6 of at least 45%. The trial should achieve 80% power to detect differences between the treatment arms and keeping the type I error level below 10%.<br><br>The planned number of patients with a drop out rate of about 10% will be 43 patients per arm. Thus, 86 patients need to be randomized. About 60% of patients will be eligible for randomization (at least stable disease and distinct maker profile) so all together 150 patients are required for the randomized part.<br><br>The trial uses a randomized parallel arm phase II design.
**Assessments**	**Initial staging**<br>• Review of inclusion and exclusion criteria<br>• Medical and medication history, physical examination including height, weight, vital signs (blood pressure, heart rate, respiratory rate, body temperature), ECOG-performance status<br>• Laboratory Tests:<br>Hematology panel: hemoglobin, hematocrit, count, platelets, white blood cell (WBC) count and WBC differential (neutrophils, lymphocytes)<br>Chemistry panel: sodium, potassium, calcium, creatinine, total bilirubin, alkaline phosphatase, alanine aminotransferase (ALT), aspartate aminotransferase (AST), total protein, albumin, LDH<br>Coagulation: INR, aPTT, PT<br>CEA, (CA 19-9)<br>Urine dipstick<br>Pregnancy test for women of childbearing potential within 7 days prior to start of the treatment<br>• Blood draw (7.5 ml) for CAF analysis (screening, baseline and every 2 weeks (run-in phase) or 4 weeks (randomized part) afterwards)<br>• ECG

	• Radiological imaging of the chest, abdomen and all other sites of disease (CT/MRI-scan of the thoracic and abdominal region).<br>• Obtain paraffin-embedded tumor-tissue<br>• QoL will be assessed using the EORTC QLQ-C30 and the module CR29.<br>**Previous to any new cycle**<br>• Vital signs including weight, ECOG-performance status, assessment of toxicity, concomitant medication, physical Examination (as clinically indicated)<br>• Laboratory Tests: hemoglobin, platelets, white blood cell, neutrophils, sodium, potassium, calcium, creatinine, total bilirubin, alanine aminotransferase (ALT), aspartate aminotransferase (AST), urine dipstick<br>• Blood draw (7.5 ml) for CAF analysis (screening, baseline and every 2 weeks (run-in phase) or 4 weeks (randomized part) afterwards)<br>**Final staging**<br>When any subject discontinues dosing of all study treatment, the following assessments should be made.<br>• Physical examination, vital signs including weight, ECOG-performance status, assessment of toxicity, concomitant medication<br>• Laboratory Tests: hemoglobin, platelets, white blood cell, neutrophils, lymphocytes, sodium, potassium, calcium, creatinine, total bilirubin, alkaline phosphatase, alanine aminotransferase (ALT), aspartate aminotransferase (AST), total protein, urine dipstick<br>• Tumor response assessment<br>• ECG<br>• QoL will be assessed using the EORTC QLQ-C30 and the modules CR29.<br>**Follow-up**<br>All subjects will be contacted every 3 months ± 28 days up to a maximum of 5 years per individual patient after trial inclusion.<br><br>In case of progressive disease after second line treatment only:<br>• Survival, disease status, protracted toxicity, further treatment<br>In any other case additionally:<br>• Tumor assessment, physical examination including weight,

	ECOG-performance status<br>**Tumor Response Assessment**<br>During treatment tumor response will be assessed by the investigator according to RECIST v1.1 (CT and/or MRI) every 4 cycles (8 weeks) CT and/or MRI scans will be retrospectively independently reviewed.<br>**CAF**<br>Plasma PlGF, Ang 2, VEGFs, G-CSF, sVEGFR2, HGF, bFGF, MMP-9 will be evaluated at screening (day -28 to -7), baseline (d1 just before treatment) and every 2 weeks (run-in phase) or 4 weeks (randomized part) afterwards. Further markers gaining importance during the trial will be analyzed.<br>Venous blood draw of 7.5 ml will be performed at screening, baseline and every 4 weeks (every 2 weeks during run-in phase) during treatment, followed by immediate plasma preparation, division into five equal aliquots, storage by -80°C (or alternatively -20°C for up to 8 weeks) and shipment to central laboratory every two months during marker driven part or after enrolment of 60% of patients and at the end of the run in phase (baseline samples will be shipped together with first 8 week assessment). Marker analysis will be performed centrally as soon as possible and eligible patients (at least stable disease according to RECIST) will be randomized during the next treatment cycle (after 5th/9th/13th or 17th cycle). CAF assessment will be performed every 4 weeks until progression during second line treatment with aflibercept.<br>**Translational research on tumour tissue**<br>Paraffin embedded tissue (FFPE) will be evaluated for CAIX/HIF1a, Npn-1, VEGFs, Bv8, PDGF-C, VEGFRs, CEACAM5, vascular density, KRAS, NRAS and BRAF (baseline). Optional rebiopsy at time of randomization will be performed. Upon collection tissue will be divided in three parts one for FFPE, one in RNA later and one fresh frozen at -80°C. FFPE of rebiopsy will be evaluated for CAIX/HIF1a, Npn-1, VEGFs, Bv8, PDGF-C, CXCR-4, SDF-1 and VEGFRs. RNA later and fresh frozen tissue will be stored for further analyses.<br>**Safety**<br>Toxicity will be graded according to NCI CTCAE version 4.03. Physical examination including weight and vital signs and ECOG-performance status will be determined at every visit.<br>**Quality of life**<br>QoL will be assessed using the EORTC QLQ-C30 and the module CR29 at baseline, every 8 weeks and at the end of treatment.
**Study Duration**	Randomization will be performed median 8 months after enrollment and start of bevacizumab+chemotherapy. Median PFS

**(individual patient)**	with bevacizumab+ mFOLFOX6 will be about 10 months (Fuchs, Marshall et al. 2007; Saltz, Clarke et al. 2008; Kopetz, Hoff et al. 2010). Second line Chemotherapy and aflibercept demonstrated a median PFS of 6.9 months. Estimated median time on study will be about 17 months for both run-in phase and randomized part. Maximum time on trial including follow up will be 5 years per patient.
**Independent Data Monitoring Committee (IDMC)**	The results of the run-in phase (predictive value of CAF profile for radiographic progression) will be reviewed by the IDMC after available data on CAF and efficacy of the first 30 and again after the first 50 patients, to decide about conducting the randomized part.
**Timelines**	First patient in: QII 2014<br>Last patient in: QIV 2016<br>(recruitment 30 months)<br>Last patient out/study completion: QII 2018<br>Follow up for overall survival until: QII 2020

1. Introduction and Background

1.1 First-line therapy in previously untreated metastatic colorectal cancer

Colorectal cancer (CRC) is the most frequently diagnosed cancer in Europe and one of the leading causes of cancer death worldwide (Ferlay, Shin et al. 2010; Jemal, Bray et al. 2011). About 20-25% of patients with CRC present with metastatic disease at time of diagnosis, and further 20-25% of patients will develop metastases after curative resection.

During the past few years the treatment outcome of patients with metastatic colorectal cancer (mCRC) has improved considerably with the introduction of several new cytotoxic agents as capecitabine, irinotecan and oxaliplatin (Wolpin and Mayer 2008). Parenterally or orally administered fluoropyrimidines have been given with irinotecan, in regimens known as FOLFIRI (folinic acid, fluorouracil, and irinotecan) or CAPIRI/IRIS (capecitabine/S1 and irinotecan) and with oxaliplatin (FOLFOX/XELOX) (Douillard, Cunningham et al. 2000; Koopman, Antonini et al. 2007; Arkenau, Arnold et al. 2008; Kohne, De Greve et al. 2008; Muro, Boku et al. 2010). The use of these drug combinations in mCRC has significantly prolonged overall survival (OS). In particular, FOLFOX and FOLFIRI have significantly improved clinical efficacy as related to overall response rates, time to tumor progression, and lead to an improved overall survival. Both FOLFOX and FOLFIRI are appropriate, highly active first-line regimens that may also be administered as second-line salvage chemotherapy after progression of each first line (Tournigand, Andre et al. 2004).

Bevacizumab, cetuximab and panitumumab are monoclonal antibodies approved for treatment of mCRC. The addition of these targeted agents to the mCRC treatment armamentarium has provided increased therapeutic options and improved treatment outcomes for patients. Bevacizumab, cetuximab and panitumumab are available for first and further line treatments in combination with chemotherapy or as single agent (only EGFR antibodies) (Cunningham, Humblet et al. 2004; Hurwitz, Fehrenbacher et al. 2004; Giantonio, Catalano et al. 2007; Jonker, O'Callaghan et al. 2007; Van Cutsem, Peeters et al. 2007; Saltz, Clarke et al. 2008; Sobrero, Maurel et al. 2008; Peeters, Price et al. 2010; Bokemeyer, Bondarenko et al. 2011; Douillard 2011; Van Cutsem, Kohne et al. 2011). Efficacy of EGFR antibodies is limited to patients with KRAS wildtype (Amado, Wolf et al. 2008; Karapetis, Khambata-Ford et al. 2008).

Further drugs like aflibercept and regorafenib have recently reported promising results in combination with chemotherapy in second line or as single agent in refractory disease (Tabernero 2011; Van Cutsem 2011; Grothey 2012).

Today, the choice of first-line treatment in metastatic colorectal carcinoma should basically depend on the clinical situation at the time and on the goals of treatment, because independent positive predictive factors have not yet been established.

1.2 Bevacizumab based first line therapy

Several trials evaluated the efficacy of bevacizumab containing first-line regimens. In the first phase III study (study AVF2107), performed in 815 previously untreated patients with mCRC, the efficacy of the IFL regimen (irinotecan 125 mg/m2 90-minute infusion, followed by LV 20 mg/m2 IV bolus, followed by 5-FU 500 mg/m2 IV bolus; once weekly for 4 weeks, repeated every 6 weeks) was compared with that of IFL plus bevacizumab (5

mg/kg IV every 2 weeks) (Hurwitz, Fehrenbacher et al. 2004).

The addition of bevacizumab significantly prolonged OS and PFS, while the response rate was notably higher (45% vs. 35% for IFL + placebo; p=0.0029) and duration of response significantly longer (10.4 months vs. 7.1 months for IFL + placebo; p=0.0014) in patients in the bevacizumab group. Grade 3 hypertension was reported in 11% of patients in the bevacizumab group and 2% of those in the IFL group (p<0.01) but was easily manageable. Thrombotic events occurred in 19% of patients in the bevacizumab plus IFL group and 16% of those in the IFL group. Based on these findings, the US Food and Drug Administration (FDA) approved the combination of 5-FU-based chemotherapy plus bevacizumab for first-line treatment of patients with mCRC in 2004.

The NO16966 study was initially designed to prove non-inferiority of the XELOX regimen compared with FOLFOX-4. After the presentation of the significant effect of the addition of bevacizumab to IFL in 2003 this trial was amended to assess the potential improvement of the addition of bevacizumab to XELOX or FOLFOX-4 in a 2x2 factorial design (Hurwitz, Fehrenbacher et al. 2004). Response rates in this trial were not different between the bevacizumab and placebo arms. However, the rate of potentially curative surgery occurred in 55 (9.6%) patients on bevacizumab vs. 38 (6.6%) on placebo (p=0.061) in the per protocol population. Curative hepatic resections occurred in 48 (8.4%) cases on bevacizumab vs. 32 (5.6%) on placebo (p=0.059). No increase in SAE, death, or treatment-related death was observed in patients treated with bevacizumab undergoing surgery. Median progression-free survival (PFS) was significantly better in the bevacizumab group, 9.4 vs. 8.0 months in the placebo group (hazard ratio 0.83; 97.5% CI, 0.72 to 0.95; p=0.0023). However, median overall survival was 21.3 months in the bevacizumab group and 19.9 months in the placebo group (HR, 0.89; 97.5% CI, 0.76 to 1.03; p=0.077) (Saltz, Clarke et al. 2008).

The observational phase IV trial MO18024 (First BEAT) started accrual in June 2004. Nearly 2000 patients with non-resectable mCRC were recruited in 41 countries and started first-line chemotherapy (according to the treating physician’s choice) in combination with bevacizumab (5 mg/kg every 2 weeks in combination with 5-FU-based chemotherapy or 7.5 mg/kg every 3 weeks in combination with capecitabine-based chemotherapy) given until disease progression. The aim of the study was to gain safety experience in a relatively unselected patient population resembling usual critical practice. The results of this large community-based study confirmed the safety profile of bevacizumab in first-line metastatic CRC patients receiving a variety of chemotherapy regimens, namely FOLFOX, XELOX, FOLFIRI, or capecitabine. Overall, data from First BEAT study showed that resections with curative intent were performed in 11.8% (225/1914) with 76.9% (173/225) R0 resections in patients treated with bevacizumab and first-line chemotherapy for mCRC (Cassidy 2008; Okines, Puerto et al. 2009). Best results were obtained in patients with metastatic disease limited to the liver. Achieving a resection with curative intent has a clear impact on PFS and most likely on OS. Oxaliplatin-based regimens, particularly FOLFOX, appear to be the most effective treatment choice, even though a selection bias cannot be excluded as the study design allowed the physician to choose the treatment (Van Cutsem, Rivera et al. 2009). The observational phase IV trial (BriTE) displayed similar results in nearly 2000 patients in the US, revealing the expected and manageable toxicities (Grothey, Sugrue et al. 2008).

1.3 Aflibercept in metastatic colorectal cancer

1.3.1 Aflibercept background

Further details on preclinical, clinical safety and preliminary efficacy are provided in the Investigator's Brochure, which contains comprehensive information on aflibercept.

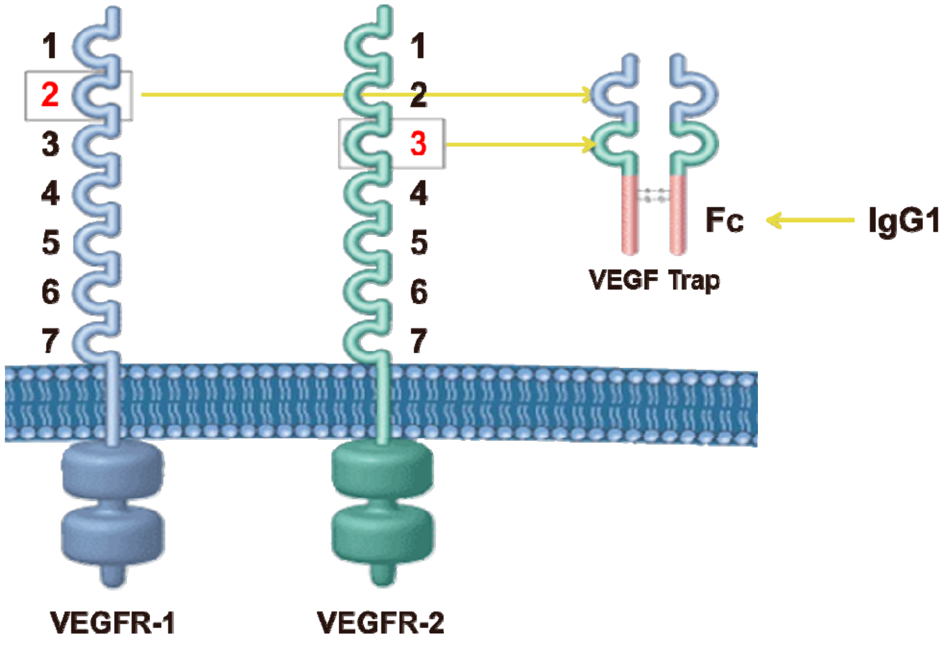

Figure 1 – Aflibercept schematic representation (VEGF Trap)

Aflibercept is a recombinant fusion protein consisting of human vascular endothelial growth factor (VEGF) receptor extracellular domains fused to the Fc portion of human immunoglobulin G1 (IgG1). [1] Aflibercept contains portions of the extracellular domains of 2 different vascular endothelial growth factor receptors (VEGFRs): VEGFR1 (also known as Flt-1) and VEGFR2 (also known as KDR or Flk-1). Aflibercept drug product is formulated as a sterile liquid for intravenous (IV) administration. Aflibercept binds VEGF in the picomolar (pmol/L) range, and also binds placental growth factor (PlGF), although with lower affinity. The affinity constants (KD) for binding to 2 human isoforms of VEGF, VEGF165 and VEGF121, are 0.50 pmol/L and 0.36 pmol/L, respectively. The KD for human PlGF2 is 39 pmol/L. The binding of aflibercept to its ligands in vivo is expected to block tumor angiogenesis and vascular permeability. Aflibercept has been found to be active with a broad pharmacological index against early and advanced stage disease in a variety of preclinical solid tumor models including sarcomas, and ovarian, prostate, mammary, colon, and gastric carcinomas when used as a single agent or in combination with cytotoxic agents. In mouse models of ascites formation with ovarian and renal cell carcinoma, aflibercept inhibited ascites formation and reduced tumor burden.

Two analyses were assayed in animal models specifically by enzyme linked immunosorbent assay (ELISA) methods: free aflibercept (compound not complexed to VEGF), and bound aflibercept (complexed aflibercept: VEGF [ratio 1/1]).

Following IV administration in all animal species evaluated, free aflibercept was characterized by a low clearance (0.5 to 3 mL/hr/kg), a low volume of distribution (69 to 226 mL/kg), and a long apparent elimination half-life (t1/2) of 48 to 98 hours. Based on

the correlation between exposure and activity in non-clinical models, the target pharmacological exposure in humans is proposed to be a safely administered dose of aflibercept at which an excess of free aflibercept is sustained.

The toxicity profile of aflibercept was evaluated in monkeys. The main compound-related microscopic findings were in the bone, nasal cavity, kidney, ovary, and adrenal gland. In the bone, aflibercept-induced effects consisted mainly of thickening of the growth plate and osteocartilaginous exostoses observed on the axial and appendicular skeleton that correlated with hunched posture at clinical examination. In the nasal cavities, degeneration/regeneration of the respiratory and olfactory epithelium, and atrophy/loss of nasal septum and/or turbinates was often associated with hemorrhage and suppurative exudate. Histopathologic findings in the kidneys (increased glomerular mesangial matrix) were associated in a few animals with decreased serum total protein and albumin levels and increased serum blood urea nitrogen (BUN) and urine protein and/or microalbumin levels. In the ovaries, the decreased number of maturing follicles, granulosa cells, and/or theca cells was associated with an overall inhibition of the female reproductive function. In the adrenals, a decreased vacuolation of adrenal zona fasciculata cells with cytoplasmic eosinophilia was observed. In addition, focal vascular proliferation/degeneration was noted in a range of organs, including in particular the digestive system, urinary bladder, heart, and brain of a few monkeys. In addition, increased liver enzyme levels were noted in a few monkeys with portal inflammation and necrosis. Aflibercept administration also resulted in a decrease in sperm motility and increased incidence of abnormal spermatozoa morphology. Most aflibercept-related findings were noted from the lowest doses tested (1.5 to 3 mg/kg/administration). With the exception of skeletal and nasal findings, aflibercept-related findings were reversible within 5 months after treatment cessation. In sexually immature monkeys treated for 3 months, the main compound-related findings were comparable to those in sexually mature monkeys. SC administration of aflibercept to male mice resulted in significant reductions in microvessel density mainly within the liver, pancreatic islets and thyroid follicles at all doses and to a lesser extent in the pituitary glands and adipose tissue. The single SC administration of aflibercept to rodents resulted in a moderate, long lasting but reversible increase in systolic and diastolic blood pressure. The blood pressure in rats treated with aflibercept was lowered by several classes of anti-hypertensive agents: e.g. angiotensin converting enzyme inhibitors, calcium channel blockers, an adrenergic receptor antagonist and modulator of nitric oxide availability. Aflibercept was shown to induce a moderate delay in wound repair and healing from 0.3 mg/kg/administration after IV administration in rabbits.

When administered IV to pregnant rabbits (as a 30 minute infusion once daily, on gestation Days 6, 9, 12, 15, and 18 - a total of 5 administrations) aflibercept induced minimal to moderate maternal toxicity, abortion, and embryolethality at 60 mg/kg/administration. External, visceral, and/or skeletal malformations were observed in fetus from pregnant rabbits treated from 3 mg/kg/administration (approximately 1.3 times the exposure in patients treated at the recommended human dose). Aflibercept has been administered to more than 3000 patients with advanced solid malignancies in clinical oncology trials, to 76 healthy subjects, and to 41 ophthalmology patients. Doses have been administered up to 800 µg/kg subcutaneously (SC) twice weekly, 7 mg/kg IV every 2 weeks, and 9 mg/kg IV every 3 weeks.

Single agent Phase I studies are completed and several Phase I studies as combination chemotherapy are ongoing. To date, 6 Phase II studies of aflibercept as single agent or in combination with standard cytotoxic agents have been initiated in a variety of oncologic

indications; 2 are completed and 4 are ongoing. One phase III combination study in pancreatic cancer was stopped for futility based on predefined boundary rules and other phase III combination studies are completed or are ongoing in the following indications: lung cancer, colorectal cancer, and prostate cancer.

In terms of adverse events (AEs), the most common treatment-emergent AEs (TEAEs) reported for aflibercept monotherapy included asthenia/fatigue, hypertension, headache, nausea, gastrointestinal and abdominal pains, dysphonia, musculoskeletal and connective tissue pains (including back pain, musculoskeletal pain and pain in extremities), decreased appetite, vomiting, hemorrhage (mainly mild to moderate epistaxis), constipation, diarrhea, breathing abnormalities (mainly dyspnea), arthralgia, oedema, proteinuria, cough, fever, myalgia and weight decrease. Target pharmacological exposure has been reached at doses ≥2 mg/kg IV given every 2 weeks. Free aflibercept levels have remained in excess of bound aflibercept levels throughout the dosing intervals at this, or higher, doses. Objective tumor responses and prolonged (>1year) disease stabilization have been reported at dose levels ≥800 µg/kg SC and ≥1 mg/kg IV with aflibercept monotherapy and with aflibercept administered in combination with cytotoxic chemotherapy.

1.3.2 Preclinical data

In vitro, aflibercept binds to human VEGF165 with high affinity. Aflibercept binds to VEGF-A from human, mouse, rat and rabbit with sub-picomolar KD values. The human VEGF-A protein sequence is identical to its counterpart from cynomolgus monkey. Thus, the binding interaction of human and monkey VEGF with aflibercept is the same. As a consequence of the ability of aflibercept to bind to rodent, rabbit and monkey VEGF, these species were used for toxicological investigations. The high binding affinity of aflibercept to VEGF results in the formation of a complex that prevents VEGF from interacting with endothelial cell surface receptors. In vitro, aflibercept blocked VEGF-induced proliferation of endothelial cells and strongly inhibited microvessel outgrowth from rat aorta in vitro. To examine whether effector function plays any role in aflibercept activity, ex vivo cell-based assays were performed. Aflibercept was not able to mediate ADCC or CDC activity in either primary endothelial or tumor cell lines. Based on these results, aflibercept is unlikely to promote either CDC or ADCC in vivo.

In vivo pharmacology studies have indicated that treatment with aflibercept effectively inhibits tumor growth of a wide variety of murine, rat, and human tumor cell lines implanted in mice. Consistent with the inhibition of VEGF, aflibercept treatment of several established tumors results in a rapid decrease in tumor vessel density. Aflibercept exhibits a broad spectrum of anti-tumor activity, being active against 22 out of 27 models tested. Aflibercept also was effective in combination with several widely-used chemotherapeutic agents, including oxaliplatin, 5-FU, irinotecan, paclitaxel, and docetaxel.

Combined drugs (with aflibercept)	Tumor models grafted in mice	Combination results
Docetaxel	Mouse melanoma B16	Enhanced activity
Paclitaxel	Human ovarian adenocarcinoma OVCAR-3	Enhanced activity
5-Fluorouracil	Mouse mammary adenocarcinoma MA 13/C	Synergistic activity
Irinotecan	Human colon carcinoma HCT 116	Synergistic activity
Gemcitabine	Human colon carcinoma HCT 116	Enhanced activity
S-1	Mouse mammary adenocarcinoma MA 13/C	Synergistic activity
Oxaliplatin	Mouse colon adenocarcinoma C51	Synergistic activity
Cisplatin	Mouse colon adenocarcinoma C51	Synergistic activity

Table 1. In vivo antitumor activity of aflibercept in combination with chemotherapy (*Extracted from the Investigator's Brochure Aflibercept (VEGF TRAP, AVE0005).*

Aflibercept treatment was well tolerated in the preclinical tumor models, with no overt toxicities at doses up to 40 mg/kg twice per week. Importantly, increased toxicities were not generally observed in the combinations of aflibercept with chemotherapy. Histological analysis indicated that treatment with aflibercept resulted in the formation of largely avascular and necrotic tumors, demonstrating that tumor-induced angiogenesis was blocked.

Aflibercept was also active in blocking tumor growth in similar animal tumor models in combination with paclitaxel, docetaxel, or radiation, and was synergistic with 5-fluorouracil (5-FU), and irinotecan. Aflibercept as a single agent and in combination with paclitaxel also prevented the formation of ascites in mouse tumor models. Following IV administration in all animal species evaluated, free aflibercept was characterized by a low clearance (0.5 to 3 mL/hr/kg), a low volume of distribution (69 to 226 mL/kg), and a long apparent elimination half-life (48 to 98 hours). Absolute bioavailability of the SC route was 94% and 85% in mice and monkeys, respectively. Aflibercept inhibition of tumor growth in mouse xenograft models was observed at doses ≥2.5 mg/kg. This dose corresponds to free aflibercept Cmax concentrations of 10 µg/mL, about 10-fold higher than bound aflibercept concentrations. This suggests that levels of free aflibercept in excess of bound aflibercept may be necessary for pharmacological activity. Accordingly, the target pharmacological exposure in humans has been proposed to be safe at safely administered doses where maximal bound aflibercept levels are achieved and an excess of free aflibercept is sustained. Aflibercept had no effects on the respiratory function in rats, the renal function in mice and the venous and arterial thrombus formation in rabbits. SC administrations of aflibercept to male mice resulted in significant reductions in microvessel density mainly within the liver, pancreatic islets and thyroid follicles at doses ranging from 2.5 to 25 mg/kg/administration and to a lesser extent in the pituitary gland and adipose tissue. The single SC administration of aflibercept at 2.5 and 25 mg/kg to mice and from 0.5 to 25 mg/kg to rats resulted in a moderate, long lasting but reversible increase in systolic and diastolic blood pressure. The blood pressure in rats treated with aflibercept was lowered by several classes of anti-hypertensive agents, eg, angiotensin converting enzyme inhibitors, calcium channel blockers, an adrenergic receptor

antagonist and modulator of nitric oxide availability. Aflibercept was shown to induce a moderate delay in wound repair and healing from 0.3 mg/kg/administration after IV administration in rabbits. Aflibercept toxicity was evaluated after SC or IV administration for 4 and 13 weeks and IV administration for 26 weeks to (sexually mature and immature) cynomolgus monkeys and SC administration for up to 13 weeks in rats. In a 3-month rat study, the SC administration 3 times per week of aflibercept was associated with morbidity and mortality at 1 and 2 mg/kg. The most prominent microscopic findings were observed in the kidney (mesangial thickening and tubular protein casts) associated with increased BUN and urinary protein levels and decreased serum protein levels. It is likely that the renal toxicity of aflibercept observed in normal immunocompetent rats, which produce antibodies to aflibercept, was due, at least in part, to the deposition of immune complexes in renal glomeruli. Microscopic changes in the femur (decrease and/or loss of bony trabeculae and metaphysical capillaries, thinning of cortical bone) were observed from 1 mg/kg. These bone changes were most likely induced primarily by the pharmacologic effect of aflibercept.

In the primate studies, main compound-related microscopic findings were observed in the bone, nasal cavity, kidney, ovary, and adrenal gland. In the bone, aflibercept-induced effects consisted of thickening of the growth plate and osteocartilaginous exostoses observed on the axial and appendicular skeleton that correlated with hunched posture noted at clinical examination. In the nasal cavities, a minimal to moderate degeneration/regeneration of the respiratory and olfactory epithelium was often associated with hemorrhage and suppurative exudate, and atrophy/loss of nasal septum and/or turbinates. Histopathological findings in the kidneys (increased glomerular mesangial matrix) were associated in a few animals with decreased serum total protein and albumin levels and increased BUN and urine protein and/or microalbumin levels. In the ovaries, the decreased number of maturing follicles, granulosa cells and/or theca cells was associated with an overall inhibition of the female reproductive function. The histopathological changes in the bone growth plate and ovary were considered related to the pharmacological activity of aflibercept. In the adrenals, a decreased vacuolation of adrenal zona fasciculata cells with cytoplasmic eosinophilia was observed. In addition, focal vascular proliferation/degeneration was noted in a range of organs including in particular the digestive system, urinary bladder, heart and brain of a few monkeys. Main clinical pathology changes consisted in slight increases in red blood cell parameters, fibrinogen, globulin, BUN and decreases in albumin. Urinary changes associated with kidney microscopic findings consisted of increased urinary protein and/or microalbumin. In addition, increased liver enzymes levels were noted in a few monkeys with portal inflammation and necrosis. Aflibercept administration resulted also in a decrease in sperm motility and increased incidence of abnormal spermatozoa morphology. Most aflibercept-related findings were noted from the lowest doses tested (1.5 to 3 mg/kg/administration in most instances. With the exception of skeletal and nasal cavity findings, aflibercept-related findings were reversible within 5 months.

Aflibercept was not highly immunogenic in monkeys treated for 3 months by either the SC or IV route while the incidence of antibody response increased in monkeys treated for 6 months. When administered IV to pregnant rabbits (as a 30-minute infusion once daily, on gestation Days 6, 9, 12, 15, and 18 - a total of 5 administrations) aflibercept induced minimal to moderate maternal toxicity, abortion, and embryolethality at 60 mg/kg/administration. At the moment, there is no clinical data about teratogenicity or embryolethality for subjects fathered by male subjects treated with Aflibercept. External, visceral, and/or skeletal malformations were observed in fetuses from pregnant rabbits

treated from 3 mg/kg/administration (approximately 1.3 times the exposure in patients treated at the recommended human dose). Aflibercept did not demonstrate cross-reactivity with any of a broad panel of representative human tissues by immunohistochemical staining at concentrations (5 and 25 µg/mL) for which there were strong specific bindings to VEGF-expressing CHO cells.

1.3.3 Pharmacokinetics

Clinical pharmacokinetics of single agent aflibercept were evaluated following SC and IV administration. Pharmacokinetics of SC administration have been characterized by relatively little fluctuation between peak and trough free aflibercept levels, and by non-linear PK at doses ranging from 0.025 to 0.8 mg/kg. The pharmacokinetics of IV infusion have been characterized by a relatively low volume of distribution, and no or little accumulation during a biweekly regimen. Free aflibercept levels have remained in excess of bound aflibercept levels throughout the dosing intervals at >2 mg/kg IV biweekly dose levels, a target pharmacological exposure that correlated with antitumor efficacy in the preclinical setting. When administered IV, the PK of free aflibercept was non-linear following administration of <2 mg/kg doses, but became linear between the 2 mg/kg and 7 mg/kg dose levels. The PK of free aflibercept was characterized by high fluctuation and no or little accumulation when given every 2 weeks. The concentrations of bound aflibercept increased between the 0.3 mg/kg and 2 mg/kg dose levels, and then plateaued between the 2 mg/kg and 7 mg/kg dose levels, suggesting that free aflibercept was available in a sufficient amount to bind all endogenous VEGF in this higher dose range.

1.3.4 Pharmacodynamics

VEGF is a mitogen for endothelial cells that promotes formation of new blood vessels that are required for normal and neoplastic tissue growth. The importance of VEGF-mediated angiogenesis in tumor biology is underscored by numerous observations that VEGF and/or its receptors are highly expressed in a variety of tumor types. Aflibercept inhibits the biological actions of VEGF by preventing its interaction with 2 VEGFRs, VEGFR1 (Flt-1) and VEGFR2 (Flk-1), predominantly located on the vascular endothelium. In vitro, aflibercept binds to human VEGF165 with a high affinity (KD = 0.50 pM) and a 1:1 stochiometry. It also binds the other ligands of VEGFR1, including PlGF with good affinity (KD = 39 pM). In vitro*,* aflibercept binding to VEGF prevented VEGF-induced phosphorylation of VEGFR2 and VEGF-induced proliferation of endothelial cells. Aflibercept does not mediate antibody-dependent cell cytotoxicity (ADCC) in either primary human umbilical vein endothelial cells or tumor cell lines. No CDC induced by aflibercept was observed in either endothelial cells or tumor cell lines. Based on these results aflibercept would not be predicted to promote either CDC or ADCC activities in vivo. In vivo pharmacology studies have indicated that treatment with aflibercept effectively inhibits tumor growth of a wide variety of rodent and human tumor cell lines implanted either SC or orthotopically in mice. Aflibercept treatment inhibited the growth of tumors representing a variety of tissue types including melanoma, glioma, rhabdomyosarcoma, Wilms tumor, neuroblastoma, Ewing sarcoma, lymphoma, and ovarian, pancreatic, prostate, mammary, gastric, and colon tumor tissues, with a broad pharmacological index. Antitumor activity was observed from 2.5 to 40 mg/kg. Aflibercept treatment significantly decreased the vessel density in tumors, an indication that tumor-induced angiogenesis was inhibited. The PK profile of aflibercept was assessed by measuring both the free aflibercept and the aflibercept bound to VEGF. At doses where aflibercept was active in mouse tumor models, free aflibercept in serum was found in

large excess compared to a steady state level of ~2 µg/mL for bound aflibercept; VEGF was captured by aflibercept and found as complexes in the blood circulation. Because VEGF has a major role in vascular leakage leading to ascites formation, aflibercept was tested against tumor models producing ascites in mice: a human ovarian cancer model (OVCAR-3) inoculated into the peritoneum, and a renal cell carcinoma model (RENCA) implanted in the kidney. In a first study with the RENCA model, aflibercept at 40 mg/kg completely prevented ascites formation and inhibited tumor growth. In a second study, aflibercept was evaluated in dose response. No measurable ascites developed and tumor burden was reduced in mice treated at 10 and 25 mg/kg of aflibercept. When OVCAR-3 cells were implanted into the peritoneum of nude mice, animals developed significant tumor burden, as well as ascites, over a period of approximately 8 weeks. Treatment with aflibercept 25 mg/kg twice weekly for 5 weeks, initiated 2 weeks after tumor cell inoculation, prevented the formation of ascites in all animals and also decreased tumor burden in these mice by greater than 50%. Aflibercept was also active in blocking tumor growth in similar animal tumor models when administered in combination with paclitaxel, docetaxel, gemcitabine, or radiation. Synergism was observed with 5-FU, S-1, cisplatin, and irinotecan.

1.3.5 Summary of clinical data

1.3.5.1 *Phase I*

In the single agent Phase I study of SC aflibercept (TED6113), 2 patients, 1 with heavily pretreated bronchoalveolar carcinoma and another with medullary thyroid cancer, improved either subjectively and/or radiologically at a dose of 800 µg/kg with prolonged disease stabilization of over 2 years.

In the Phase I studies of IV single agent aflibercept (TED6115 and TED6116), 3 patients have had RECIST-defined partial tumor responses: 1 patient with metastatic malignant thymoma treated at the 3 mg/kg dose level, and 2 patients with ovarian cancer treated at the 7 mg/kg dose level. Two patients with renal cell cancer, treated at the 1 mg/kg and 2 mg/kg dose levels, respectively, have had prolonged (exceeding one year) stable disease (SD).

In the combination Phase I studies, radiological tumor responses per RECIST criteria have been observed across different tumor types (breast, cholangiocarcinoma, gastric and gastroesophageal junction, pancreas, colorectal, thyroid and ovarian cancers) and aflibercept doses.

1.3.5.2 *Phase II*

Single agent Phase II studies in previously treated patients (2 or more lines of chemotherapy treatment) have evaluated aflibercept administered IV every 2 weeks in ovarian and lung cancer (ARD6122, ARD6123) with response rate (RR) as the primary endpoint. In ARD6123, a single agent, single arm study of aflibercept at 4 mg/kg in advanced, heavily pretreated non-squamous non-small cell lung cancer (NSCLC), an Investigator-reported overall response rate (ORR) of 2% was observed, along with a median progression free survival (PFS) of approximately 12 weeks, and stable disease (SD) for ≥60 days of approximately 30%. The median overall survival (OS) was approximately 6 months. In ARD6122, a randomized, double-blind study of aflibercept at 2 mg/kg or 4 mg/kg in advanced ovarian cancer, the Investigator-reported ORR was 3.8% in the 2 mg/kg arm and 7.3% in the 4 mg/kg arm; the median PFS in both arms was approximately 18 weeks, and SD for ≥60 days of approximately 45% and 37% in the 2

mg/kg and 4 mg/kg arms, respectively. The median OS was approximately 55 weeks in the 2 mg/kg arm and 50 weeks in the 4 mg/kg arm.

Additionally, single agent Phase II studies have explored the efficacy of aflibercept in prolonging the time to repeat paracentesis in advanced ovarian cancer patients with symptomatic malignant ascites (SMA). Study ARD6772 explored 4 mg/kg IV every 2 weeks, and showed at least a doubling of the primary endpoint (time to repeat paracentesis compared to baseline) for 62% of the patients. In a similar patient population in Study EFC6125 (a randomized double-blind study of aflibercept 4 mg/kg IV every 2 weeks versus placebo), aflibercept significantly prolonged the time to repeat paracentesis as compared with placebo, from 23.3 to 55.1 days for a difference of 31.8 days (95% CI [10.56 - 53.05]; p-value = 0.0019).

1.3.5.3 *Phase III*

Preliminary results of the VELOUR trial were recently reported (Tabernero 2011; Van Cutsem 2011). In the VELOUR study 1,226 patients with mCRC who had progressed after first-line treatment containing oxaliplatin were randomized to FOLFIRI plus aflibercept (4 mg/kg IV, day 1, every two weeks) or to FOLFIRI and placebo. After a median follow-up of 22.3 months, there was a statistically significant survival advantage for patients treated with aflibercept and FOLFIRI (HR 0.82; *p*=0.0032). The primary analysis showed a median OS of 13.5 months in the aflibercept arm and 12.1 months in the placebo arm. PFS also favored aflibercept (6.9 vs. 4.7 months; HR, 0.76; 95% CI, 0.58-0.99; *p*=0.00007), as did response rate (19.8% vs. 11.1%; *p*=0.0001) (Van Cutsem 2011).

Subgroup analysis of stratification factors showed a similar effect of aflibercept on OS in patients with Eastern Cooperative Oncology Group (ECOG) performance status (PS) 0, 1 or 2. The investigators observed no significant differences between patients treated with prior bevacizumab and those with no previous exposure to bevacizumab. The HR among the 30% of the study population that had already been treated with bevacizumab was 0.86, and the HR among the 70% of patients without prior bevacizumab exposure was 0.79 (*P*=0.723). Analysis of survival by demographic characteristics revealed a similar benefit for patients younger than age 65 years or age 65 and older, for men and for women, and for patients from all geographic regions represented in the trial. Tests for interaction again confirmed the consistency of the results. Consistent with the results for OS, PFS did not differ significantly by previous bevacizumab exposure. Patients who had not been previously treated with bevacizumab had a median PFS of 6.9 months with aflibercept versus 5.4 months with placebo. In the subgroup of bevacizumab-treated patients, median PFS was 6.7 months with aflibercept and 3.9 months with placebo. The test for interaction showed no significant differences (*p*=0.6954) (Tabernero 2011).

1.3.6 Safety

1.3.6.1 *Single agent studies*

All grade treatment emergent adverse events (TEAEs) very commonly reported across single agent oncology trials (N=404) regardless of relationship to study drug, in > 10% of patients, include asthenia/fatigue, hypertension, headache, nausea, abdominal pain, dysphonia (hoarse voice), musculoskeletal and connective tissue pains (including back pain, musculoskeletal pain and pain in extremities), decreased appetite, vomiting, hemorrhage (including mainly mild to moderate epistaxis), constipation, diarrhea, breathing abnormalities (mainly dyspnea), arthralgia, edema, proteinuria, fever, and

weight decrease. Additionally, all grade TEAEs commonly reported across single agent oncology trials regardless of relationship to study drug, in >5% of patients, include mucosal inflammation, dizziness, rash, urinary tract infection, insomnia, dehydration, hyponatremia, abdominal distension and flatulence, stomatitis, and ulceration, pharyngolaryngeal pain, gastrointestinal obstruction and stenosis, depression, dyspepsia, anxiety, muscle spasm, paraesthesia, dry mouth, and pneumonia. Grade 3-5 events regardless of relationship experienced by >2% of patients included hypertension, asthenia/fatigue, breathing abnormalities (dyspnea), abdominal pain, vomiting, gastrointestinal obstruction and stenosis, dehydration, decreased appetite, headache, diarrhea, nausea, musculoskeletal and connective tissue pains, pneumonia, constipation, and arthralgia. Additionally, in the overall system order class (SOC) of infections and infestations, Grade 3-5 infections were reported for 7.7% of patients although no individual diagnosis (other than pneumonia) were reported for >2% of patients. The majority of diagnosis terms listed above has been reported as both non-serious and serious adverse events. In addition to these diagnoses all grade SAEs reported in single agent oncology studies for more than 1 patient regardless of relationship and excluding events reported by investigator as directly attributed to disease progression also included: cardiac failure, renal failure (none requiring dialysis, cases confounded by ureteral obstruction, dehydration or sepsis), gastrointestinal hemorrhage, intestinal perforation, hemoptysis, fistula (includes tracheoesophageal and enterocutaneous fistula), thrombocytopenia (confounded by new onset AML, or systemic infection), hypoglycemia, reversible posterior leukoencephalopathy syndrome (RPLS), death/sudden death, cerebral ischemia, deep vein thrombosis and pulmonary embolism. In the single agent studies, although these AEs may be drug related, an association with the underlying disease in a heavily pretreated patient population (majority of the patients enrolled in third line (or more) ovarian cancer and non-small cell lung cancer indications) cannot be excluded.

1.3.6.2 *Combination studies*

All grade TEAEs commonly reported across trials exploring aflibercept in combination with other anticancer drugs are similar to the single agent drug safety profile but also reflect common toxicities associated with the drugs used in these combination regimens. Some anticancer drugs being explored in Phase I combination studies include: docetaxel, gemcitabine, erlotinib, irinotecan, oxaliplatin, cisplatin, and fluorouracil. AEs commonly reported in the combination trials included asthenia/fatigue, gastrointestinal adverse events (diarrhea, nausea, vomiting, constipation, stomatitis), hypertension, proteinuria, epistaxis, dysphonia, pain (including abdominal pain, musculoskeletal pain, and back pain), headache, dyspnea, decreased appetite, neuropathy, mucosal inflammation, electrolyte changes (including hypomagnesemia), and alopecia. Hematological abnormalities commonly reported in the combination trials included neutropenia, anemia, and thrombocytopenia.

In studies where aflibercept was administered in combination with chemotherapy SAEs reported regardless of relationship in at least 10 patients included pyrexia, febrile neutropenia, diarrhea, small intestinal obstruction, hypertension, dyspnea, neutropenia and pulmonary embolism. SAEs reported for 2 or more patients in combination studies but not for 2 or more patients in single agent studies included febrile neutropenia, neutropenia, thrombotic microangiopathy (reported as SAE in 1 patient in single agent Study ARD6122), colitis, gastroesophageal reflux disease, hypersensitivity, syncope, cerebral hemorrhage, somnolence, confusional state, hematuria, nephrotic syndrome

(reported as SAE for only 1 patient and as non-serious event in a second patient in single agent Study ARD6122), and hypotension.

The most frequent AEs, with ≥5% difference in incidence between treatment arms, excluding anti-VEGF class events from the VELOUR phase III trial evaluating FOLFIRI +/- aflibercept as second-line therapy in MCRC are shown in table 2.

The contribution of aflibercept to the frequency and severity of anti-VEGF associated events was explored in the VELOUR phase III trial (Table 3,4).

**Safety Population, % of patients**	**Placebo, N = 605**		**Aflibercept N = 611**	
	All Grades	Grade 3-4	All Grades	Grade 3-4
Diarrhea	56.5	7.8	69.2	19.3
Neutropenia	56.3	29.5	67.8	36.7
Complicated neutropenia	-	2.8	-	5.7
Asthenic conditions	50.2	10.6	60.4	16.9
Stomatitis & ulceration	34.9	5.0	54.8	13.7
Thrombocytopenia	33.8	1.7	47.4	3.3
Infections	32.7	6.9	46.2	12.3
Decrease appetite	23.8	1.8	31.9	3.4
Weight decrease	14.4	0.8	31.9	2.6
Palmar plantar erythrodysaesthesia	4.3	0.5	11.0	2.8
Skin hyperpigmentation	2.8	0	8.2	0
Dehydration	3.0	1.3	9.0	4.3

Table 2. Most frequent AEs in the VELOUR phase III trial (excluding anti-VEGF class events) (Van Cutsem 2011)

**Safety population, % of patients**	**Placebo/FOLFIRI, N=605**		**Aflibercept/FOLFIRI, N=611**	
	All grade	Grade 3/4	All grade	Grade 3/4
Proteinuria	40.7	1.2	62.2	7.9
Hypertension	10.7	1.5	41.4	19.3
Haemorrhage	19.0	1.7	37.8	2.9
GI origin	5.1	1.0	10.0	2.0
Dysphonia	3.3	0	25.4	0.5
Headache	8.8	0.3	22.3	1.6
Venous thromboembolic event	7.3	6.3	9.3	7.9
Arterial thromboembolic event	1.5	0.5	2.6	1.8
Fistula	0.5	0.2	1.5	0.3
Compromized wound healing	0.8	0	0.5	0.3
GI perforation	0.5	0.3	0.5	0.5

Table 3. Anti-VEGF associated events in the VELOUR trial (overall population) (Van Cutsem 2011)

**Safety population, % of patients**	**No prior bevacizumab, N=424**		**Prior bevacizumab, N=187**	
	All grade	Grade 3/4	All grade	Grade 3/4
Proteinuria	62.5	7.5	61.5	8.6
Hypertension	42.2	20.5	39.6	16.6
Haemorrhage	37.3	2.8	39.0	3.2
GI origin	9.9	1.7	10.2	2.7
Dysphonia	27.8	0.5	19.8	0.5
Headache	23.1	1.9	20.3	1.1
Venous thromboembolic event	9.0	7.8	10.2	8.0
Arterial thromboembolic event	2.4	1.7	3.2	2.1
Fistula	1.9	0.5	0.5	0
Compromized wound healing	0.2	0.2	1.1	0.5
GI perforation	0.7	0.7	0	0

Table 4. Anti-VEGF associated events by prior use of bevacizumab in patients receiving aflibercept (Van Cutsem 2011)

In the VELOUR phase III trial, the safety profile of aflibercept was acceptable and consistent with known anti-VEGF adverse effects. Adding aflibercept increases the specific CT related toxicity in the combination arm: neutropenic complications and diarrhea/stomatitis. And, prior treatment with an anti-angiogenic agent does not appear to significantly impact the safety profile of aflibercept.

1.4 Cytokines and angiogenic factors (CAF) in metastatic colorectal cancer

There is a broad variety of CAF described in mCRC with conflicting results regarding prognostic or predictive value for single analysis at baseline of for dynamic analyses during treatment. In the already mentioned trial by Kopetz et al several proangiogenic cytokines were elevated before progression, notably basic fibroblast growth factor (bFGF), placental like growth factor (PlGF), hepatocyte growth factor (HGF), matrix metallo peptidase 9 (MMP9) and VEGF C (Kopetz, Hoff et al. 2010). PlGF could be confirmed in a retrospective cohort presented by the same group at ASCO 2011 and by the GONO group, whereas VEGF-C could not be validated (Lieu 2011; Loupakis, Cremolini et al. 2011). Interestingly, the increase of PlGF is limited to treatment with bevacizumab and does not occur during treatment with chemotherapy alone (Lieu 2011).

PlGF is a member of the vascular endothelial growth factor (VEGF) family of proangiogenic factors and its overexpression has been linked to pathological angiogenesis. PlGF levels have been shown to be elevated in a number of different tumor types, including CRC, breast cancer (BC) and renal cell carcinoma (Matsumoto, Suzuki et al. 2003; Chen, Hsieh et al. 2004; Parr, Watkins et al. 2005; Wei, Tsao et al. 2005; Escudero-Esparza, Martin et al. 2009; Wei, Liang et al. 2009; Escudero-Esparza, Martin et al. 2010). Furthermore, high serum PlGF and PlGF mRNA levels are correlated with poor outcomes and higher recurrence rates after curative resection in patients with CRC, hepatocellular carcinoma and gastric cancer (Chen, Hsieh et al. 2004; Parr, Watkins et al. 2005; Wei, Tsao et al. 2005; Ho, Chen et al. 2007; Escudero-Esparza, Martin et al. 2009; Wei, Liang et al. 2009). Moreover, PlGF levels were elevated in patients after anti-angiogenic therapy and prior to disease progression in patients with CRC (Batchelor, Sorensen et al. 2007; Rini, Michaelson et al. 2008; Willett, Duda et al. 2009; Kopetz, Hoff et al. 2010; Nikolinakos, Altorki et al. 2010; Lieu 2011; Loupakis, Cremolini et al. 2011). These data indicate a major role of PlGF in resistance to anti angiogenic treatment, particularly to bevacizumab.

1.5 Rationale for the early switch regimen

Up-regulation of VEGF-A-independent angiogenic pathways during continuous inhibition of VEGF signaling in the presence of e.g. tumor hypoxia might be a potential explanation of resistance to treatment with bevacizumab. Recent studies reported rising levels of proangiogenic cytokines (PlGF, HGF, and bFGF) during treatment with bevacizumab and before disease progression, which could not be demonstrated with chemotherapy alone (Kopetz, Hoff et al. 2010; Lieu 2011; Loupakis, Cremolini et al. 2011). PlGF, bFGF, HGF and MMP-9 levels during bevacizumab treatment were found to be increased to 25-100% over baseline just before progression (figure 2 and 3) and might thus be useful components of a composite marker for early detection of progression.

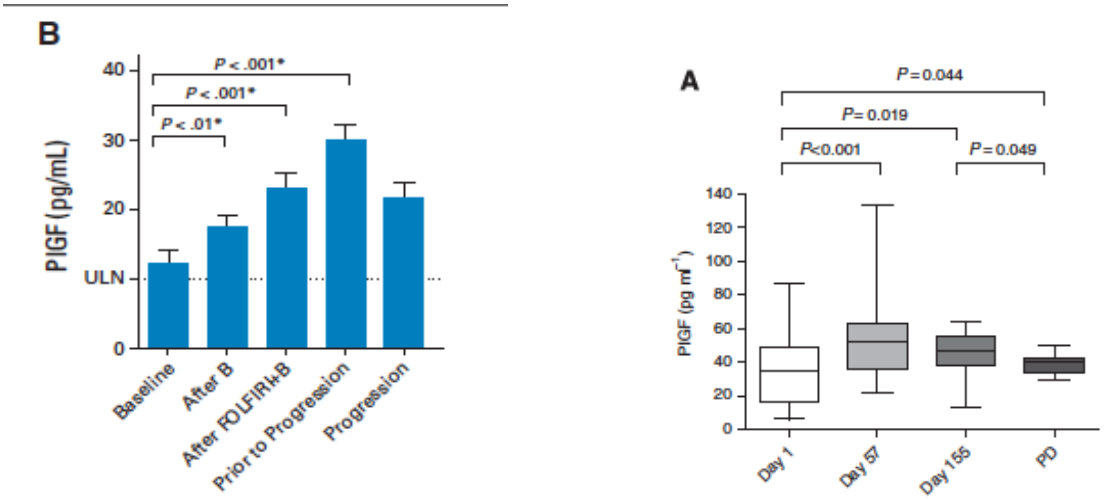

Figure 2 Patterns of PlGF levels during bevacizumab and chemotherapy (Kopetz, Hoff et al. 2010; Loupakis, Cremolini et al. 2011)

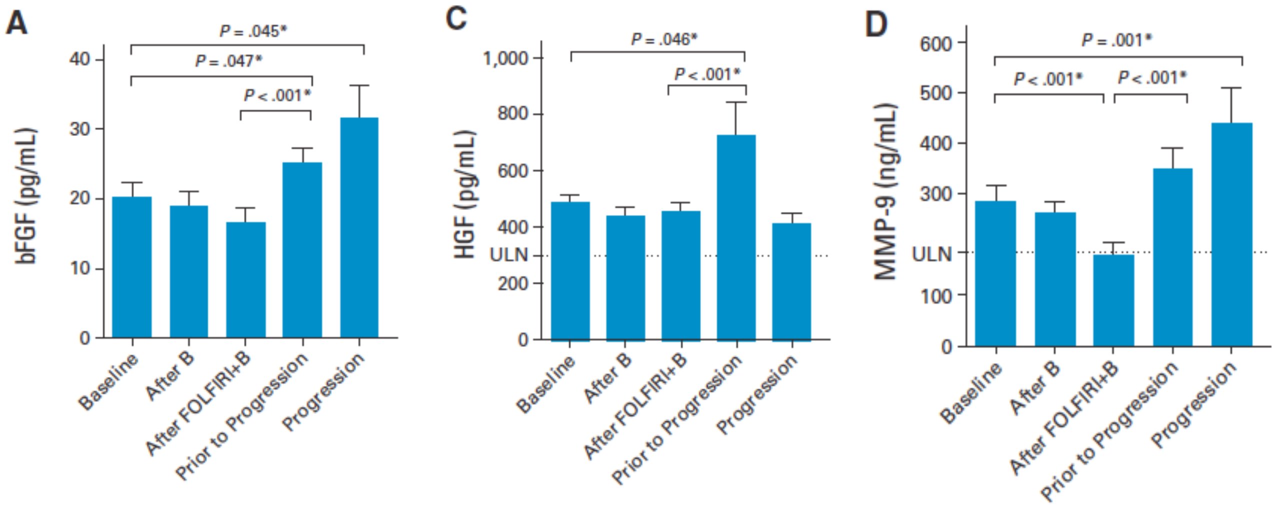

Figure 3 Patterns of bFGF, HGF and MMP-9 levels during bevacizumab and chemotherapy (Kopetz, Hoff et al. 2010; Loupakis, Cremolini et al. 2011)

Currently, results of two large second line phase III trials (VELOUR and TML) have challenged angiogenic treatment strategies in mCRC (Tabernero 2011; Van Cutsem 2011; Arnold 2012). Both trials demonstrated the beneficial impact on survival for continuation of antiangiogenic treatment, with either aflibercept after bevacizumab or continuation of bevacizumab beyond progression with a changed chemotherapy backbone. In the VELOUR trial with overall 1226 patients, treatment with aflibercept plus FOLFIRI was superior in terms of OS (12.1 to 13.5 months, HR 0.817, 95% CI 0.713-0.937), RR and PFS compared to FOLFIRI alone after failure of FOLFOX. Furthermore, efficacy of aflibercept was in the same range even after pretreatment with bevacizumab, albeit not statistical significant due to the smaller number of patients (n=373) (11.7 to 12.5 months, HR 0.862, 95% 0.673-1.104) (Tabernero 2011; Van Cutsem 2011). In the TML trial presented at ASCO 2012 continuation of bevacizumab beyond progression with changed chemotherapy backbone either oxaliplatin-based to irinotecan-based or vice versa demonstrated significant superior OS (9.8 to 11.2 months, HR 0.81; 95% CI 0.69–0.94) and PFS compared to chemotherapy alone (Arnold 2012).

Aflibercept targets VEGF isoforms A and B and PlGF and might thus have a stronger antiangiogenic effect compared to bevacizumab. Change of angiogenic strategies might be effective as recently shown with bevacizumab adapted cell lines (after 3 months of bevacizumab treatment), expressing higher levels of PlGF and VEGF A-C and being more invasive and migratory than control cells, which could be reversed by VEGFR targeting (Fan, Samuel et al. 2011).

The concept of maintaining VEGF inhibition and change chemotherapy in case of radiologic progression has been proven in the TML trial. However, early change of bevacizumab to the potentially more effective angiogenesis inhibitor aflibercept in case of forthcoming progression, albeit not yet radiologically visible, maintaining chemotherapy might be beneficial in terms of progression free survival and thus delay change to second line chemotherapy. The impact of this marker-driven treatment modification on efficacy and CAF is unknown and will therefore be evaluated in the current trial.

1.6 Rationale for the analysis of cytokines and angiogenic factors and other evaluations

There is a broad variety of cytokines and angiogenic factors (CAF), which might have a prognostic or predictive role either before or during treatment with different anti-angiogenic agents (bevacizumab and aflibercept) and different chemotherapy backbones and furthermore during the change of one of both treatments (anti-angiogenic agent or chemotherapy) maintaining the other.

Predictive value of pre-therapeutic VEGF levels for treatment with bevacizumab in breast cancer was recently reported (AVADO trial; Miles et al. 2010). Patients with high circulating VEGF levels showed a trend towards increased PFS compared to patients with low VEGF levels. In contrast pre-treatment levels of other tested biomarkers including VEGFR-1, ICAM-1 and E-selectin had no predictive impact in this study (Miles et al., 2010). However, predictive value of VEGF seems to be inconsistent in different types of cancer and is intensely debated in current literature (Jain, Duda et al. 2009). In metastatic colorectal cancer low pre-therapeutic serum levels of angiopoietin-2 (Ang-2) were correlated with a higher response rate and longer PFS when compared to low serum levels in patients treated with bevacizumab (Goede, Coutelle et al.).

A recently published study indicates predictive value of different factors involved in angiogenesis including delta-like ligand 4 (DLL4), neuropilin-1 (Npn-1) and vascular endothelial growth factor C (VEGF-C) and VEGF-D in patients treated with bevacizumab combined with chemotherapy (Jubb, Miller et al. 2011; Weickhardt 2011).

Preclinical findings further suggest that anti-angiogenic treatment, after a transient period of vessel normalization, elicits intratumoral hypoxia due to vessel pruning (Fischer, Jonckx et al. 2007; Bergers and Hanahan 2008; Fischer, Mazzone et al. 2008; Paez-Ribes, Allen et al. 2009). This hypoxia induces resistance and metastasis via different mechanisms including secretion of alternative cytokines including osteopontin, which fosters tumor progression and metastasis (Casanovas, Hicklin et al. 2005; Fischer, Jonckx et al. 2007; Ebos, Lee et al. 2009; Paez-Ribes, Allen et al. 2009). Hypoxia also leads to increased attraction of pro-angiogenic myeloid cells including $CD11b^{+}Gr1^{+}$ cells (Shojaei, Wu et al. 2007; Murdoch, Muthana et al. 2008). It was recently elucidated, that $CD11b^{+}Gr1^{+}$ cells comprise a heterogeneous population of myeloid cells that are recruited from the bone marrow to tumors, where they confer resistance to anti-angiogenic treatment in experimental tumor models (Shojaei, Wu et al. 2007; Shojaei, Wu et al. 2007). G-CSF, produced by tumors, induces mobilization and recruitment of these

myeloid cells (Ebihara, Xu et al. 2000; Shojaei, Wu et al. 2007). Third hypoxia remarkably also results in distinct mechanisms of escape towards VEGF(R)Is, namely (i) selection of hypoxia-resistant cancer cells, less dependent on angiogenesis; (ii) favouring of intrinsically hypoxia-resistant cancer stem cells and (iii) increasing invasive and migratory phenotype of cancer cells. Of note, hypoxia also compromises efficacy chemotherapy and radiotherapy (Brown 2007). A growing body of evidence indicates that hypoxia represents a worse prognostic marker in a variety of cancers and that it compromises response to chemo- and radiotherapy (Wouters and Brown 1997; Harris 2002; Vaupel and Mayer 2007; Jubb and Harris 2010). Direct measurements of tumor tissue hypoxia are impossible in cancer patients due to their invasiveness. Hence, reliable surrogate markers for hypoxia were developed including HIF1a, CAIX and Glut-1 (Jubb and Harris 2010). These markers correlate with patient outcome in different cancers (Vaupel and Mayer 2007; Jubb and Harris 2010) and circumstantial evidence from a small Phase II study in glioblastoma patients treated with irinotecan and Bev indicates association of high expression of CAIX with poor survival (Sathornsumetee, Cao et al. 2008).

Molecular markers like BRAF and KRAS/NRAS will be additionally analyzed in the trial. Recently, presented data has shown a strong prognostic value for BRAF and potentially for KRAS/NRAS as well, although at least for the latter results are still divergent (Maughan, Adams et al. 2011; Price, Hardingham et al. 2011; Bokemeyer, Cutsem et al. 2012).

In case other molecular markers, which could be analyzed in paraffin embedded tumor tissues might gain importance during conduction of the trial further analyses will be performed.

Evaluation of quality of life during chemotherapy is an important issue especially in the palliative setting using the well-established EORTC QLQ C30 and the colorectal module CR29.

1.7 Risk-Benefit Assessment

Patients included in this trial suffer from metastatic, mainly incurable colorectal cancer. Median overall survival of these patients is about 20 months. The current trial evaluates an early switch of angiogenic treatment, which will not impair sequence of treatment and receipt of all available drugs in this disease setting. In regard of a symptomatic or even life endangering clinical progression an early switch might be beneficial for the patient. Furthermore, change of angiogenic strategy maintaining the chemo backbone will delay definite change to second line and might thus be relevant for treatment sequence and overall survival.

2. Study Objective

The primary objective of the two phase PERMAD trial is the evaluation of the impact of a personalized marker-driven treatment approach with early detection of progression and modification of treatment on cytokines and angiogenic factors (CAF) and efficacy.

In regard of the two parts, the primary objective of the run-in phase with conventional switch of chemotherapy together with the anti-angiogenic agent is the determination of a distinct cytokines and angiogenic factor (CAF) profile during treatment with FOLFOX and bevacizumab, which allows early detection/prediction of progressive disease. The primary objective of the marker-driven randomized part with marker-driven switch of anti-angiogenic agent and maintenance of chemotherapy is the evaluation of the efficacy of an early marker-driven switch of anti-angiogenic treatment (bevacizumab to aflibercept)

maintaining the chemotherapy backbone until definite radiological progression compared to a conventional treatment approach of changing chemotherapy and antiangiogenic agent at time of radiologic progression.

Secondary objectives of both parts are prognostic and predictive value of CAF at baseline and the changes during treatment, efficacy, tolerability and patient related outcome.

3. Study Design

This is a multicentre, multinational, open labeled, prospective, randomized, controlled phase II study designed to assess the clinical utility of an early marker driven change of anti-angiogenic treatment (bevacizumab to aflibercept) maintaining the chemotherapy backbone until definite radiological progression in first line treatment of patients with metastatic colorectal cancer.

After completing the run in phase of the study, with at least 30 patients completing their first line treatment (due to progression, secondary resection or toxicity) and being evaluable for CAF analyses, the results will be reviewed by an Independent Data Monitoring Comittee (IDMC). Based on that review the decision to continue with, modify or cancel the randomized part will be made.

3.1 Primary Endpoint

The primary endpoint of the run-in phase with conventional switch of chemotherapy together with the anti-angiogenic agent is:

- Progression free survival (PFS1) of first line treatment

The primary endpoint of the randomized part with marker-driven switch of antiangiogenic agent and maintenance of chemotherapy is:

- Progression free survival rate at 6 months (PFSR@6) after first cycle after randomization.

3.2 Secondary Endpoints

The secondary endpoints include:

- Predictive value of cytokines and angiogenic factor (CAF) particularly PIGF and VEGF-B for early detection of progression during treatment with chemotherapy and bevacizumab
- Determination and validation of a CAF profile based on PIGF and VEGF-B predicting tumor progression before radiologic progression
- PFS1, after first cycle after randomization (PFSr) and of second line treatment (PFS2)
- Time to randomization (TTR)
- Overall survival (OS)
- Overall response rate (RR) according to RECIST v1.1
- Secondary resection rate (sRR)
- Toxicity (Safety assessments will include physical examinations (blood pressure, heart rate, respiratory rate), vital signs, ECOG, clinical laboratory profile and monitoring of adverse events, according to NCI CTCAE v4.03)
- Quality of life using the EORTC QLQ-C30 and the modules CR29
- Changes in CAF during early marker-driven switch and conventional treatment approach

- Prognostic value of CAF at baseline and/or during treatment

4. Study Population

All patients with metastatic or recurrent colorectal cancer admitted for chemotherapy will be reviewed for study eligibility. After checking suitability to enter the study, patients who agree to participate must have signed the informed consent form before undergoing any study related procedures or treatment.

4.1 Number of Patients

200 patients will be enrolled in this study. Patients withdrawn from the trial will not be replaced.

4.2 Selection criteria

Patients will be eligible for the study if they fulfill the following entry criteria.

4.2.1 Inclusion criteria

1. Patients with histologically confirmed diagnosis of colorectal cancer presenting with unresectable stage IV (UICC) disease (primary tumor may be present)
2. Patients with at least one measurable lesion, with size > 1 cm (RECIST v1.1)
3. ECOG Performance status ≤ 2
4. Life expectancy > 3 months
5. Age ≥18 years.
6. Haematologic function: ANC ≥ 1.5 x 10^9/L, platelets ≥ 100 x10^9/L, hemoglobin ≥ 9 g/dl or 5.59 mmol/l
7. Patients not receiving therapeutic anticoagulation must have an INR < 1.5 and aPTT < 1.5 x ULN within 7 days prior to enrollment. The use of full dose anticoagulants is allowed as long as the INR or aPTT is within therapeutic limits (according to the medical standard in the institution) and the patient has been on a stable dose for anticoagulants for at least two weeks at the time of enrollment.
8. Adequate liver function as measured by serum transaminases (AST & ALT) ≤ 2.5 x ULN (in case of liver metastases < 5 x ULN) and total bilirubin ≤ 1.5 x ULN
9. Adequate renal function: Serum creatinine ≤ 1.5 x ULN
10. Signed, written informed consent
11. At least 6 months after completion of adjuvant chemotherapy.

4.2.2 Exclusion criteria

1. Treatment with any other investigational agent within 30 days prior to entering this study.
2. Prior systemic or local treatment of metastatic disease.
3. Prior adjuvant or neo-adjuvant chemotherapy/radiotherapy completed less than 6 months prior to study entry.
4. Pre History or evidence upon physical/neurological examination of CNS disease (unrelated to cancer) (unless adequately treated with standard medical therapy) e.g. uncontrolled seizures.
5. Fertile women (< 1 year after last menstruation) and men of childbearing potential unwilling or unable to use effective means of contraception (adequate: intrauterine device, long-acting injection, hormon implant, vasectomy) during treatment and for 6 months after the end of treatment.
6. Pregnancy or lactation.

7. Positive serum pregnancy test within 7 days of starting study treatment in pre-menopausal women and women < 1 year after the onset of menopause. Note: a negative test has to be reconfirmed by a urine test, should the 7-day window be exceeded.
8. Past or current history (within the last 2 years prior to treatment start) of other malignancies except metastatic colorectal cancer (patients with curatively treated basal and squamous cell carcinoma of the skin or *in situ* carcinoma of the cervix are eligible).
9. Peripheral neuropathy NCI CTCAE-grade ≥ 1
10. Known DPD-insufficiency.
11. Active inflammatory bowel disease or other bowel disease causing chronic diarrhea (defined as > 4 loose stools per day)
12. History of interstitial lung disease (e.g., pneumonitis or pulmonary fibrosis) haemoptoe or evidence of interstitial lung disease on baseline CT scan.
13. Serious, non-healing wound, ulcer or bone fracture.
14. Thrombosis or severe bleeding within 6 months prior to entry into the study (except for bleeding of the tumor before its surgical resection) and no evidence of bleeding diathesis or coagulopathy.
15. Urine dipstick for proteinuria ≥ 2+. If urine dipstick is ≥ 2+, 24-hour urine must demonstrate ≤ 1 g of protein in 24 hours for patient to be eligible.
16. Major surgical procedure, open biopsy or significant traumatic injury within 28 days prior to treatment.
17. Clinically significant cardiovascular disease, for example CVA, myocardial infarction (≤ 12 months before treatment start), unstable angina, NYHA Class II CHF, arrhythmia requiring medication, or uncontrolled hypertension.
18. Evidence of any other disease, metabolic dysfunction, physical examination finding or laboratory finding giving reasonable suspicion of a disease or condition that contraindicates the use of an investigational drug or puts the patient at high risk for treatment-related complications.
19. Known hypersensitivity or contraindication to the drugs used in the trial (eg: aflibercept, 5-FU, folinic acid/ leucovorin, oxaliplatin, bevacizumab, irinotecan).
20. Concomitant treatment with ASS > 325 mg/d or NSAIDs, known to inhibit platelet function, sorivudin or analog compounds or preparations of St. John's wort.
21. Inability or unwillingness to comply with the protocol.

5. Study Procedures and Methodology

5.1 Study Schedule Overview

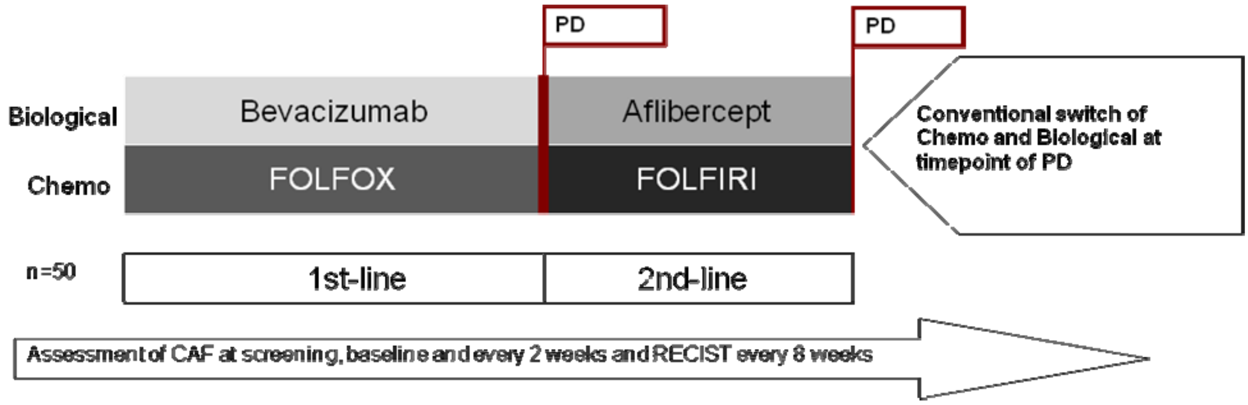

Figure 4: Study schedule overview (run in phase with conventional switch of chemotherapy together with the anti-angiogenic agent)

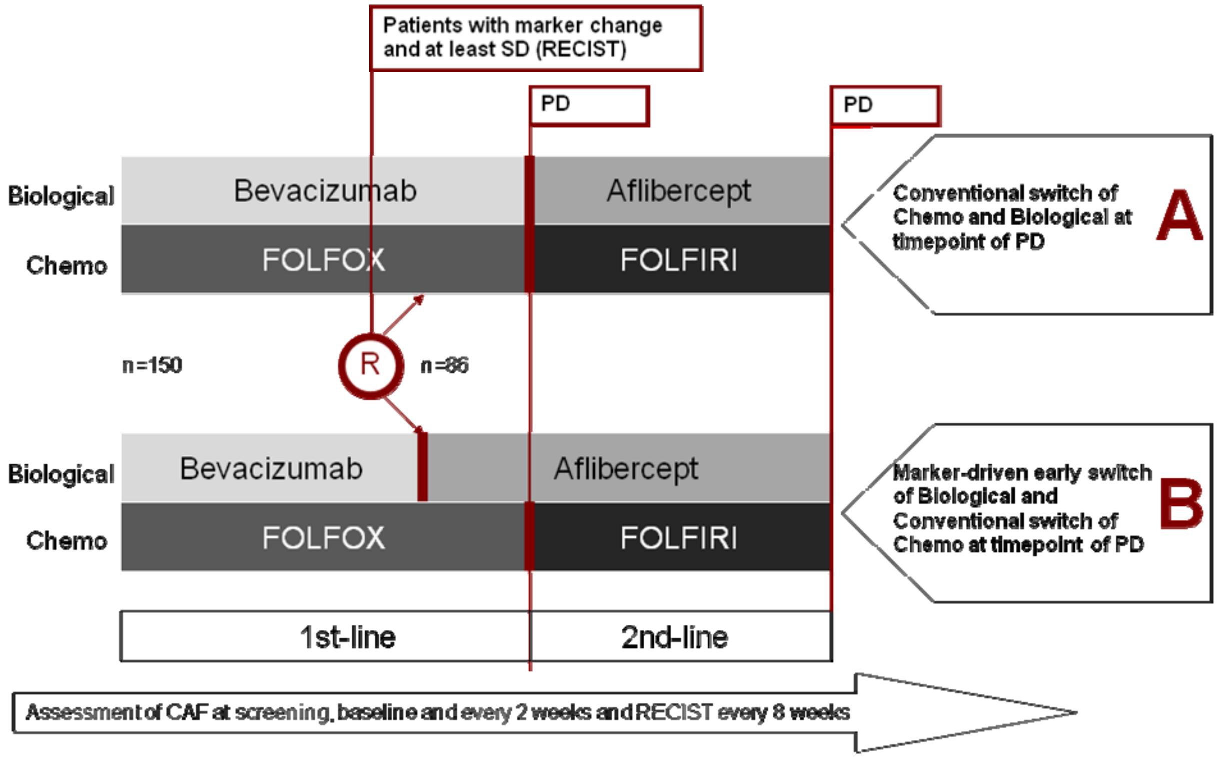

Figure 5: Study schedule overview (randomized part with marker-driven switch of anti-angiogenic agent and maintenance of chemotherapy)

After completing the run in phase of the study, with at least 30 patients completing their first line treatment (due to progression, secondary resection or toxicity) and being evaluable for CAF analyses, the results will be reviewed by an Independent Data Monitoring Comittee (IDMC). Based on that review the decision to continue with, modify or cancel the randomized part will be made. If no CAF profile predictive for radiographic progression can be detected, the randomized study part will not be initiated.

There is no planned accrual suspension up to a recruitment of 100 patients, although recruitment rate will be kept fixed at 10 patients /3 months between the 31st and 51st and 15 patients/3 months between 51st patient and date of available decision of 1st IDMC. Independent of decision of the IDMC about continuation of the trial with the randomized part, inclusion of patients will be continued until the 100th patient (figure 6).

- In case the IDMC decides to continue with the randomized part accrual suspension will be released. After the 50th patient completed first line treatment IDMC will meet again to review the available data and decide about modification, cessation or continuation of the randomized part.
- If the randomized part will not be opened (according to 1st IDMC), recruitment will stop at 100 patients and patients will continue through first and second line with the scheduled treatment plan and marker analysis.

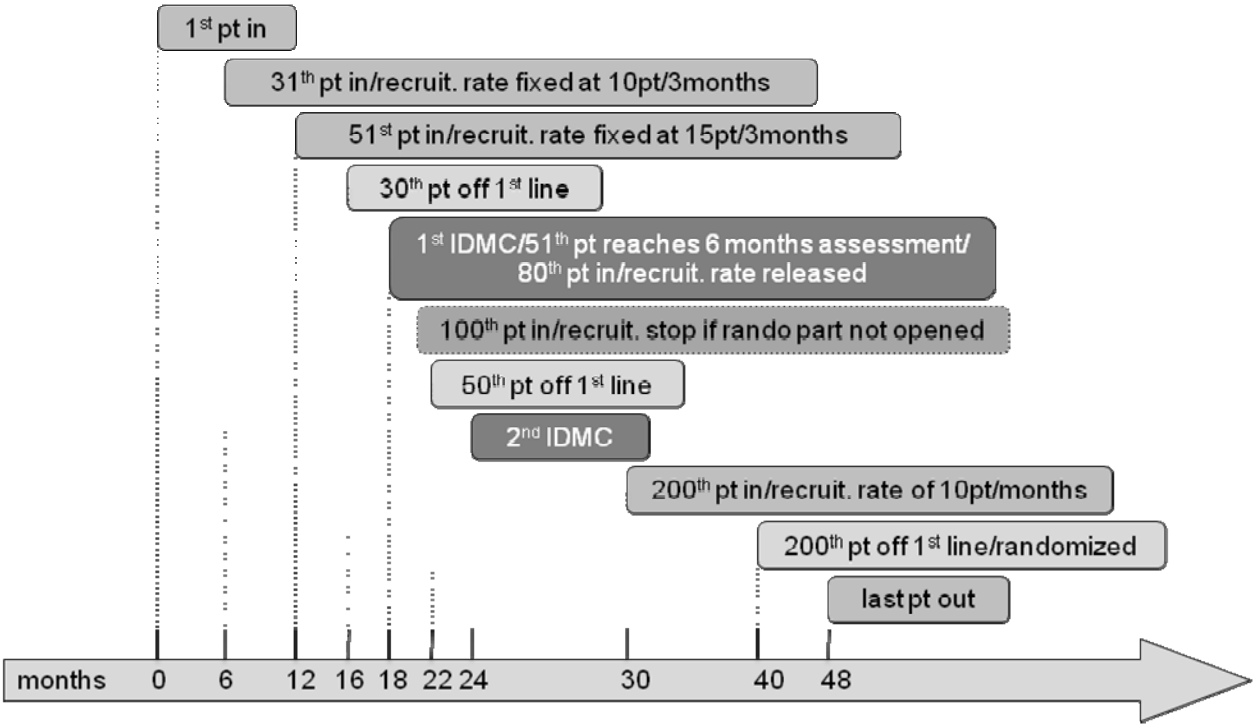

Figure 6: Overview time-points and patient accrual
(abbreviations: pt patient, recruit recruitment, rando randomized, IDMC idependent data monitoring committee)

5.1.1 Treatment

Bevacizumab, 5FU, leucovorin, irinotecan and oxaliplatin are approved for the treatment of metastatic colorectal cancer and will not be supplied or reimbursed. Aflibercept will be supplied and defined as investigational medicinal products (IMP).

5.1.1.1 Treatment schedules

All enrolled patients will be treated 1st line with bevacizumab and mFOLFOX6 every two weeks:

Bevacizumab

bevacizumab 5 mg/kg iv over 30 to 90 min (day 1)

mFOLFOX6

oxaliplatin at a dose of 85 mg/m^2 iv over two hours (day 1)
5-FU 400 mg/m^2 iv bolus (day 1)
LV at a dose of 400 mg/m^2 iv over two hours (day 1)
5-FU at a dose of 2400 mg/m^2 iv over 46 hours (day 1-3)

5.1.2 Treatment duration

Treatment with bevacizumab and mFOLFOX6 will be administered until progression (according to RECIST v1.1), intolerable toxicity or secondary resection in the run in phase. In the marker driven part bevacizumab and mFOLFOX6 will be administered until change of the CAF-profile determined in the run in phase and at least stable disease according to RECIST v1.1, or progression, intolerable toxicity or secondary resection.

5.1.3 Randomization

All eligible patients, being at least stable disease according to RECIST during treatment with mFOLFOX6 and bevacizumab and demonstrating the CAF marker profile to be determined in the run in will be randomized during the treatment cycle following the imaging and central analysis of CAF profile (after 5th/9th/13th or 17th cycle).

For patients not eligible for randomization conventional switch (as in arm A) will be performed.

5.1.3.1 *Arm A (conventional switch of chemotherapy together with the anti-angiogenic treatment)*

Bevacizumab and mFOLFOX6 (continuation of same regimen until progressive disease (PD) according to RECIST v1.1, followed by switch to aflibercept and FOLFIRI after PD).

5.1.3.2 *Arm B (early marker-driven switch of anti-angiogenic agent and maintenance of chemotherapy)*

Aflibercept and mFOLFOX6 (change of bevacizumab to aflibercept and continuation of mFOLFOX6 until PD according to RECIST v1.1, followed by change to FOLFIRI after PD).

All enrolled patients will be treated with aflibercept and FOLFIRI after radiological progression according to RECIST v1.1 (2nd line) every two weeks.

Aflibercept

aflibercept 4mg/kg iv over one hour (day 1)

FOLFIRI

irinotecan 180 mg/m2 iv over one hour (day 1)
5-FU 400 mg/m2 iv bolus (day 1)
LV at a dose of 400 mg/m2 iv over two hours (day 1)
5-FU at a dose of 2400 mg/m2 iv over 46 hours (day 1-3)

5.1.4 Definite change of treatment (second line treatment)

In case of radiological progression at any time point treatment with bevacizumab/aflibercept and mFOLFOX6 will be changed to aflibercept and FOLFIRI (change of chemo-backbone).

5.1.5 Concomitant medication

Antiemetics

For prevention of nausea and vomiting, 5-HT3 antagonists and dexamethasone are strongly recommended for oxaliplatin-based chemotherapy. For delayed nausea and vomiting, the use of oral dexamethasone is recommended; metoclopramide, alizapride, prochlorperazine may be used at the discretion of the prescribing physician. Subjects should have a supply of antiemetics available at home should delayed nausea/vomiting occur.

Antibiotics

In subjects with diarrhoea and neutropenia, even in the absence of fever, empiric use of antibiotics as prophylaxis against bowel sepsis should be strongly considered. Use of a quinolone is suggested in this setting.

Anticoagulants

The use of full dose anticoagulants is allowed as long as the INR or aPTT is within therapeutic limits (according to the medical standard in the institution) and the patient has been on a stable dose for anticoagulants for at least two weeks at the time of registration. During treatment monitoring of INR for oral anticoagulants is recommended.

Antivirals and Antiprotozoals
Fluoropyrimidines should not be administered together with the halogenated antiviral drug sorivudine or its chemically related analogues, such as brivudine. Caution must also be exercised if metronidazole is administered.

Allopurinol
Interactions with allopurinol have been observed for 5-FU; with possible decreased efficacy of 5-FU. Concomitant use of allopurinol with 5-FU should be avoided.

Anti-epileptic Substances
Folinic acid may diminish the effect of anti-epileptic substances: phenobarbital, primidone, phenytoin and succinimides, and may increase the frequency of seizures (a decrease of plasma levels of enzymatic inductor anticonvulsant drugs may be observed because the hepatic metabolism is increased as folates are one of the cofactors).

Growth factors
Haematopoietic growth factors (i.e., G- or GM-CSF) may be used according to institutional guidelines to treat febrile neutropenia, but should not be used as primary or secondary prophylaxis. Growth factors must be discontinued at least 48 hours prior to initiation of the next treatment of chemotherapy.

5.2 Assessments and Guidelines for Visits

		Treatment Period				Follow up Period	
**Study Week (W)**	Screening (day -28 to -7)	every 2 weeks before and after switch of treatment	every 8 weeks before and after switch of treatment	randomization and/or change of treatment	end of treatment (final staging)	every three months (±28 days) until progression or death	after 2nd progression (FOLFIRI+aflibercept) every three months (±28 days) until death
**Informed Consent**	X						
**I/E criteria**	X						
**Medical History/ Demographics**	X						
**Physical Examination[1]**	X	X[8]		X[8]	X[8]	X[8]	
**ECG**	X				X		
**Vital signs[2]**	X	X		X	X	X	
**ECOG PS**	X	X		X	X	X	
**Paraffine embedded tumor tissue**	X						
**Optional fresh biopsy**				X			
**Laboratory determinations[3]**	X	X[11]		X	X	X[12]	
**Serum Pregnancy Test[4]**	X						
**Urine dipstick**	X	X		X	X		
**Tumor markers (CEA, optional CA 19-9)**	X		X		X	X[12]	
**Disease status (RECIST v1.1) [5]**	X		X		if applicable	X	X
**Quality of life ass. [6]**	X		X	X	X		
**Blood draw (7,5ml) for CAF [7]**	X	X[9]		X	X		
**Treatment**		X					
**Concomitant medication/therapy**	X	X		X	X	X	X
**Safety monitoring (NCI CTCAE v4.03)**		X					
**Survival**		X[10]					

1: including weight, (height only baseline), 2: blood pressure, heart rate, respiratory rate, body temperature, 3: hematology: WBC with neutrophils, lymphocytes, monocytes, eosinophils, and basophils, hemoglobin, platelets, hematocrit; blood chemistries: sodium, potassium, calcium, serum creatinine, alkaline phosphatase, AST, ALT, total protein, total bilirubin; albumin, LDH, coagulation (INR, aPTT, PT) baseline and as clinically indicated (e.g. treatment with oral anticoagulants), 4: only women of childbearing potential, 5: documentation of disease status acc. to RECIST v1.1 by MRI or CT scan of thoracic and abdominal region and all other sites of disease (at least one lesion must be identified as target lesion and measured within 4 weeks prior to enrollment) and afterwards every 4 cycles (8 weeks) during treatment, 6: EORTC QLQ-C30 and module CR29, 7: blood draw for CAF will be performed at screening (day -28 to -7) and baseline (d1 just before treatment) every 2 weeks during run-in phase and every 4 weeks during randomized phase, 8: weight, physical examination if clinically indicated, 9: every 2 weeks during run-in phase and every 4 weeks during randomized phase, 10: Follow up for survival might be by telephone call. 11 during treatment only WBC with neutrophils, hemoglobin, platelets, sodium, potassium, calcium, serum creatinine, AST, ALT, total bilirubin, 12: if clinically indicated.

Table 5. Assessments overview

5.2.1 Baseline Assessment

Consenting patients will have the following screening/baseline assessments performed prior to the first treatment.

Within four weeks of the first treatment:

- Relevant medical history and currently ongoing concomitant medication including previous cancer history and cancer treatment will be recorded.
- Physical examination including weight, height and vital signs (blood pressure, heart rate, respiratory rate, body temperature)
- Laboratory tests [WBC with neutrophils, lymphocytes, monocytes, eosinophils, and basophils, hemoglobin, platelets, hematocrit; blood chemistries: sodium, potassium, calcium, serum creatinine, alkaline phosphatase, AST, ALT, total protein, albumin, LDH and total bilirubin; tumor markers: CEA levels and CA19-9 (optional); and coagulation (baseline and as clinically indicated; e.g. treatment with oral anticoagulants): INR, aPTT, PT]
- Urine dipstick
- Demographics
- Performance Status (ECOG) (Appendix B)
- ECG
- Serum pregnancy test (for women of child bearing potential)
- Quality of life assessment using the EORTC QLQ-C30 and the module CR29 (Appendix F)
- Obtain paraffin-embedded tumor-tissue
- Blood draw (7,5ml) for determination of baseline CAF (Blood draw for CAF will be performed at screening (day -28 to -7) and baseline (d1 just before treatment)
- Documentation of disease status
  - All sites of disease should be identified by MRI or CT scan and recorded at baseline. At least one measurable lesion must be identified as target lesion and measured. This scan must be performed within 4 weeks prior to enrollment. Radiological imaging of the chest, abdomen and all other sites of disease have to be performed (CT/MRI-scan of the thoracic and abdominal region).

The investigator will confirm the patient's eligibility after all baseline scans and laboratory results have been reviewed.

5.2.2 Assessments during treatment period

The following assessments will be made every 2 weeks before and after switch of treatment.

- Vital signs (blood pressure, heart rate, respiratory rate, body temperature) and weight
- Performance Status (ECOG) (Appendix B)
- Physical examination (if clinically indicated)
- Laboratory Tests (WBC with neutrophils, hemoglobin, platelets, blood chemistries: sodium, potassium, calcium, serum creatinine, AST, ALT and total bilirubin)
- Blood draw (7.5ml) for CAF analysis (baseline and every 2 weeks during run-in phase and every 4 weeks during randomized phase)

- Urine dipstick
- Adverse Event monitoring and Concomitant Medication/Therapy monitoring

The following assessments will additionally be made every 8 weeks before and after switch of treatment.

- Tumor markers: CEA and CA19-9 (optional)
- Quality of life assessment using the EORTC QLQ-C30 and the module CR29 (Appendix F)
- Documentation of disease status (CT/MRI)

5.2.3 Randomization and/or change of treatment

Blood sampling for CAF will be performed every 4 weeks during randomized phase and sent to central laboratory for immediate analyses every 8 weeks, when disease status (acc. to RECIST v1.1) will be documented. As long as no progressive disease (acc. to RECIST v1.1) will be noted, treatment continues with mFOLFOX6 and bevacizumab. If CAF marker profile suggestive for disease progression (as determined in the run in phase) is noted, respective study site will be contacted to assess current disease status and decide about randomization.

All eligible patients, being at least stable disease according to RECIST during treatment with mFOLFOX6 and bevacizumab and demonstrating the CAF marker profile to be determined in the run in will be randomized during the treatment cycle following the imaging and central analysis of CAF profile (after $5^{th}/9^{th}/13^{th}$ or 17th cycle).

In case of radiological progression (according to RECIST v1.1 as judged by the treating investigator) at any time point treatment with bevacizumab/aflibercept and mFOLFOX6 will be changed to aflibercept and FOLFIRI (change of chemo-backbone).

The following assessment will be made after randomization (day 1 of the $6^{th}/10^{th}/14^{th}$ or 18th cycle) and/or change of treatment, due to radiological progression (day 1of $5^{th}/9^{th}/13^{th}$ or 17th cycle)

- Physical examination (if clinically indicated) and vital signs (blood pressure, heart rate, respiratory rate, body temperature) and weight
- Performance Status (ECOG) (Appendix B)
- Laboratory Tests (red blood cells, WBC with neutrophils, lymphocytes, monocytes, eosinophils, and basophils, hemoglobin, platelets, hematocrit, blood chemistries: sodium, potassium, calcium, serum creatinine, alkaline phosphatase, AST, ALT, total protein and total bilirubin)
- Urine dipstick
- Optional fresh biopsy
- Blood draw (7.5ml) for CAF analysis
- Adverse Event monitoring and Concomitant Medication/Therapy monitoring
- Quality of life assessment using the EORTC QLQ-C30 and the module CR29 (Appendix F)

5.2.4 End of Treatment

The following assessments will be made within 28 days if patient discontinues treatment due to progression after enrollment (e.g. lack of therapeutic efficacy), severe toxicity disabling further treatment continuation, severe adverse events related to the treatment or any of the points mentioned in 5.5.1.

- Physical examination including vital signs (blood pressure, heart rate, respiratory rate, body temperature) and weight
- Performance Status (ECOG) (Appendix B)
- Laboratory Tests (red blood cells, WBC with neutrophils, lymphocytes, monocytes, eosinophils, and basophils, hemoglobin, platelets, hematocrit; blood chemistries: sodium, potassium, calcium, serum creatinine, alkaline phosphatase AST, ALT, total protein and total bilirubin; tumor markers)
- Blood draw (7.5ml) for CAF analysis
- Urine dipstick
- Adverse Event monitoring and Concomitant Medication/Therapy monitoring
- ECG
- Quality of life assessment using the EORTC QLQ-C30 and the module CR29 (Appendix F)
- If applicable: documentation of disease status (CT/MRI) and CEA (CA 19-9 optional)

5.2.5 Follow-up Period

Patients will be followed-up every 3 months ± 28 days until progression or death up to a maximum of 5 years per individual patient after trial inclusion for:

- Safety evaluation using NCI CTCAE v4.03 and Concomitant Medication/Therapy monitoring
- Physical examination, including vital signs (blood pressure, heart rate, respiratory rate)
- Performance Status (ECOG) (Appendix B)
- Laboratory determinations, if clinically indicated (CBC, blood chemistries, tumor markers)
- Disease status assessment: follow-up scan every three months and any other time point during the conduct of the study if there is an indication for a change in disease status: same lesions and method as at study entry.

After 2nd progression (with FOLFIRI+Aflibercept), patients will be followed every 3 months ± 28 days until death. The investigator will record disease status, protracted toxicities, further treatment and patient survival.

5.3 Study Duration

Study duration is planned as follows:

First patient in:	QII 2014
Last patient in: (recruitment 30 months)	QIV 2016
Last patient out/study completion:	QII 2018
Follow up for overall survival until:	QII 2020

Maximum time on trial per patient is 5 years.

5.4 Rules for contraception and pregnancy reporting

A negative pregnancy test and willingness to use highly effective methods of contraception (per institutional standard) during treatment and for 6 months (male or female) after the end of treatment is absolutely essential for subjects to participate in the

PERMAD-trial (adequate: intrauterine device, long-acting injection, hormon implant, vasectomy).

Any pregnancy in a female subject or in a female partner of a male subject diagnosed during the treatment period or within 6 months after last study treatment administration must be reported unhesitatingly to the CRO. Follow-up information on the subject and her pregnancy outcome should be communicated by the Investigator to the CRO as soon as available.

5.5 Study Termination

5.5.1 Patient Withdrawal

Patients may be withdrawn from the study by the investigator or terminate their participation prematurely based on the following:

- Post-consent determination of ineligibility based on safety or eligibility criteria
- Lack of therapeutic efficacy, as evidenced by progression
- Interval down staging of target lesions such that the patient would be an acceptable candidate for curative treatments
- Physician’s judgment following an adverse event
- Termination by the Sponsor, or a regulatory authority
- Patients that require radiation therapy for local palliative purposes
- Any other reason for withdrawal that the study physician or patient indicates is in the overall best interest of the patient

All patients will be followed by clinical visitations or telephone contact post withdrawal for assessment of overall survival.

Patients who withdraw consent prior to receiving any therapy will be withdrawn from the study and no follow-up safety surveillance is required.

Patients who voluntarily withdraw consent or who are withdrawn by the study physician for any reason after receiving therapy will be followed-up for at least 7 days. The purpose of this follow-up is to capture all adverse events and document any serious, procedure related adverse events.

If a patient dies prior to the last scheduled study visit, the date and cause of death will be recorded.

5.5.2 Study Completion

The study will be considered complete when:

- all patients have exited the study following progression during or after second line, or
- all patients have discontinued treatment.

Overall survival post study completion will be assessed by clinical visit and/or telephone follow-up for a maximum of two years after last patient out.

6. Investigational Medical Product: Aflibercept

Aflibercept (ZALTRAP^{TM}) is a monoclonal antibody inhibiting VEGF and PIGF.
Further details are available in the investigator’s brochure.

6.1 Structure, molecular mass

Aflibercept (VEGF Trap) is a recombinant protein consisting of sequences derived from human VEGF receptor extracellular domains fused to the Fc portion of human IgG1. The extracellular domain sequences come from 2 different VEGF receptors, VEGFR1 (also known as Flt-1) and VEGFR2 (also known as KDR or Flk-1). Each of the VEGF receptors are composed of seven immunoglobulin (Ig) domains in their extracellular regions, with Ig domains 2 and 3 contributing the majority of the binding energy for VEGF. Thus the amino acid sequence structure of aflibercept comprises Ig domain 2 from VEGFR1, fused to Ig domain 3 from VEGFR2, which is in turn fused to the Fc domain of IgG1. There are no extraneous linker sequences between any of the peptide domains. The presumptive Ig domain structure of aflibercept is provided in Figure 7.

Aflibercept contains ~15% glycosylation yielding a total molecular weight of 115 kDa (based on multiangle laser light scattering studies).

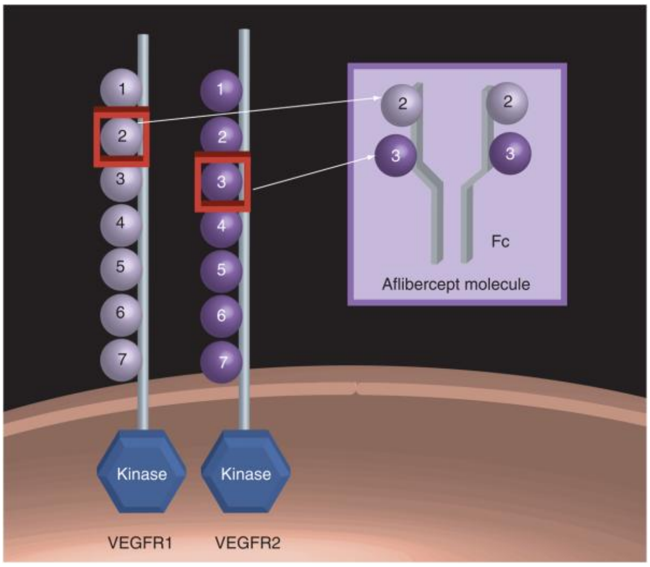

Figure 7: Aflibercept/VEGF$_{R1R2}$ structure

Domain 2 of VEGFR1 and domain 3 of VEGFR2 complexed with the Fc portion of human IgG1.

Aflibercept is a covalent (disulfide linked) dimer of identical subunits. Based on reduced and non-reduced SDS PAGE, the observed molecular weights are approximately 48 000 daltons and 98 000 daltons for the de-glycosylated monomer and dimer, respectively. The values for the glycosylated forms are 58 000 and 115 000 daltons.

All five putative N-glycosylation sites predicted by the primary sequence (two in R1 Ig domain 2, two in R2 Ig domain 3, one in Fc domain) can be occupied with carbohydrate and exhibit some degree of chain heterogeneity, including heterogeneity in terminal sialic acid residues, except at the single unsialylated site associated with the Fc region. The disulfide bond structure of aflibercept was determined using standard peptide mapping methods and matches that predicted based on the known disulfide patterns of the VEGFR1 (Ig domain 2), VEGFR2 (Ig domain 3) and the IgG Fc domain. There are 4 intramolecular disulfide bonds/monomer (one in R1 Ig domain 2, one in R2 Ig domain 3, two in Fc domain) and two intermolecular disulfides between Fc domains. The N-terminal amino acid sequence of the recombinant protein (SDTGRPFVEMYSEIP) corresponds to the sequence predicted by the clone following removal of the signal sequence. However, like many recombinant antibodies, the carboxyl terminus lacks the predicted C-terminal

lysine residue on the Fc moiety. As a recombinant protein, aflibercept is iosynthesized from l-amino acids. Aflibercept contains 431 amino acids.

Mechanism of action

Aflibercept is a specific antagonist that binds and inactivates circulating VEGF. Aflibercept was designed to prevent the growth of primary and metastatic tumors by blocking tumor angiogenesis and vascular permeability.

Nomenclature

- Laboratory code number: AVE0005
- INN: Aflibercept
- Synonymous Name: VEGF Trap

Description and list of excipients

Concentrate for solution for IV infusion.

Aflibercept is supplied for IV administration as a sterile, non-pyrogenic, colorless to pale-yellow colored, 25 mg/mL solution, packaged in a type 1, clear borosilicate glass vial closed with a flanged cap with tear-off lid and inserted sealing disc, Flurotec® (PTFE) coated. The pH of the solution is about 6.2. The aqueous solution contains the following excipients: sucrose, sodium chloride, sodium citrate dihydrate, citric acid monohydrate, polysorbate 20, sodium phosphate dibasic heptahydrate, sodium phosphate monobasic monohydrate, and water for injection.

Within this trial the following aflibercept drug product presentation is available:

- 200 mg/8 mL in a 10 mL vial

The fill volume has been established to ensure removal of 8 mL, respectively. Prior to infusion, the aflibercept dosage form must be diluted directly into infusion bags of 0.9% sodium chloride solution or 5% dextrose. The concentration of the diluted solution can range between 0.6 and 8 mg/mL. The pH of the diluted solution is about 6.2. Aflibercept does not contain any microbial preservative. Therefore, care must be taken to ensure the sterility of the prepared solution. The dilution must be carried out by a healthcare professional under aseptic conditions.

Adventitious agents safety evaluation

Aflibercept drug product complies with bovine spongiform encephalopathy/transmissible spongiform encephalopathy (BSE/TSE) and viral safety regulations.

6.2 Preparation and administration of aflibercept

6.2.1 Preparation

Multiple vials of aflibercept concentrate for solution for infusion may be required depending on the patient's weight and the intended dose. The necessary volume of aflibercept should be withdrawn from the vials and injected directly into the infusion bag. Aflibercept concentrate should be diluted for infusion with 0.9% sodium chloride solution or 5% dextrose by a healthcare professional. The dilution must be carried out under aseptic conditions. Any unused portion left in a vial must be discarded, as the investigational drug product does not contain any preservatives.

Infusion bags made of the following materials may be used:

- PVC containing DEHP
- Polyolefin (PVC free DEHP free)

The final concentration of the diluted solution can range between 0.6 mg/ml and 8 mg/ml. This concentration allows for:

- 44 to 497 mg of aflibercept concentrate to be diluted in a 50 mL PVC – DEHP bag
- 43 to 536 mg of aflibercept concentrate to be diluted in a 50 mL polyolefin bag
- 79 to 980 mg of aflibercept concentrate to be diluted in a 100 mL PVC – DEHP bag
- 80 to 1061 mg of aflibercept concentrate to be diluted in a 100 mL polyolefin bag
- 120 to 1494 mg of aflibercept concentrate to be diluted in a 150 mL PVC – DEHP bag
- 196 to 1950 mg of aflibercept concentrate to be diluted in a 250 mL PVC – DEHP bag

Polypropylene syringes may be used. Chemical and physical stability of the diluted aflibercept solutions in polypropylene syringe or infusion bags has been demonstrated for up to 24-hours under refrigerated conditions (2° to 8°C) or for up to 8 hours at room temperature (approximately 25°C).

From a microbiological point of view, the product should be used immediately. In-use storage times and conditions are the responsibility of the user. Dilution under aseptic conditions should be applied.

Diluted solutions of aflibercept should be administered using infusion tubing made of the following materials:

- PVC containing DEHP
- DEHP-free PVC containing TOTM
- polypropylene
- polyethylene lined PVC
- polyurethane

6.2.2 Infusion conditions

The infusion sets must contain a 0.2 µm polyethersulfone inline filter. PVDF or Nylon filters should not be used. Infusion can be conducted by gravity, with an IV infusion pump, or with a syringe pump using administration sets made of the above materials. The aflibercept IV dose should be infused over 1 hour. The infusion should not exceed 2 hours at ambient temperature (approximately 25°C). Parenteral investigational drug products must be inspected visually for particulate matter and discoloration prior to administration.

6.2.3 Storage period of premix and infusion solution

The aflibercept concentrate for solution for infusion in its original unopened container is stable for 36 months under refrigerated conditions (2 to 8°C).

The Hospital Pharmacist will be responsible for the appropriate storage of the IMP at the study centre, and must immediately inform the Monitor/Sponsor of non-respect of the required storage conditions. When closing the investigational centre, all unused IMP

containers will be destroyed on site. If an IMP batch is suspected to be defective, Sanofi-Aventis will immediately inform the Sponsor so that the Hospital Pharmacist can immediately get the appropriate information. The Hospital Pharmacist will organise the destruction of the concerned batch(es) and new batch(es) will be sent to the investigational centre when appropriate.

6.2.4 Storage conditions and shelf life, destruction of used IMP

The aflibercept concentrate for solution for infusion in its original unopened container is stable for 36 months under refrigerated conditions (2 to 8°C).

All used IMP vials and packages should be kept in the Pharmacy until drug accountability has been performed by the responsible monitor. After approval of the sponsor used IMP can be destroyed on site according to national specifications, adequate documentation should be filed in the Pharmacy file / Investigator Site File of the study.

7. Dose modifications

7.1 Bevacizumab

No dose reduction of bevacizumab is foreseen for an individual patient. Skipped doses or termination of treatment will be based on the observed toxicities as specified below. No dose adjustments are allowed except for body weight changes of more than 10%. Missed doses will not be made up for. A rounding up or down of the dose is acceptable to allow practical ease of administration (±10%).

Specific instructions on the grading and management of hypertension, proteinuria, thrombosis/embolism and haemorrhage and other events attributable to bevacizumab, are provided below.

Grade 3-4 bevacizumab-related AEs, should be managed as below:

- First occurrence: hold bevacizumab until toxicity has improved to grade ≤1
- Second occurrence: permanently discontinue treatment

In addition any patient who experiences the following events should permanently discontinue bevacizumab:

- Gastrointestinal perforation, abscesses and fistulae
- Arterial or venous thromboembolic events
- Grade 3/4 haemorrhagic events other than pulmonary or CNS haemorrhage
- Symptomatic grade 4 thrombosis appropriately manageable by medication
- Grade 4 proteinuria (nephrotic syndrome)
- Reversible posterior leucoencephalopathy syndrome
- Any grade of pulmonary haemorrhage/haemoptysis
- Any grade CNS haemorrhage
- Any grade hypersensitivity/allergic reactions in response to bevacizumab infusion
- Grade ≥3 bowel obstruction
- Any grade of wound dehiscence requiring medical or surgical intervention (if the wound is from an incision that entered a body cavity)
- Grade ≥3 left ventricular systolic dysfunction

Note that bevacizumab should be temporarily interrupted in the event of febrile grade 4 neutropenia and/or grade 4 thrombocytopenia, since these conditions are predisposing factors for an increased bleeding tendency. In case bevacizumab is discontinued, chemotherapy should be continued.

7.1.1 Surgical procedures / wound healing complications

Bevacizumab therapy should not be initiated for at least 28 days following major surgery or until the surgical wound is fully healed. In patients who experience wound healing complications during bevacizumab treatment, bevacizumab should be withheld until the wound is fully healed.

Bevacizumab should be withheld for at least 5 weeks before conducting elective surgery. Emergency surgery should be performed as appropriate without delay.

7.1.2 Hypertension

Blood pressure needs to be assessed before each bevacizumab administration.

The following table lists the clinical action to be taken in the event of an AE/SAE of hypertension.

Blood pressure measurements should occur after the patient has been in a resting position for ≥5 minutes. Repeat measurement of BP for verification should be undertaken if the initial reading is ≥140 mmHg systolic and/or ≥90 mmHg diastolic pressures.

CTC AE Grade of Hypertension	Description	Action
1	Asymptomatic, transient (< 24 hrs) increase by > 20 mmHg (diastolic) or to > 150/100 mmHg if previously within normal limits	Intervention not indicated
2	Recurrent or persistent (> 24 hr) or symptomatic increase by > 20 mmHg (diastolic) or to > 150/100 mmHg if previously within normal limits	Monotherapy of anti-hypertensive may be indicated. Once controlled to < 150/100 mmHg, patients may continue bevacizumab therapy
3	Requiring more than one anti-hypertensive or more intensive therapy than previously	Bevacizumab should be withheld for persistent or symptomatic hypertension and should be permanently discontinued if hypertension is not controlled
4	Life threatening consequence (e.g. hypertensive crisis)	Occurrence of grade 4 hypertension should lead to permanent discontinuation of bevacizumab

Table 6. Management of Hypertension

All doses of anti-hypertensive medicines should be recorded at all visits.

7.1.3 Proteinuria

Patients with a history of hypertension may be at increased risk for the development of proteinuria when treated with bevacizumab. There is evidence suggesting that Grade 1 proteinuria may be related to bevacizumab dose. Monitoring of proteinuria by dipstick urinanalysis is recommended prior to starting and during bevacizumab therapy. Bevacizumab should be discontinued in patients who develop Grade 4 proteinuria (nephrotic syndrome).

All patients receiving bevacizumab will have an urinanalysis performed within 48 hours prior to each bevacizumab dose. All toxicity will be graded according to NCI CTC-AE (version 4.03) guidelines. Adjustment of bevacizumab administration for proteinuria will occur according to the following guidelines:

- Proteinuria by dipstick has to be assessed before each bevacizumab administration unless proteinuria has been determined by 24-hour urine

7.1.4 First occurrence of proteinuria during treatment with bevacizumab

- **<2+** proteinuria (dipstick): administer bevacizumab as planned
- **≥2+** proteinuria (dipstick): administer bevacizumab as planned and collect 24-hour urine for determination of total protein within 3 days before the next scheduled bevacizumab administration:
  - **If 24-hour proteinuria ≤2 g**: administer next bevacizumab dose as scheduled
  - **If 24-hour proteinuria >2 g**: omit next scheduled bevacizumab dose and do 24-hour urine collection for determination of total protein within 3 days before the subsequently scheduled cycle. Delay bevacizumab treatment until proteinuria has decreased to ≤2 g. Do 24-hour urine before each scheduled dose until proteinuria has improved to ≤1 g/24 hours, but omit bevacizumab only if >2 g/24 hours
- An algorithm of bevacizumab interruption due to proteinuria is given in Figure 7 and 8.
- Nephrotic syndrome (Grade 4, NCI CTC-AE v4.03): Permanently discontinue bevacizumab treatment

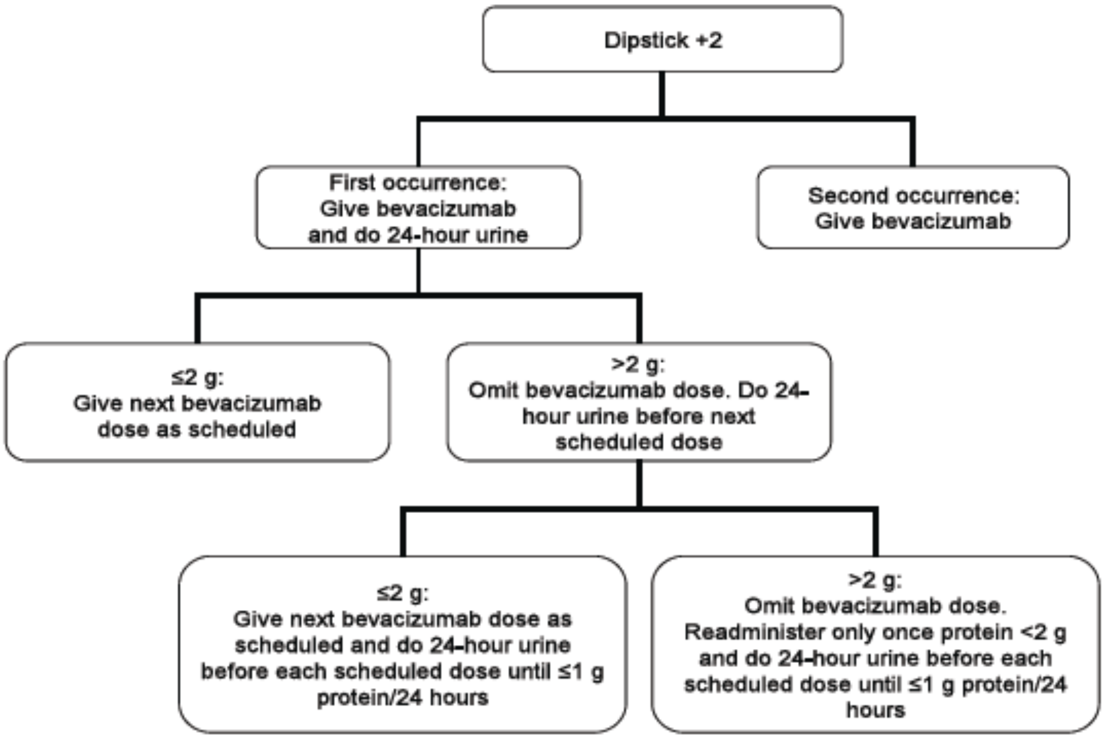

Figure 8: Algorithm of Bevacizumab Dose Interruption in Case of Dipstick 2+ Proteinuria

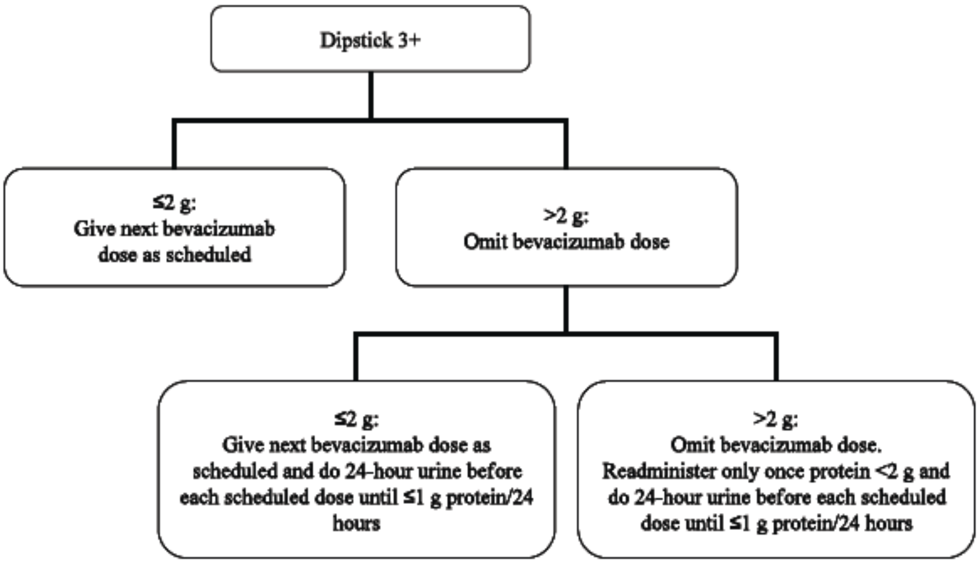

Figure 9: Algorithm of Bevacizumab Dose Interruption in Case of Dipstick ≥ 3+ Proteinuria

7.1.5 Thrombosis / embolism

For patients who develop grade 3 or 4 thrombosis/embolism the following action is recommended:

- Bevacizumab should be permanently discontinued in patients who develop arterial thromboembolic events or symptomatic grade 4 thromboembolism.
- Grade 3 or 4 venous thrombosis: Hold study drug treatment for 2 weeks. Bevacizumab may be resumed after initiation of therapeutic-dose anticoagulant therapy as soon as all of the following criteria are met:
  - The patient must be on a stable dose of anticoagulant and, if on warfarin, have an INR within the target range prior to restarting study drug treatment
  - The patient must not have had a grade 3 or 4 haemorrhagic event since entering the study
  - The patient must not have had any evidence of tumor invading or abutting major blood vessels on any prior disease assessment.

7.1.6 Haemorrhage

All toxicity will be graded according to NCI CTC-AE guidelines (version 4.03). Dose modification of bevacizumab in patients who develop Grade 3 or 4 toxicity for haemorrhage attributable to bevacizumab is as follows:

Patients who develop grade 3 or 4 haemorrhage should discontinue bevacizumab treatment.

7.1.7 Congestive Heart Failure

Prior anthracyclines exposure and/or prior radiation to the chest wall may be possible risk factors for the development of CHF. Caution should be exercised before initiating bevacizumab therapy in patients with these risk factors. No significant increased incidence of CHF in patients treated with bevacizumab was observed in NSCLC or CRC trials. Bevacizumab should be permanently discontinued in Grade ≥3 left ventricular systolic dysfunction.

7.1.8 Tracheo-oesophageal fistula

Patients should be monitored closely on an ongoing basis for emergent oesophagitis, oesophageal pain and/or dysphagia. Treatment with bevacizumab should be interrupted if patients develop severe or persistent oesophagitis and should not be re-introduced until the oesophagitis recovers to at least CTC Grade 1 and after discussion with the study medical monitor.

Patients receiving concurrent chemotherapy and radiation plus bevacizumab for the treatment of disease including the trachea, bronchi and/or oesophagus structures may be at particular risk for the development of tracheo-oesophageal fistula. For patients who are to receive non-urgent palliative radiotherapy to the mediastinal region, it is recommended to allow at least six weeks between the administration of the last dose of bevacizumab and the start of radiotherapy. Investigators should assess and consider the benefit of palliative radiotherapy versus the risk of the potential development of tracheo-oesophageal fistula.

Bevacizumab should be permanently discontinued in patients with TE fistula or any grade 4 fistulae. Limited information is available on the continued use of bevacizumab in patients with other fistulae. In cases of internal fistula not arising in the GI tract (< grade 4), discontinuation of bevacizumab should be considered.

7.1.9 Hypersensitivity reactions/infusion reactions

In some clinical trials anaphylactic and anaphylactoid-type reactions were reported more frequently in patients receiving bevacizumab in combination with chemotherapy than with chemotherapy alone. The incidence of these reactions in some clinical trials of bevacizumab is common (up to 5% in bevacizumab-treated patients). For patients with any grade hypersensitivity reactions/infusion reactions bevacizumab infusion should be discontinued.

7.2 Aflibercept

Aflibercept dose reduction is described in Table 7. Only one dose reduction is allowed.

Sponsor should be contacted by Investigator for discussion on a case by case basis if deemed appropriate, upon clinical judgment.

	**Initial dose (mg/kg)**	**Dose reduction, level – 1 (mg/kg)**
Aflibercept	4	2

Table 7. Aflibercept dose reduction level

Actions considered for aflibercept according to the type of toxicity, are described in Table 8 and 9.

Toxicity	Grade	Action
**Hypertension**	Grade ≤ 2<br><br><br><br><br>Grade 3 (requiring more than one drug or more intensive therapy than previously)<br><br><br><br><br><br><br><br><br><br><br><br><br><br><br><br><br><br>Grade 4	Initiate antihypertensive drug therapy (see recommendation below) and close monitoring of blood pressure for further adjustment, as needed.<br>No dose modification and no delay.<br>Modify antihypertensive drug therapy (see recommendation below).<br><br>Delay the administration of both FOLFIRI/mFOLFOX6 **and** aflibercept for a maximum of 2 weeks, until recovery to blood pressure (BP) ≤ 140/90 or to systolic BP < 160 if diastolic BP < 90 for patients with known history of isolated systolic hypertension:<br>• If BP is controlled within 2 weeks delay:<br>- First episode: re-administer FOLFIRI/mFOLFOX6 **and** aflibercept at the same dose.<br>- Second episode: re-administer FOLFIRI/mFOLFOX6 **and** aflibercept, with aflibercept reduced to dose level -1.<br>-Third episode, discontinue aflibercept.<br>• If BP is still uncontrolled despite appropriate anti hypertensive treatment and after 2 weeks delay: Administer FOLFIRI/mFOLFOX6 **and** discontinue aflibercept for 1 cycle; the reintroduction of aflibercept at a dose reduced to dose level – 1 will be reconsidered at the time of the administration of the subsequent cycle (in combination with FOLFIRI), only if BP is controlled at the time of re-administration.<br>• In case of re-occurrence of grade 3 BP, in presence of maximal/optimal antihypertensive therapy despite dose reduction of aflibercept, or if BP is still uncontrolled despite 1 omission of administration of aflibercept, the patients will be permanently discontinued from aflibercept. FOLFIRI/mFOLFOX6 will be continued if the investigator thinks the patient is benefiting from it.<br><br>Seek cardiologist opinion, and permanently discontinue aflibercept.
**Arterial thromboembolic events** (e.g.: myocardial infarction, or stroke, etc) **documented by appropriate tests**	Any Grade	Permanently discontinue aflibercept.
**Hemorrhage**	Grade 3-4	Permanently discontinue aflibercept.
**GI perforation/ fistula formation**	Any Grade	Permanently discontinue treatment.

Toxicity	Grade	Action
**Reversible posterior Leuko-encephalopathy syndrome documented with appropriate tests**	Any grade	Permanently discontinue treatment.
**Venous Thromboembolic Event documented by appropriate tests**	Grade 3 (DVT)	First episode: Treat DVT with heparins **and** continue treatment [a]<br>Second episode despite appropriate anticoagulation: Permanently discontinue aflibercept
	Grade 4 (PE)	Permanently discontinue aflibercept [b]

[a] Based on investigator's judgement in assessing potential risk of extension and/or embolization.

[b] Continuation of aflibercept may be considered, depending on individual patient benefit/risk assessment in case of incidental discovery of asymptomatic pulmonary embolism.

Table 8. Dose modifications for aflibercept

7.2.1 Hypertension therapy recommendations:

- *For patients without prior antihypertensive therapy*, at the time of the hypertensive episode a close monitoring of the BP (every 2 weeks is recommended) should be initiated for adjustment in treatment, as needed. Ultimately, antihypertensive treatment must be individualized based on the presence of comorbidity factors such as diabetes, cardiovascular or renal disease, additionally taking into account the safety and the efficacy of any prior antihypertensive therapy received. Oral and/or intravenous sodium intake should be carefully monitored in these patients.
- *For patients already under anti-hypertensive therapy*, efforts should be done to optimize the existing therapy before adding other agents as required to control the BP.

When hypertension is accompanied by signs or symptoms of end organ damage such as hypertensive retinopathy, kidney function abnormalities (like progressive proteinuria), or any signs or symptoms of cardiovascular morbidity or central nervous system (CNS) morbidity, aflibercept should be interrupted.

7.2.2 Proteinuria

Determination and management of proteinuria:
Prior to each administration of aflibercept perform a urine dipstick for WBC, RBC and protein (morning spot urine).
If proteinuria is < 2+ on the dipstick in absence of hematuria, aflibercept should be administered as planned.
If the proteinuria is $\geq$ 2+ on the dipstick, UPCR (Urinary Protein/urinary Creatinine Ratio) should be performed prior to administration of aflibercept.

Urinary protein creatinine ratio (UPCR) corresponds to the ratio of urinary protein and urinary creatinine concentrations (expressed in mg/dL). There is a high correlation between morning UPCR and 24-hour proteinuria in patients with normal or reduced renal

function. This ratio provides an accurate quantification of 24-hour urinary protein excretion.

UPCR to detect proteinuria, should be done on morning urine spot. If UPCR > 1, 24-hour urine collection to grade proteinuria will be performed. In addition, in case UPCR is greater than 2 or in case of proteinuria of renal origin (according to urinary protein electrophoresis and/or nephrologist judgement) is associated with hematuria (microscopic or macroscopic), then a work up for considering thrombotic microangiopathy should be initiated and a nephrologist consultation be considered.

Delay in availability of the results should not delay consultation with a nephrologist.

Actions with regard to aflibercept dosing will depend on the presence of hematuria and the level of 24-hour proteinuria results. Only one dose level reduction for aflibercept is foreseen.

Aflibercept administration should be suspended for ≥2 grams of proteinuria/24 hours and restarted when proteinuria is <2 grams/24 hours. If there is recurrence, the administration should be suspended until <2 grams/24 hours and then the dose should be reduced to 2 mg/kg.

Aflibercept treatment should be discontinued in patients who develop nephrotic syndrome or TMA.

Proteinuria should always be assessed taking into account the presence or absence of hematuria and the blood pressure status of the patient.

7.2.3 Reversible posterior leuko-encephalopathy (RPLS) or clinical symptoms related to vasogenic edema of the white matter.

Clinical presentations are variable and may include headache, altered mental status, seizure and cortical visual deficit. Hypertension is a risk factor. MRI scans are key to diagnosis and typically demonstrate vasogenic edema (hyperintensity in T2 and FLAIR images and hypodensity in T1 images) predominently in the white matter of the posterior parietal and occipital lobes; less frequently, the anterior distributions and the gray matter may also be involved. RPLS should be in the differential diagnosis in patients presenting with unexplained mental status change, visual disturbance, seizure, or other CNS findings. RPLS is potentially reversible with early recognition of symptoms and timely correction of the underlying causes, including control of BP and interruption of the offending drug, which are important in order to prevent progression to irreversible tissue damage.

7.2.4 Gastro-intestinal perforation:

Patients should be monitored for signs and symptoms of GI perforation as this has been reported with anti-VEGF agents as a class.

7.2.5 Hypersensitivity reaction:

In case of hypersensitivity reaction, institutional treatment guidelines for this type of AEs, or the following proposed guideline in Table 9 can be applied. Pre-treatment with corticosteroids and/or antihistamines may be considered in subsequent cycles.

Symptom Severity	Intervention Recommendation
Mild-Moderate<br>e.g., NCI CTCAE grade ≤ 2 cutaneous reaction, pruritus, flushing, rash, dyspnea, tachycardia, hypotension, anxiety, headache, myalgias, edema, nausea	Stop aflibercept infusion;<br>Give diphenhydramine 50 mg IV and/or IV dexamethasone 10 mg;<br>Resume aflibercept infusion after subject recovery.
Severe<br>e.g., symptomatic bronchospasm, generalized urticaria, systolic BP ≤ 80 mm Hg, angioedema, anaphylaxis	Stop aflibercept infusion;<br>Give IV diphenhydramine 50 mg and/or IV dexamethasone 10 mg and/or epinephrine as needed;<br>Permanently discontinue aflibercept.

Table 9. Acute infusion reaction management

7.2.6 Wound healing complications/surgery

The half-life of bound aflibercept is approximately 20 days. Suspend aflibercept for at least 4 weeks prior to elective surgery.
Aflibercept therapy should not be administered for at least 4 weeks following major surgery and not until the surgical wound is fully healed.
For minor surgery such as central venous access port placement, biopsy and tooth extraction, aflibercept may be initiated/restarted once the surgical wound is fully healed.
Aflibercept should be permanently discontinued in patients who develop wound dehiscence or compromised wound healing requiring medical intervention.

7.3 Dose modifications for chemotherapy

Systematic toxicity evaluation using the National Cancer Institute Common Toxicity Criteria (NCI-CTC) Version 4.03 (see Appendix C) begins with the first treatment cycle, and is repeated before each further cycle and at the end of treatment.

Local standard practice will drive dose reduction or schedule modifications of chemotherapeutic regimens. In case of toxicity related chemotherapy dose reduction no dose reescalation is allowed.

Additional guidelines are given in tables 4 and 5. In case a patient experiences severe chemotherapy-related toxicity or progressive disease, Investigators will be allowed to modify or change the chemotherapy regimen as appropriate.

7.3.1 General notes regarding dose modifications for chemotherapy-related toxicity

- For adverse events which are considered by the Investigator unlikely to develop into serious or life-threatening events and which do not result in a delay or interruption of therapy (e.g. alopecia, altered taste etc.), treatment will be continued at the same dose without reduction or interruption.
- If toxicity requires a dosing delay or interruption of any drug for more than one cycle, that chemotherapeutic drug should be discontinued. The patient can remain on study with the remaining chemotherapeutic drugs and will continue to be evaluated according to study procedures (Monotherapy with oxaliplatin is excluded).
- If all chemotherapy must be discontinued permanently due to related toxicity, the patient may continue with bevacizumab.

- If chemotherapy is withheld due to toxicity, bevacizumab might also be withheld in order to maintain the synchronisation of both treatment regimens. However, treatment with bevacizumab may continue at the Investigator's discretion if no specific bevacizumab-related toxicity is present (see Section 6.1).

7.3.2 Guidelines for dose modifications

Patients developing toxicity will be managed according to table 4. Administration of 5-FU/LV should be delayed in case of toxicity.

The occurrence of grade 4 non-haematological toxicity other than nausea/vomiting or alopecia or grade 4 thrombocytopenia necessitates withdrawal from study treatment.

7.3.3 Toxicity at the start of the following cycle

Patients must meet the following criteria before each new cycle:

- Absolute neutrophil count ≥ 1500/mm^3 and platelet count ≥ 100.000/mm^3
- Treatment-related diarrhea and/or abdominal cramps are fully resolved to baseline or grade 0 and no loperamide has been administered during the last 24 hours.
- Any bilirubin elevation has resolved to at least grade 1.
- Recovery from any treatment-related grade 3/4 non-hematological toxicity (except alopecia) to baseline or ≤ grade 1.

Patients not meeting the above criteria on the date scheduled for the new cycle must suspend treatment with the anticancer drugs 5-FU/leucovorin, irinotecan or oxaliplatin until they meet the above criteria.

Bevacizumab is continued without dose reduction except in the case of bevacizumab-related toxicity (see “dose modification: bevacizumab”).

Dose adjustments are at the investigator’s discretion insofar as they must take account of the patient’s clinical situation and the suspected causal relationship between the toxicities and administration of the anticancer drugs. Dose re-escalation is not permitted. If the above criteria necessitate postponement for more than 3 weeks, the patient should be withdrawn from the study.

**Adverse Event**	**Grade**	**Dose modification**
**Neutropenia**	Grade ≥ 3 or grade 4 for < 5 days	Delay/interrupt chemotherapy until resolution to grade ≤ 2<br>Consider use of G-CSF<br>*3rd occurrence requiring dose delay within study:*<br>Reduce all further doses of oxaliplatin, irinotecan, 5-FU and LV to 75%
	Grade 4 for ≥ 5 days or Febrile neutropenia	Reduce all further doses of oxaliplatin, irinotecan, 5-FU and LV to 75%
**Thrombopenia**	Grade ≥ 2	Delay/interrupt chemotherapy until resolution to grade ≤ 1
	Grade ≥ 3	Reduce all further doses of oxaliplatin, irinotecan and 5-FU and LV to 75%
	Grade 4	Discontinue treatment
**Diarrhea**	Grade ≥ 2	Delay/interrupt chemotherapy until diarrhea grade 0 or baseline<br>*2nd occurrence requiring dose delays:*<br>Delay/interrupt chemotherapy until diarrhea grade 0 or baseline and reduce irinotecan, 5-FU and LV to 75%
	Grade ≥ 3	Reduce all further doses of irinotecan, 5-FU and LV/ to 75%
**Mucositis**	Grade ≥ 2	Delay/interrupt chemotherapy until resolution to grade ≤ 1
	Grade ≥ 3 or 2nd occurrence grade ≥ 2 requiring dose delays	Reduce all further doses of 5-FU and LV to 75%
**Cardiac**	Any grade	Discontinue treatment if suspected to be related to 5-FU or bevacizumab/aflibercept
**Hand-foot-syndrome**	Grade ≥ 2	Delay/interrupt 5-FU and LV until resolution to grade ≤ 1
	Grade ≥ 3 or 2nd occurrence grade ≥ 2 requiring dose delays	Reduce all further doses of 5-FU and LV to 75%
**Other related significant organ toxicities** (except alopecia; nausea vomiting only in case of appropriate prophylaxis)	Grade ≥ 2	Delay/interrupt chemotherapy until resolution to grade ≤ 1
	Grade ≥ 3	Delay/interrupt chemotherapy until resolution to grade ≤ 1<br>Reduce all further doses of oxaliplatin/irinotecan, 5-FU and LV to 75%

Table 10. Dose modifications for chemotherapy induced toxicity

7.3.4 Toxicity and specific dose modification: oxaliplatin

Neurotoxic side effects occurring on treatment with **oxaliplatin** require the following response (see Table 11):

A small proportion of patients (1–2%) experience a syndrome of pharyngolaryngeal dysesthesia, a special form of acute neuropathy characterized by a subjective feeling of dysphagia and dyspnea with no objective evidence of airway obstruction. This unpleasant sensation is non-life-threatening and rapidly reversible without treatment. The duration of oxaliplatin infusions should be increased to 6 hours in the following cycles. The use of Calcium/Magnesium infusion before and after oxaliplatin should be introduced.

Toxicity	Toxicity lasting between 1-7 days	Toxicity lasting >7 days	Toxicity persisting between cycles
Cold-related dysaesthesia	No dose reduction	No dose reduction	Withhold oxaliplatin until recovery then restart with 25% reduction of scheduled dose<br>Omit oxaliplatin if recurs
Paraesthesia without pain	No dose reduction	No dose reduction	Withhold oxaliplatin until recovery then restart with 25% reduction of scheduled dose<br>Omit oxaliplatin if recurs
Paraesthesia with pain	No dose reduction	25% reduction of scheduled dose of oxaliplatin<br>Omit oxaliplatin if recurs	Omit oxaliplatin
Paraesthesia with functional impairment	No dose reduction	25% reduction of scheduled dose of oxaliplatin<br>Omit oxaliplatin if recurs	Omit oxaliplatin

Table 11. Dose modification for oxaliplatin-induced neuropathy

If oxaliplatin is withdrawn because of specific neurotoxicity, treatment should be continued with 5-FU/LV, bevacizumab and if applicable irinotecan until progression (study end).

Grade 3 or 4 allergic reactions require cessation of treatment with oxaliplatin. Where there is clinical evidence of pulmonary fibrosis, further treatment should be given only after excluding the disease and discontinued if fibrosis is confirmed.

7.3.5 Toxicity of irinotecan and guidelines for treatment of diarrhea

Undesirable effects of irinotecan are diarrhea 72%, nausea 55%, vomiting 41%, mucositis, e.g. stomatitis 26%, fever 33%, leukopenia 25%, and alopecia 22%.

Several clinical studies investigating irinotecan have shown that subjects who are homozygous for the UGT1A1*28 allele (also known as the “7/7” genotype) are at greater risk for irinotecan-induced severe diarrhoea or neutropenia (ACPS Advisory Committee

Meeting, 2004). Investigators should follow institutional guidelines and local medical practice for UGT1A1 genotype testing. Subjects known to be homozygous for the UGT1A1*28 allele should have the starting dose of irinotecan reduced by at least 20% (<130 mg/m^2).

Guidelines for Diarrhea Management

Symptoms of diarrhoea and/or abdominal cramping may occur at any time during a treatment cycle with irinotecan and should be managed according to the following guidelines or as per standard institutional practice.

Early diarrhoea (occurring during or shortly after infusion of irinotecan) or other cholinergic symptoms may be prevented or ameliorated by atropine if not contraindicated.

Late-onset diarrhoea (usually occurring > 24 hours after administration of irinotecan) can be life threatening or fatal if not monitored carefully by trained personnel and treated with appropriate supportive therapy. Late-onset diarrhoea associated with irinotecan should be managed aggressively according to the following guidelines adapted from Rothenberg et al. (2001).

Each subject should be given loperamide HCl (Imodium A-D) to take home upon initiation of chemotherapy. This loperamide supply should be replaced when used up. Subjects should be instructed to notify the investigator or nurse and initiate treatment with loperamide at:

1. the first episode of poorly formed or loose stools, or
2. the earliest onset of 1-2 more bowel movements per day than normally expected, or
3. an increase in stool volume or liquidity

Subjects should be instructed NOT to follow the directions on the loperamide box. Instead, they should be instructed to take 2 caplets (4 mg) initially then 1 caplet (2 mg) every 2 hours until the subject is free of diarrhoea (bowel movement) for 12 hours. During the night, the subject should take 2 caplets every 4 hours. Subjects should also be instructed to notify the investigator or nurse for the occurrence of bloody or black stools, symptoms of dehydration, fever, and inability to take liquids by mouth, inability to control diarrhoea (return to baseline) within 24 hours of using loperamide. Subjects with diarrhoea should be evaluated frequently by a nurse or physician until resolution of diarrhoea.

If diarrhoea persists for > 24 hours despite loperamide, oral fluoroquinolone antibiotic therapy should be considered.

If diarrhoea persists for > 48 hours despite loperamide, hospitalization for parenteral hydration should be considered. Appropriate antibiotics should be considered for any subject hospitalized for prolonged diarrhoea, even in the absence of fever or neutropenia and continued until resolution of diarrhoea.

Changes in electrolytes, even without BUN/urea and/or creatinine elevation, may reflect early physiologic consequences of treatment-induced gastrointestinal toxicity. Subjects with clinically significant electrolyte changes should be evaluated for dehydration and receive aggressive fluid and electrolyte replacement, if indicated.

8. Criteria of Evaluation

8.1 Progression Free Survival

8.1.1 PFS of first line treatment (PFS 1)

Time from study enrolment to date of first observed progression or death.

8.1.2 PFS after first cycle after randomization (PFS r)

Time from first cycle after randomization to date of first observed progression or death. The Progression Free Survival Rate at 6 months will be determined by the proportion of patients being alive without progressive disease 6 months after randomization.

8.1.3 PFS of second line treatment

Time from definite change of chemotherapy backbone to date of first observed progression or death (during treatment with aflibercept and changed chemotherapy).

8.2 Time to randomization

Time from study enrolment to date of first observed progression or death (equals difference between PFS 1 and PFS r)

8.3 Response Rate

Prior to 1994 the assessment of anti-tumor effect of a treatment was generally determined in accordance with the World Health Organization (WHO) criteria established in the late 1970s. The WHO criteria assess the change in tumor size using two perpendicular measurements of lesions.

The WHO criteria were reviewed in the 1990s leading to the development of a new set of criteria called Response Evaluation Criteria in Solid Tumors (RECIST) (Therasse, Arbuck et al. 2000). Recent version v1.1 of RECIST (Eisenhauer, Therasse et al. 2009) considers the change in tumor size using the sum of unidimensional measurements of the longest diameter in up to two target lesions per organ (or five in total, representing all involved organs) and also accounts for non measurable lesions.

8.3.1 RECIST Criteria

RECIST is currently accepted as the basis for assessing antitumor activity in all solid tumor types and is endorsed by regulatory authorities.

Measurement and identification of target lesions

Patients must have at least one measurable lesion, defined as > 10 mm using spiral CT or MRI. Where disease is restricted to a solitary lesion, its neoplastic nature must be confirmed by cytology/histology. Malignant lymph nodes: To be considered pathologically enlarged and measurable, a lymph node must be ≥ 15 mm in short axis when assessed by CT scan (CT scan slice thickness recommended to be no greater than 5 mm). At baseline and in follow-up, only the short axis will be measured and followed.

Baseline measurements must be taken no more than 4 weeks prior to enrollment. The same measurement technique (CT/MRI) must be used at baseline and follow up. No more than 2 target lesions per organ and 5 lesions in total, representative of all sites involved will be identified. Those with the largest diameters should be included. All other (non-target) lesions should be reported but not measured, in order that their presence or lack thereof may be tracked at follow up.

Criteria for target lesions

Complete Response (CR): Disappearance of all target lesions. Any pathological lymph nodes (whether target or non-target) must have reduction in short axis to < 10 mm.

Partial Response (PR): At least a 30 % decrease in the sum of diameters of target lesions, taking as reference the baseline sum diameters.

Progressive Disease (PD): At least a 20 % increase in the sum of diameters of target lesions, taking as reference the smallest sum on study (this includes the baseline sum if that is the smallest on study). In addition to the relative increase of 20 %, the sum must also demonstrate an absolute increase of at least 5 mm. (Note: the appearance of one or more new lesions is also considered progression).

Stable Disease (SD): Neither sufficient shrinkage to qualify for PR nor sufficient increase to qualify for PD, taking as reference the smallest sum diameters while on study.

Criteria for non-target lesions

Complete Response (CR): Disappearance of all non-target lesions and normalisation of tumor marker level. All lymph nodes must be non-pathological in size (<10mm short axis).

Non-CR/Non-PD: Persistence of one or more non-target lesion(s) and/or maintenance of tumor marker level above the normal limits.

Progressive Disease (PD): Unequivocal progression of existing non-target lesions. (Note: the appearance of one or more new lesions is also considered progression).

Response evaluation and reporting

During treatment tumor response will be assessed by the investigator according to RECIST v1.1 (CT and/or MRI) every 4 cycles (8 weeks) for the first 6 months and afterwards every 3 months. CT and/or MRI scans will be independently reviewed e.g. for resectability and allocation to the clinical groups.

At each follow up visit, response in target and non-target lesions and presence of any new lesions will be reported in the CRF. Overall response will be assigned by combining the response in target lesions, non-target lesions, and the appearance or lack of new lesions as outlined in the table below.

**Target Lesions**	**Non-target Lesions**	**New Lesions**	**Overall Response**
CR	CR	No	CR
CR	Incomplete response/SD	No	PR
PR	Non-PD	No	PR
SD	Non-PD	No	SD
PD	Any	Yes or no	PD
Any	PD	Yes or no	PD
Any	Any	Yes	PD

Table 12. Response evaluation according to RECIST

Evaluation of best overall response

The best overall response is defined as the best response from start of treatment over all follow up visits or until disease progression/recurrence whichever comes first.

8.4 Secondary resection rate

Rate of secondary R0-resections in case of resectable disease as curative approach after randomization.

8.5 Overall Survival

Overall survival will be determined as time from study enrolment to date of death.

8.6 Safety Endpoints

Safety assessments will include physical examinations including vital signs (blood pressure, heart rate, respiratory rate), ECOG, clinical laboratory profile and adverse events.

All observed toxicities and side effects will be graded according to NCI Common Terminology Criteria for Adverse Events: NCI CTCAE v4.03 (NCI 2010) for all patients and the degree of association of each with the procedure assessed and summarised.

Treatment related Serious Adverse Events rate (SAE), defined as SAEs considered possibly, probably or definitely related to treatment, will be determined.

8.7 Quality of life (QoL)

Quality of life will be assessed using the EORTC QLQ C30 questionnaire and the modules CR29 at baseline, during treatment every 8 weeks and at the end of treatment.

8.8 Further evaluations

Efficacy of the treatment according to KRAS, NRAS and BRAF status and tumor response will be analyzed retrospectively.

9. Analyses of cytokines and angiogenic factors

In plasma PlGF, Ang 2, VEGFs, G-CSF, sVEGFR2, HGF, bFGF and MMP-9 will be evaluated at screening (day -28 to -7), baseline (day1) and afterwards every 2 weeks during run-in phase and every 4 weeks during randomized phase. Furthermore, paraffin embedded tissue will be evaluated: CAIX/HIF1a, Npn-1, VEGFs, Bv8, PDGF-C, VEGFRs, CEACAM5, vascular density, KRAS, NRAS and BRAF (baseline). Optional rebiopsy at time of randomization will be performed. Upon collection tissue will be divided in three parts one for FFPE, one in RNA later and one fresh frozen at -80°C. FFPE of rebiopsy will be evaluated for CAIX/HIF1a, Npn-1, VEGFs, Bv8, PDGF-C, CXCR-4, SDF-1 and VEGFRs. RNA later and fresh frozen tissue will be stored for further analyses of angiogenic pathways as mentioned above. Further markers gaining importance during the trial will be analyzed.

9.1 Technical aspects

Venous blood draw of 7,5 ml will be performed at screening (day -28 to -7), baseline (day1) and every 2 weeks during run-in phase and every 4 weeks during randomized phase, followed by immediate plasma preparation, division into five equal aliquots, storage by -80°C (alternatively at -20°C for up to 8 weeks) and shipment to central laboratory every two months during marker driven part or after enrolment of 60% of patients and at the end of the run in phase (baseline samples will be shipped together with first 8 week assessment). Marker analysis will be performed centrally. During the

run-in phase a distinct CAF profile will be determined, which will be used for randomization trigger during the randomized part.

Patient sample will be analyzed as soon as possible and eligible patients (at least stable disease according to RECIST) will be randomized during the next treatment cycle (after $5^{th}/9^{th}/13^{th}$ or 17th cycle) between conventional switch (arm A) or early switch (arm B). Tumor imaging will be performed every 8 weeks according to RECIST v1.1. CAF assessment will be performed until end of study (progression during aflibercept containing second line).

For determination of soluble angiogenic factors (e.g. PlGF, VEGFs, bFGF, HGF) ELISAs will be used. Several CAF (hypoxia-induced factor 1α (HIF1α) and carbonic anhydrase IX (CAIX), VEGFs, PDGF, VEGFRs, Npn-1, and Bv8 will be quantified in paraffin-embedded tissue as described (Jubb, Miller et al. 2011). Vascular density will be assessed via CD31 stain. DAB stainings on TMAs will be performed as described (Vleugel, Greijer et al. 2005) and quantify DAB^+ area by means of morphometric analysis in a blinded fashion. Morphometric analysis yields a score derived from staining intensity and fraction of positive tumor cells, as previously described (Minner, Wittmer et al. 2011).

10. Assessment of Adverse Events

10.1 Definitions

An **Adverse Event (AE)** is defined as any untoward medical occurrence or experience in a subject or clinical investigation subject which occurs following the administration of the trial medication regardless of the dose or causal relationship. This can include any unfavorable and unintended signs (such as rash or enlarged liver), or symptoms (such as nausea or chest pain), an abnormal laboratory finding (including blood tests, x-rays or scans) or a disease temporally associated with the use of the protocol treatment.

Progression or deterioration of the malignancy under study (including new metastatic lesions and death due to disease progression) will be part of the efficacy assessment and should not be reported as AE or SAE. Death due to progressive disease during the study should be reported on the applicable study termination case report form with ‘death’ as reason for study termination and ‘disease progression’ as reason for death.

Worsening of a pre-existing medical condition (e.g. diabetes, migraine headaches, gout) should be considered an adverse event if there is either an increase in severity, frequency, or duration of the condition or an association with significantly worse outcomes.

Interventions for pre-treatment conditions (e.g. elective cosmetic surgery) or medical procedures that were planned before study enrolment are not considered adverse events.

The investigator is responsible for reviewing laboratory test results and determining whether an abnormal value in an individual study subject represents a change from values before the study. In general, abnormal laboratory findings without clinical significance (based on the investigator's judgment) should not be recorded as adverse events; however, laboratory value changes requiring therapy or adjustment in prior therapy are considered adverse events.

An **Adverse Drug Reaction (ADR)** is defined as any response to a medicinal product, that is noxious and unintended, related to any dose. (ICH-GCP).

Response to a medicinal product (used in the above definition) means that a causal

relationship between the medicinal product and the adverse event is at least a reasonable possibility, i.e. the relationship cannot be ruled out.

An **Unexpected Adverse Drug Reaction** is any adverse reaction for which the nature or severity is not consistent with the applicable product information (e.g. Summary of product characteristics). (ICH-GCP).

A **Serious Adverse Event (SAE)** is defined as any undesirable experience occurring to a subject, whether or not considered related to the protocol treatment. A Serious Adverse Event (SAE) which is considered related to the protocol treatment is defined as a **Serious Adverse Drug Reaction (SADR)**.

Adverse events and adverse drug reactions which are considered as **serious** are those which result in:

- death
- a life threatening event (i.e. the subject was at immediate risk of death at the time the reaction was observed)
- hospitalization or prolongation of existing hospitalization
- persistent or significant disability/incapacity
- a congenital anomaly/birth defect
- a medically significant condition, defined as an event that jeopardizes the patient or may require medical or surgical intervention to prevent one of the outcomes listed above

A hospitalization meeting the regulatory definition for “serious” is any inpatient hospital admission that includes a minimum of an overnight stay in a health care facility. Any adverse event that does not meet one of the definitions of serious (e.g. emergency room visit, outpatient surgery, or requires urgent investigation) may be considered by the investigator to meet the “other significant medical hazard” criterion for classification as a serious adverse event. Examples include allergic bronchospasm, convulsions, and blood dyscrasia.

Hospitalization for the performing of protocol-required procedures for elective procedures that have been booked in advance of enrolment that are not reasons for exclusion, or administration of study treatment is not classified as an SAE.

SUSAR: Suspected Unexpected Serious Adverse Reactions (the reference documents to assess expectedness are the summary of product characteristics).

10.2 Reporting Procedure for All Adverse Events

The investigator is responsible for ensuring that all adverse events observed by the investigator or reported by subjects are properly captured in the subjects’ medical records.

In addition, the investigator is responsible for ensuring that all adverse events captured in the subjects’ medical records (as specified above) are reported on the CRF.

Adverse events will be collected for those subjects who have provided informed consent and entered the study and will be recorded throughout the study period, beginning after the informed consent has been obtained until 28 days after the last administration of treatment in this trial.

The following adverse event attributes must be assigned by the investigator: adverse event diagnosis or syndrome(s) (if known, signs or symptoms if not known), event description (with detail appropriate to the event), dates of onset and resolution, severity, assessment of relatedness to study treatment, and action taken. The investigator may be asked to provide follow-up information, discharge summaries, and extracts from medical records or CRFs and for serious adverse events on the serious adverse event report form.

10.2.1 Assessment of Causality of Adverse Events

If applicable, the relationship of the adverse event to the study treatment will be assessed by means of the question: “Is there a reasonable possibility that the event may have been caused by the study treatment?” The investigator should respond to this question with either Yes or No.

If the adverse event occurred after informed consent but before initiation of study treatment, the relationship of the adverse event to study screening is to be assessed by means of the question: “Is there a reasonable possibility that the event may have been caused by the study screening procedures/treatment?” The investigator should respond to this question with either Yes or No. If the answer is Yes, record what part of the study screening procedures/treatment is suspected.

An adverse event will not be considered possibly related to study treatment if it

- may be judged to be due to extraneous causes such as disease or environment or toxic factors.
- may be judged to be due to the subject’s clinical state or other therapy being administered.
- is not biologically plausible that the event is related to study medication.
- does not reappear or worsen when study treatment is re-administered.
- does not follow a temporal sequence from administration of study treatment.

An adverse event will be considered possibly related to study treatment if it:

- follows a temporal sequence from administration of study treatment.
- is a known response to the investigational product based on clinical or preclinical data.
- could not be explained by the known characteristics of the subject’s clinical state, environmental or toxic factors, or other therapy administered to the subject.
- disappears or decreases upon cessation or reduction of dose of study treatment.
- reappears or worsens when study treatment is re-administered.

Medically significant i.e. serious adverse events will be followed until resolved or considered stable. It will be left to the investigator’s clinical judgment to determine whether an adverse event is related and of sufficient severity to require the subject’s removal from treatment or from the study. A subject may also voluntarily withdraw from treatment due to what he or she perceives as an intolerable adverse event. If either of these situations arises, the subject should be strongly encouraged to undergo a safety follow-up assessment and be under medical supervision until symptoms cease or the condition becomes stable.

10.2.2 Assessment of Severity of Adverse Events

The severity of adverse events will be graded according to the CTCAE version 4.03.

When an adverse event cannot be graded by CTCAE version 4.03, the following severity grades are to be used:

1 = mild
2 = moderate
3 = severe
4 = life-threatening or disabling
5 = fatal

10.3 Serious Adverse Events Reporting

Serious adverse events will be collected and recorded throughout the study period, beginning after informed consent has been obtained until 28 days after the last administration of treatment in this trial.

Additionally all serious adverse events related to study medication (= serious adverse drug reactions) must be recorded through the follow-up visits which occur 18 months after last study drug administration.

The investigator must **unhesitatingly** (within 24 hours) report all serious adverse events on a separate SAE report form to Assign Safety Desk.

Please fax the report to:

Assign Safety Desk

Stadlweg 23, 6020 Innsbruck, Austria

FAX: +43 512 281514

Tel: +43 676 844033835

Mail: SafetyDesk@assigngroup.com

Follow-up information and supporting documents should also be reported within 1 business day and can be sent by fax or email to Assign Safety Desk.

The sponsor will medically review all SAEs. The sponsor is responsible for ensuring that all reporting requirements to all concerned investigators, to the Central Ethics Committees, and to Regulatory Authorities are fulfilled. In accordance with the legal requirements (Directives 2005/28/EC and 2001/20/EC, GCP-V and the German Drug Law and other local regulations) all Adverse Drug Reactions that are both serious and unexpected are subject to expedited reporting. Annual Safety Reports will be sent to the Central Ethics Committees and the competent authorities.

11. Data Analysis and Statistical Considerations

11.1 Run in Phase for determination of CAF profile based on PIGF and VEGF-B for early detection of progression

11.1.1 Prognostic value of baseline values

Univariate and multivariate semi parametric Cox models for PFS/response with the CAF level(s) as either continuous or dichotomized variables will be fit. Fractional polynomials will be fit to explore the continuous relation biomarker level and hazard of PFS. Multivariable Cox proportional hazards regression for PFS/response with CAF levels will be used based on a bootstrap approach over cases (Sauerbrei and Schumacher 1992), that has a nested selection procedure over all parameter subsets (evaluation of all

parameter subsets and Akaike information criterion) to determine the most relevant parameters in each resampling step, i.e. only the most frequent variables from all runs are used in accordance with adjustment of the number of variables to the number of events during follow-up. This together with embedded likelihood ratio tests will enable to select a parsimonious model with acceptable goodness of fit.

For combining individual markers into single combination scores a methodology will be used that maximizes the rank correlation of the linear combination of the markers and a target (non-negative correlation maximization, see (Song, von Figura et al. 2010)). Determining the appropriate combination under the rank and non-negative constraints is performed via population based optimization strategies. Non-negativity constraints ensure a parts-based representation as only additive, not subtractive, combinations are allowed.

11.1.2 Determination of the association of repeated measurement of CAF levels particularly PIGF and VEGF-B on PFS/response status

Various statistical methods will be used depending on the timeliness of the measurement:

1. Cox model for PFS with the longitudinal measurements of CAF (or the percent changes from baseline) as time dependent covariates.
2. Landmark analyses using the status of CAF at time t as covariates for PFS/response after time t.
3. A linear mixed effect model may also be investigated with random effect on the intercept and the time variable to account for both missing data and intercept correlation.

Receiver operating characteristic curves (ROC) will be used to evaluate the prognostic performance of the CAF level on PFS/response over time (Heagerty and Zheng 2005). The Harrel statistic adapted to survival data will be used.

The potential association between CAF level and clinico-pathologic data (demographic, clinical, and immunohistochemical variables) in patients will be explored by running linear regression models.

11.2 Independent data monitoring committee (IDMC)

An independent data monitoring committee (IDMC) will follow the progress of the clinical trial. After completing the run in phase of the study, with at least 30 patients completing their first line treatment (progression, secondary resection, toxicity) and being evaluable for CAF analyses, the results will be reviewed by the IDMC. Based on that review the decision to continue with, modify or cancel the randomized part will be made. After the 50th patient completing first line treatment IDMC will meet again to review the available data and decide about modification, cessation or continuation of the randomized part.

11.3 Randomized Phase - Sample Size Calculation

The majority of patients continuing treatment with bevacizumab and mFOLFOX6 after change of CAF-profile compared baseline are expected to progress at the next scheduled staging (within two months). Thus, PFSR@6 months after randomization will be 20% for arm A (conventional switch). The early marker-driven switch is expected to result in a PFSR@6 of at least 45%. The trial should achieve 80% power to detect differences between the treatment arms and keeping the type I error level below 10%.

The planned number of patients with a drop out rate of about 10% will be 43 patients per

arm. Thus, 86 patients need to be randomized. About 60% of patients will be eligible for randomization (at least stable disease and distinct maker profile) so all together 150 patients are required for the randomized part.

The trial uses a randomized parallel arm phase II design.

11.4 Populations for Analysis

The All Enrolled population will include all patients with a signed ICF.

The Intention-to-treat (ITT) population will include all randomized patients in the study. All patients will be grouped according to their randomization regardless of actual treatment received.

The Per-protocol (PP) population will include all patients without major protocol deviations who receive at least one treatment cycle and who were treated according to their randomization schedule.

The Safety population consists of all patients who received at least one treatment cycle. All patients will be grouped according to their actual treatment received

11.5 Patient Demographics/Other Baseline Characteristics

The following demographic and baseline characteristics will be summarized descriptively by analysis population / treatment group:

- Gender and age
- ECOG performance status
- Tumor marker (CEA and optional CA 19-9)
- Disease status
- Other characteristics (e.g liver chemistry)
- KRAS, NRAS and BRAF mutational status

Medical history will be summarized by primary body system organ class and preferred term (according to NCI CTCAE v4.03).

About 60% males and 40% females are expected in the study population. No gender-specific differences with respect to primary and secondary endpoints are expected, but gender as a baseline characteristic will be summarized descriptively by analysis population / treatment group.

11.6 Treatments (study treatments)

The number and dose of treatment cycles will be summarized by treatment group.

11.7 Efficacy Analysis

11.7.1 Primary Efficacy Endpoint

The primary endpoint of the run-in phase with conventional switch of chemotherapy together with the anti-angiogenic agent is:

- Progression free survival (PFS1) of first line treatment

The primary endpoint of the randomized part with marker-driven switch of antiangiogenic agent and maintenance of chemotherapy is:

- Progression free survival rate at 6 months (PFSR@6) after first cycle after randomization.

11.7.2 Secondary Efficacy Endpoints

The secondary efficacy endpoints will be the following variables:

- Predictive value of cytokines and angiogenic factor (CAF) particularly PlGF and VEGF-B for early detection of progression during treatment with chemotherapy and bevacizumab
- Determination and validation of a CAF profile based on PlGF and VEGF-B predicting tumor progression before radiologic progression
- PFS1, after first cycle after randomization (PFSr) and of second line treatment (PFS2)
- Time to randomization (TTR)
- Overall survival (OS)
- Overall response rate (RR) according to RECIST v1.1
- Secondary resection rate (sRR)
- Toxicity (Safety assessments will include physical examinations (blood pressure, heart rate, respiratory rate), vital signs, ECOG, clinical laboratory profile and monitoring of adverse events, according to NCI CTCAE v4.03)
- Quality of life using the EORTC QLQ-C30 and the modules CR29
- Changes in CAF during early marker-driven switch and conventional treatment approach
- Prognostic value of CAF at baseline and/or during treatment

Response rates and secondary resection rate will be summarized using frequency tables

For the time-to-event variables PFS and OS, the Kaplan-Meier method will be used to estimate the event free survival, and the log-rank test will be conducted to compare the two treatment groups.

Toxicity and Quality of life will be documented in a descriptive way.

All secondary efficacy analyses will be based on the all enrolled and ITT population and the corresponding statistical testing results will be interpreted in an exploratory sense.

12. Data management

12.1 Randomization Procedure

For randomization the following data are needed:

CAF levels and current tumor response according to RECIST.

Randomization to study treatment should occur within seven days after eligibility criteria have been met. Upon confirmation of eligibility, study subjects will be randomized to one of two treatment arms in 1:1 ratio via the eCRF system.

- Arm A (conventional switch of chemotherapy together with the anti-angiogenic agent)
- Arm B (early marker-driven switch of anti-angiogenic agent and maintenance of chemotherapy)

Patient identification list

All enroled patients have to be documented in a confidential patient identification list (subject enrolment log). This list contains the patient specific numbers (patient- and randomization-number) together with date of birth and the full name of the patient. Patient

related data will be just transmitted in pseudonymized form. The identification list will stay at the center.

12.2 Data capture

All data will be entered directly at the center by the site staff with the eCRF system InterTrial. Data will be evaluated for consistency, accuracy and completeness. After completion of data capture the clinical database will be closed and the data will be transferred for statistical analysis.

13. Quality assurance

13.1 Standardization

Criteria for assessing efficacy and safety endpoints will be standardized by using NCI-CTCAE version 4.03 for safety issues and RECIST version 1.1 for efficacy parameters. Every center has to reveal their laboratory norm values and their validation through certification.

13.2 Data access

All source data have to be in the patients file under the responsibility of the investigator. Documentation in the eCRF must correspond to source data in the patient file. For this trial source data are defined as:

- medical and demographical data
- results of laboratory and imaging data
- selection criteria
- signed informed consent form (original)

13.3 Monitoring/ Source Data Verification (SDV)

The monitoring will be conducted by Assign International GmbH. There will be central monitoring by reviewing the data being entered into the eCRF (InterTrial) as well as on-site monitoring. The frequency of on-site visits will depend on the number of recruited patients. The access to trial specific data for the monitor will be ensured through the cooperation treaty between the sponsor and the study site. The monitor must be given access to subject medical records and other study-related records needed to verify the entries on the eCRF. The investigator agrees to cooperate with the monitor to ensure that any problems detected in the course of these monitoring visits, including delays in completing case report forms, are resolved. The investigator has to ensure that all data required according to this protocol will be entered promptly in the eCRF. Data collected on each subject will be recorded in the subjects' medical records and the eCRF.

Quality control of data will be done by reviewing the data entered into the trial software for consistency, accuracy and completeness. During on-site visits the correct transmission of data into the eCRF (source data verification) as well as informed consent forms, selection criteria, efficacy and safety parameters will be reviewed. The complete scale of the monitoring will be defined by the trial specific monitoring plan.

13.4 Audits and Inspections

To ensure quality of data, study integrity, and compliance with the protocol and the various applicable regulations and guidelines, the sponsor may conduct site visits to institutions participating to protocols.

The investigator, by accepting to participate to this protocol, agrees to co-operate fully with any quality assurance visit undertaken by third parties, including representatives from the sponsor, national and/or foreign regulatory authorities or company supplying the product under investigation, as well as to allow direct access to documentation pertaining to the clinical trial (including CRFs, source documents, hospital subject charts and other study files) to these authorized individuals.

The investigator must inform the sponsor immediately in case a regulatory authority inspection will be scheduled.

14. Regulatory and Legal Obligations

14.1 General provisions/Declaration of Helsinki

This study is conducted in agreement with the German Drug Law (AMG 1976 in Novelles), ICH Harmonized Tripartite Guideline on Good Clinical Practice, valid since 17.01.1997 (Appendix 11) the „Verordnung über die Anwendung der Guten Klinischen Praxis bei der Durchführung von klinischen Prüfungen mit Arzneimitteln zur Anwendung am Menschen“ from August 9th 2004 (recent update from 19th October 2012) and the Declaration of Helsinki (recent version). The Principle Investigator has more than two years of experience in the conduction of clinical drug trials.

14.2 Patient Protection

The responsible investigator will ensure that this study is conducted in agreement with either the Declaration of Helsinki (recent version) or the laws and regulations.

The protocol has been written, and the study will be conducted according to the ICH Harmonized Tripartite Guideline for Good Clinical Practice (reference: http://www.ifpma.org/pdfifpma/e6.pdf).

The protocol will be approved by Independent Ethics Committees.

14.3 Competent authority

Prior to the start of the trial an application for authorisation by the competent Higher Federal Authority is submitted by the sponsor including a copy of the protocol and other information and documents required by the competent Higher Federal Authority. If requested, modifications must be incorporated.

Contact address for participating subjects at the competent authority in Germany:

Paul-Ehrlich-Institut
Bundesinstitut für Impfstoffe und biomedizinische Arzneimittel
Paul-Ehrlich-Str. 51-59
63225 Langen
Telefon: +49 6103 77 1810
Fax: +49 6103 77 1277
E-Mail: klinpruefung@pei.de

14.4 Independent Ethics Committee

Prior to the start of the trial an application for the favourable opinion is submitted by the sponsor to the central independent, interdisciplinary ethics committee and to responsible local independent ethics committees as applicable. A copy of the written approval of the protocol and informed consent form must be available before the start of recruitment of

subjects into the study. All changes of the study protocol as well as all presumable unexpected heavy events linked to the study medication, will be announced to the IEC. Once a year or whenever it is questioned the IEC will get information about all SAR and about the security of the affected subjects. Recommendations and tips of the IEC will be taken up into the study protocol. The sponsor will inform the IEC about the course of the Investigation in security aspects and also about the end and the results of the investigation.

The investigator can not influence the decisions of the IEC. A list of the IEC members and the IEC rules will be ordered.

14.5 Amendments

The appendices, attached to this protocol and referred to in the protocol, form an integral part of the protocol. No changes or amendments to this protocol may be made by the Investigator. The sponsor must submit and obtain approval from the IEC and competent Higher Federal Authority for all subsequent substantial protocol amendments. For changes to the informed consent form approval from the IEC has to be obtained.

14.6 Study Reports

A clinical trial report will be written and provided to the IEC and competent Higher Federal Authority independent of the completion or a premature closure of the trial.

14.7 Informed Consent

The informed consent form will be submitted together with the study protocol to the independent ethics committees (IEC) for review and approval. If requested, modifications must be incorporated. A copy of the written approval of the IEC must be available before starting the trial and dispensing any trial medication to trial subjects. The informed consent form must not be altered by the investigator except for contact data of the investigators. Changes to the informed consent form also have to be approved by the IEC. The revised form will be sent to all sites to replace the preceding version.

Before a subject's participation in the clinical study, the investigator must obtain written informed consent from the subject. All subjects will be informed of the aims of the study, the possible adverse events, the anticipated benefits, the procedures and possible hazards to which he/she will be exposed, and the mechanism of treatment allocation the subjects also will be informed about alternative treatments. Subjects will be informed of their insurance protection and the obligations which are linked to insurance. They will be informed as to the strict confidentiality of their subject data, but that their medical records may be reviewed for trial purposes by authorized individuals other than their treating physician. It will be emphasized that the participation is voluntary and that the subject is allowed to refuse further participation in the protocol whenever he/she wants. This will not prejudice the subject's subsequent care. The informed consent procedure must conform to the ICH guidelines on Good Clinical Practice.

The informed consent consists of three parts: consent to the diagnostic and therapeutic procedures of the trial, consent to the collection and storage of biological material, and consent to the processing and storage of data. The latter one includes consent to inspections where records may be reviewed by authorized individuals (other than their treating physician) of the sponsor or surveillance authorities / ethics committees. If the subject does not consent to the collection, processing and storage of his data, inclusion in the study is not possible and the subject's refusal should be documented in the medical notes. The subject must be informed about the aims, methods, anticipated benefits, and

potential hazards of the study and before any protocol-specific screening procedures or any study treatment are administered. The collection and storage of biological material taken by a biopsy in this clinical trial is optional; consent to this part of the trial is not necessary for the participation in this clinical trial.

The investigator is also responsible for asking the subject if the subject agrees to have his/her primary care physician informed of the subject’s participation in the clinical study. If the subject agrees to such notification, the investigator shall inform the subject’s primary care physician of the subject’s participation in the clinical study.

If a potential subject is illiterate or visually impaired, the investigator must provide an impartial witness to read the informed consent form to the subject and must allow for questions. Thereafter, both the subject and the witness must sign the informed consent form to attest that informed consent was freely given and understood.

Adequate explanations of the aims, methods, anticipated benefits, and potential hazards of the study, the mechanism of treatment allocation must be given. The subject will have enough time to decide to participate in the study or not.

The acquisition of informed consent and the subject’s agreement or refusal of his/her notification of the primary care physician must be documented in the subject’s medical records, and the informed consent form must be signed and personally dated by the subject and by the investigator. One signed original of the informed consent form must be retained in accordance with institutional policy and another original must be provided to the subject. Treatment cannot start before the subject has signed the informed consent, meets all inclusion and no exclusion criteria and is registered.

With signing the informed consent form the investigator confirms that an individual clarification conversation has taken place and that the subject has signed the informed consent form.

14.8 Subject Confidentiality

The investigator must ensure that the subject’s confidentiality is maintained. On the case report forms, subjects should be identified by their subject study number and only on the SAE report form additionally with date of birth.

In compliance with ICH GCP Guidelines, it is required that the investigator and institution permit authorized representatives of the sponsor, of regulatory agencies, and the IEC direct access to review the subject’s original medical records for verification of study-related procedures and data. Direct access includes examining, analyzing, verifying, and reproducing any records and reports that are important to the evaluation of the study. The investigator is obligated to inform and obtain the consent of the subject to permit named representatives to have access to his/her study-related records without violating the confidentiality of the subject. The investigator must keep a list for the identification of the subjects (including name, birthday, gender, date of informed consent, date of randomization / registration).

14.9 Study Documentation and Archive

The investigator must maintain a list of appropriately qualified persons to whom he/she has delegated study duties, including all those authorized to make entries and/or corrections on case report forms.

Source documents are original documents, data, and records from which the subject’s case report form data are obtained. These include but are not limited to hospital records,

clinical and office charts, laboratory and pharmacy records, diaries, microfiches, radiographs, and correspondence.

The investigator and study staff are responsible for maintaining a comprehensive and centralized filing system of all study-related (essential) documentation, suitable for inspection at any time by representatives from the study sponsor and/or applicable regulatory authorities. Elements include:

- Subject files containing completed case report forms, informed consent forms, and subject identification list.
- Study files containing the protocol with all amendments, the summary of product characteristics, copies of pre-study documentation, and all correspondence to and from the IEC.
- If kept, proof of receipt, Investigational Product Accountability Record, Return of Investigational Product for Destruction, Final Investigational Product Reconciliation Statement, and all drug-related correspondence.

In addition, all original source documents supporting entries in the case report forms must be maintained and be readily available.

All study documents and source documents must be kept for at least 10 years from submission of the final study report. Should the investigator wish to assign the study records to another party or move them to another location, he/she must notify the sponsor in writing of the new responsible person and/or the new location.

14.10 Compensation

Subjects will not be paid for participating in this clinical trial.

15. Trial Sponsorship and Financing

University of Ulm is the legal sponsor of the trial and finances the trial. Financial support for the conduction of the trial is granted by Sanofi.

16. Trial Insurance

For all subjects participating in the trial the sponsor has taken out a liability insurance policy (mentioned below) according § 40 Abs. 1 Nr. 8 und Abs. 3 German drug law (AMG) (for Germany) or other applicable local laws and regulations, which covers the sponsor, the investigator and his co-workers against liability in the event that a subject's health is injured during the course of the clinical trial. The insurance policies provide benefits, even when no one else is liable for the damage death of or injury to any subject during the trial.

A certificate of insurance will be provided to the investigators and the subjects.

Contact Adress of the insurance company:

HDI-Gerling Industrie Versicherung AG
Riethorst 2
30659 Hannover

17. Publication Policy

After receiving the biometrical results a final report will be published and further publications (abstracts etc.) will be done. All participating sites recruiting at least 10% of the patients will become a co-authorship if possible according to the publication policy of the journal. Persons involved in planning, conducting and evaluating the trial will be offered co-authorships. All co-authors will get the option to comment on the manuscript before publication.

Appendix A: Bibliography

Amado, R. G., M. Wolf, et al. (2008). "Wild-type KRAS is required for panitumumab efficacy in patients with metastatic colorectal cancer." J Clin Oncol **26**(10): 1626-1634.

Arkenau, H. T., D. Arnold, et al. (2008). "Efficacy of oxaliplatin plus capecitabine or infusional fluorouracil/leucovorin in patients with metastatic colorectal cancer: a pooled analysis of randomized trials." J Clin Oncol **26**(36): 5910-5917.

Arnold, D., Andre, T. (2012). "Bevacizumab (BEV) plus chemotherapy (CT) continued beyond first progression in patients with metastatic colorectal cancer (mCRC) previously treated with BEV plus CT: Results of a randomized phase III intergroup study (TML study)." J Clin Oncol **30**(suppl): abstr CRA3503.

Batchelor, T. T., A. G. Sorensen, et al. (2007). "AZD2171, a pan-VEGF receptor tyrosine kinase inhibitor, normalizes tumor vasculature and alleviates edema in glioblastoma patients." Cancer Cell **11**(1): 83-95.

Bergers, G. and D. Hanahan (2008). "Modes of resistance to anti-angiogenic therapy." Nat Rev Cancer **8**(8): 592-603.

Bokemeyer, C., I. Bondarenko, et al. (2011). "Efficacy according to biomarker status of cetuximab plus FOLFOX-4 as first-line treatment for metastatic colorectal cancer: the OPUS study." Ann Oncol **22**(7): 1535-1546.

Bokemeyer, C., E. V. Cutsem, et al. (2012). "Addition of cetuximab to chemotherapy as first-line treatment for KRAS wild-type metastatic colorectal cancer: Pooled analysis of the CRYSTAL and OPUS randomised clinical trials." Eur J Cancer **48**(10): 1466-1475.

Brown, J. M. (2007). "Tumor hypoxia in cancer therapy." Methods Enzymol **435**: 297-321.

Casanovas, O., D. J. Hicklin, et al. (2005). "Drug resistance by evasion of antiangiogenic targeting of VEGF signaling in late-stage pancreatic islet tumors." Cancer Cell **8**(4): 299-309.

Cassidy, J., Cunningham, D. (2008). "Surgery with curative intent in patients (pts) treated with first-line chemotherapy (CT) + bevacizumab (BEV) for metastatic colorectal cancer (mCRC): First BEAT and NO16966." J Clin Oncol **26**(May 20 suppl): abstr 4022.

Chen, C. N., F. J. Hsieh, et al. (2004). "The significance of placenta growth factor in angiogenesis and clinical outcome of human gastric cancer." Cancer Lett **213**(1): 73-82.

Cunningham, D., Y. Humblet, et al. (2004). "Cetuximab monotherapy and cetuximab plus irinotecan in irinotecan-refractory metastatic colorectal cancer." N Engl J Med **351**(4): 337-345.

Douillard, J. Y., D. Cunningham, et al. (2000). "Irinotecan combined with fluorouracil compared with fluorouracil alone as first-line treatment for metastatic colorectal cancer: a multicentre randomised trial." Lancet **355**(9209): 1041-1047.

Douillard, J. Y., Siena, S. (2011). "Final results from PRIME: Randomized phase III study of panitumumab (pmab) with FOLFOX4 for first‑line metastatic colorectal cancer (mCRC)." J Clin Oncol **29**(suppl): abstr 3510.

Ebihara, Y., M. J. Xu, et al. (2000). "Exclusive expression of G-CSF receptor on myeloid progenitors in bone marrow CD34+ cells." Br J Haematol **109**(1): 153-161.

Ebos, J., C. Lee, et al. (2009). "Acceleration of metastasis after short-term treatment with a potent inhibitor of angiogenesis." Cancer Cell **15**(in press).

Eisenhauer, E. A., P. Therasse, et al. (2009). "New response evaluation criteria in solid tumours: revised RECIST guideline (version 1.1)." Eur J Cancer **45**(2): 228-247.

Escudero-Esparza, A., T. A. Martin, et al. (2009). "PGF isoforms, PLGF-1 and PGF-2, in colorectal cancer and the prognostic significance." Cancer Genomics Proteomics **6**(4): 239-246.

Escudero-Esparza, A., T. A. Martin, et al. (2010). "PGF isoforms, PLGF-1 and PGF-2 and the PGF receptor, neuropilin, in human breast cancer: prognostic significance." Oncol Rep **23**(2): 537-544.

Fan, F., S. Samuel, et al. (2011). "Chronic exposure of colorectal cancer cells to bevacizumab promotes compensatory pathways that mediate tumour cell migration." Br J Cancer **104**(8): 1270-1277.
Ferlay, J., H. R. Shin, et al. (2010). "Estimates of worldwide burden of cancer in 2008: GLOBOCAN 2008." Int J Cancer.
Fischer, C., B. Jonckx, et al. (2007). "Anti-PlGF inhibits growth of VEGF(R)-inhibitor-resistant tumors without affecting healthy vessels." Cell **131**(3): 463-475.
Fischer, C., M. Mazzone, et al. (2008). "FLT1 and its ligands VEGFB and PlGF: drug targets for anti-angiogenic therapy?" Nat Rev Cancer **8**(12): 942-956.
Fuchs, C. S., J. Marshall, et al. (2007). "Randomized, controlled trial of irinotecan plus infusional, bolus, or oral fluoropyrimidines in first-line treatment of metastatic colorectal cancer: results from the BICC-C Study." J Clin Oncol **25**(30): 4779-4786.
Giantonio, B. J., P. J. Catalano, et al. (2007). "Bevacizumab in combination with oxaliplatin, fluorouracil, and leucovorin (FOLFOX4) for previously treated metastatic colorectal cancer: results from the Eastern Cooperative Oncology Group Study E3200." J Clin Oncol **25**(12): 1539-1544.
Goede, V., O. Coutelle, et al. "Identification of serum angiopoietin-2 as a biomarker for clinical outcome of colorectal cancer patients treated with bevacizumab-containing therapy." Br J Cancer **103**(9): 1407-1414.
Grothey, A., Sobrero, A. (2012). "Results of a phase III randomized, double-blind, placebo-controlled, multicenter trial (CORRECT) of regorafenib plus best supportive care (BSC) versus placebo plus BSC in patients (pts) with metastatic colorectal cancer (mCRC) who have progressed after standard therapies." J Clin Oncol **30**(suppl 4): abstr LBA 385.
Grothey, A., M. M. Sugrue, et al. (2008). "Bevacizumab beyond first progression is associated with prolonged overall survival in metastatic colorectal cancer: results from a large observational cohort study (BRiTE)." J Clin Oncol **26**(33): 5326-5334.
Harris, A. L. (2002). "Hypoxia--a key regulatory factor in tumour growth." Nat Rev Cancer **2**(1): 38-47.
Heagerty, P. J. and Y. Zheng (2005). "Survival model predictive accuracy and ROC curves." Biometrics **61**(1): 92-105.
Ho, M. C., C. N. Chen, et al. (2007). "Placenta growth factor not vascular endothelial growth factor A or C can predict the early recurrence after radical resection of hepatocellular carcinoma." Cancer Lett **250**(2): 237-249.
Hurwitz, H., L. Fehrenbacher, et al. (2004). "Bevacizumab plus irinotecan, fluorouracil, and leucovorin for metastatic colorectal cancer." N Engl J Med **350**(23): 2335-2342.
Jain, R. K., D. G. Duda, et al. (2009). "Biomarkers of response and resistance to antiangiogenic therapy." Nat Rev Clin Oncol **6**(6): 327-338.
Jemal, A., F. Bray, et al. (2011). "Global cancer statistics." CA Cancer J Clin **61**(2): 69-90.
Jonker, D. J., C. J. O'Callaghan, et al. (2007). "Cetuximab for the treatment of colorectal cancer." N Engl J Med **357**(20): 2040-2048.
Jubb, A. M. and A. L. Harris (2010). "Biomarkers to predict the clinical efficacy of bevacizumab in cancer." Lancet Oncol **11**(12): 1172-1183.
Jubb, A. M., K. D. Miller, et al. (2011). "Impact of exploratory biomarkers on the treatment effect of bevacizumab in metastatic breast cancer." Clin Cancer Res **17**(2): 372-381.
Karapetis, C. S., S. Khambata-Ford, et al. (2008). "K-ras mutations and benefit from cetuximab in advanced colorectal cancer." N Engl J Med **359**(17): 1757-1765.
Kohne, C. H., J. De Greve, et al. (2008). "Irinotecan combined with infusional 5-fluorouracil/folinic acid or capecitabine plus celecoxib or placebo in the first-line treatment of patients with metastatic colorectal cancer. EORTC study 40015." Ann Oncol **19**(5): 920-926.
Koopman, M., N. F. Antonini, et al. (2007). "Sequential versus combination chemotherapy with capecitabine, irinotecan, and oxaliplatin in advanced colorectal cancer (CAIRO): a phase III randomised controlled trial." Lancet **370**(9582): 135-142.
Kopetz, S., P. M. Hoff, et al. (2010). "Phase II trial of infusional fluorouracil, irinotecan, and bevacizumab for metastatic colorectal cancer: efficacy and circulating angiogenic biomarkers associated with therapeutic resistance." J Clin Oncol **28**(3): 453-459.

Lieu, C., Tran, H.T. (2011). "The association of alternate VEGF ligands with resistance to anti-VEGF therapy in metastatic colorectal cancer." J Clin Oncol **29**(suppl): abstr 3533.
Loupakis, F., C. Cremolini, et al. (2011). "Pharmacodynamic and pharmacogenetic angiogenesis-related markers of first-line FOLFOXIRI plus bevacizumab schedule in metastatic colorectal cancer." Br J Cancer **104**(8): 1262-1269.
Matsumoto, K., K. Suzuki, et al. (2003). "Prognostic significance of plasma placental growth factor levels in renal cell cancer: an association with clinical characteristics and vascular endothelial growth factor levels." Anticancer Res **23**(6D): 4953-4958.
Maughan, T. S., R. A. Adams, et al. (2011). "Addition of cetuximab to oxaliplatin-based first-line combination chemotherapy for treatment of advanced colorectal cancer: results of the randomised phase 3 MRC COIN trial." Lancet **377**(9783): 2103-2114.
Minner, S., C. Wittmer, et al. (2011). "High level PSMA expression is associated with early PSA recurrence in surgically treated prostate cancer." Prostate **71**(3): 281-288.
Murdoch, C., M. Muthana, et al. (2008). "The role of myeloid cells in the promotion of tumour angiogenesis." Nat Rev Cancer **8**(8): 618-631.
Muro, K., N. Boku, et al. (2010). "Irinotecan plus S-1 (IRIS) versus fluorouracil and folinic acid plus irinotecan (FOLFIRI) as second-line chemotherapy for metastatic colorectal cancer: a randomised phase 2/3 non-inferiority study (FIRIS study)." Lancet Oncol **11**(9): 853-860.
Nikolinakos, P. G., N. Altorki, et al. (2010). "Plasma cytokine and angiogenic factor profiling identifies markers associated with tumor shrinkage in early-stage non-small cell lung cancer patients treated with pazopanib." Cancer Res **70**(6): 2171-2179.
Okines, A., O. D. Puerto, et al. (2009). "Surgery with curative-intent in patients treated with first-line chemotherapy plus bevacizumab for metastatic colorectal cancer First BEAT and the randomised phase-III NO16966 trial." Br J Cancer **101**(7): 1033-1038.
Paez-Ribes, M., E. Allen, et al. (2009). "Anti-angiogenic therapy elicits malignant progression of tumors to increased local invasion and distant metastasis." Cancer Cell **in press**(March, 5).
Parr, C., G. Watkins, et al. (2005). "Placenta growth factor is over-expressed and has prognostic value in human breast cancer." Eur J Cancer **41**(18): 2819-2827.
Peeters, M., T. J. Price, et al. (2010). "Randomized phase III study of panitumumab with fluorouracil, leucovorin, and irinotecan (FOLFIRI) compared with FOLFIRI alone as second-line treatment in patients with metastatic colorectal cancer." J Clin Oncol **28**(31): 4706-4713.
Price, T. J., J. E. Hardingham, et al. (2011). "Impact of KRAS and BRAF Gene Mutation Status on Outcomes From the Phase III AGITG MAX Trial of Capecitabine Alone or in Combination With Bevacizumab and Mitomycin in Advanced Colorectal Cancer." J Clin Oncol **29**(19): 2675-2682.
Rini, B. I., M. D. Michaelson, et al. (2008). "Antitumor activity and biomarker analysis of sunitinib in patients with bevacizumab-refractory metastatic renal cell carcinoma." J Clin Oncol **26**(22): 3743-3748.
Saltz, L. B., S. Clarke, et al. (2008). "Bevacizumab in combination with oxaliplatin-based chemotherapy as first-line therapy in metastatic colorectal cancer: a randomized phase III study." J Clin Oncol **26**(12): 2013-2019.
Sathornsumetee, S., Y. Cao, et al. (2008). "Tumor angiogenic and hypoxic profiles predict radiographic response and survival in malignant astrocytoma patients treated with bevacizumab and irinotecan." J Clin Oncol **26**(2): 271-278.
Sauerbrei, W. and M. Schumacher (1992). "A bootstrap resampling procedure for model building: application to the Cox regression model." Stat Med **11**(16): 2093-2109.
Shojaei, F., X. Wu, et al. (2007). "Tumor refractoriness to anti-VEGF treatment is mediated by CD11b+Gr1+ myeloid cells." Nat Biotechnol **25**(8): 911-920.
Shojaei, F., X. Wu, et al. (2007). "Bv8 regulates myeloid-cell-dependent tumour angiogenesis." Nature **450**(7171): 825-831.
Sobrero, A. F., J. Maurel, et al. (2008). "EPIC: phase III trial of cetuximab plus irinotecan after fluoropyrimidine and oxaliplatin failure in patients with metastatic colorectal cancer." J Clin Oncol **26**(14): 2311-2319.

Song, Z., G. von Figura, et al. (2010). "Lifestyle impacts on the aging-associated expression of biomarkers of DNA damage and telomere dysfunction in human blood." Aging Cell **9**(4): 607-615.
Tabernero, J., Van Cutsem, E. (2011). "Results From VELOUR, a Phase III Study of Aflibercept (A) Versus Placebo (pbo) in Combination with FOLFIRI for the Treatment of Patients (pt) with Previously Treated Metastatic Colorectal Cancer (MCRC)." Eur J Cancer **47**(Supplement 2, September 2011): LBA 19.
Therasse, P., S. G. Arbuck, et al. (2000). "New guidelines to evaluate the response to treatment in solid tumors. European Organization for Research and Treatment of Cancer, National Cancer Institute of the United States, National Cancer Institute of Canada." J Natl Cancer Inst **92**(3): 205-216.
Tournigand, C., T. Andre, et al. (2004). "FOLFIRI followed by FOLFOX6 or the reverse sequence in advanced colorectal cancer: a randomized GERCOR study." J Clin Oncol **22**(2): 229-237.
Van Cutsem, E., C. H. Kohne, et al. (2011). "Cetuximab Plus Irinotecan, Fluorouracil, and Leucovorin As First-Line Treatment for Metastatic Colorectal Cancer: Updated Analysis of Overall Survival According to Tumor KRAS and BRAF Mutation Status." J Clin Oncol **29**(15): 2011-2019.
Van Cutsem, E., M. Peeters, et al. (2007). "Open-label phase III trial of panitumumab plus best supportive care compared with best supportive care alone in patients with chemotherapy-refractory metastatic colorectal cancer." J Clin Oncol **25**(13): 1658-1664.
Van Cutsem, E., F. Rivera, et al. (2009). "Safety and efficacy of first-line bevacizumab with FOLFOX, XELOX, FOLFIRI and fluoropyrimidines in metastatic colorectal cancer: the BEAT study." Ann Oncol **20**(11): 1842-1847.
Van Cutsem, E., Tabernero, J. (2011). "Intravenous (iv) aflibercept versus placebo in combination with irinotecan/5-FU (FOLFIRI) for second line treatmnet of metastatic colorectal cancer (MCRC): Results of a multinational phase III trial (EFC10262-VELOUR)." Ann Oncol **22**(Supplement 5): O-0024.
Vaupel, P. and A. Mayer (2007). "Hypoxia in cancer: significance and impact on clinical outcome." Cancer Metastasis Rev **26**(2): 225-239.
Vleugel, M. M., A. E. Greijer, et al. (2005). "Differential prognostic impact of hypoxia induced and diffuse HIF-1alpha expression in invasive breast cancer." J Clin Pathol **58**(2): 172-177.
Wei, S. C., J. T. Liang, et al. (2009). "Preoperative serum placenta growth factor level is a prognostic biomarker in colorectal cancer." Dis Colon Rectum **52**(9): 1630-1636.
Wei, S. C., P. N. Tsao, et al. (2005). "Placenta growth factor expression is correlated with survival of patients with colorectal cancer." Gut **54**(5): 666-672.
Weickhardt, A., Williams, D. (2011). "Vascular endothelial growth factors (VEGF) and VEGF receptor expression as predictive biomarkers for benefit with bevacizumab in metastatic colorectal cancer (mCRC): Analysis of the phase III MAX study." J Clin Oncol **29**(suppl): abstr 3531.
Willett, C. G., D. G. Duda, et al. (2009). "Efficacy, safety, and biomarkers of neoadjuvant bevacizumab, radiation therapy, and fluorouracil in rectal cancer: a multidisciplinary phase II study." J Clin Oncol **27**(18): 3020-3026.
Wolpin, B. M. and R. J. Mayer (2008). "Systemic treatment of colorectal cancer." Gastroenterology **134**(5): 1296-1310.
Wouters, B. G. and J. M. Brown (1997). "Cells at intermediate oxygen levels can be more important than the "hypoxic fraction" in determining tumor response to fractionated radiotherapy." Radiat Res **147**(5): 541-550.

Appendix B: ECOG performance status scale

Grade	Performance scale
0	Able to carry out all normal activity without restriction
1	Restricted in physically strenuous activity but ambulatory and able to carry out light work.
2	Ambulatory and capable of all self-care but unable to carry out any work; up and about more than 50% of waking hours.
3	Capable of only limited self-care; confined to bed or chair more than 50% of waking hours
4	Completely disabled; cannot carry on any self-care; totally confined to bed or chair.

Appendix C: Common Terminology Criteria for Adverse Events

In the present study, adverse events and/or adverse drug reactions will be recorded according to the

Common Terminology Criteria for Adverse Events (CTCAE), version 4.03.

At the time this protocol was issued, the full CTC document was available on the NCI web site, at the following address: http://ctep.cancer.gov/reporting/ctc.html.

Another option is via the EORTC Headquarters web site www.eortc.be, which provides a link to the appropriate CTC web site. This link will be updated if the CTC address is changed.

Appendix D: RECIST v1.1

In this trial the 2009 updates version of the RECIST (v1.1) will be used. The version was published in European Journal of Cancer 2009

E.A. Eisenhauer, P Therasse
New response evaluation criteria in solid tumors: Revised RECIST guideline (version 1.1)
Europ. J Cancer 45 (2009) 228–247

Access to the guidelines is possible via the EORTC Headquarters web site www.eortc.be, which provides a link to the appropriate RECIST web site. This link will be updated if the RECIST address is changed.

Appendix E: TNM Clinical Classification

Colon and Rectum (ICD-O C18-C20)

According to *Colon and Rectum Cancer Staging 7th Edition (AJCC)*
Copyright © 2009 by American Joint Committee on Cancer (AJCC). All rights reserved.

T – Primary Tumor

TX. Primary tumor cannot be assessed
T0. No evidence of primary tumor
Tis. Carcinoma in situ: intraepithelial or invasion of lamina propria[1]
T1. Tumor invades submucosa
T2. Tumor invades muscularis propria
T3. Tumor invades through muscularis propria into pericolorectal tissues
T4a. Tumor penetrates to the surface of the visceral peritoneum[2]
T4b: Tumor directly invades or is adherent to other organs or structures [3]

N – Regional Lymph Nodes[4]

NX. Regional lymph nodes cannot be assessed
N0. No regional lymph node metastasis
N1. Metastasis in 1 to 3 regional lymph nodes
N1a. Metastasis in one regional lymph node
N1b. Metastasis in 2-3 regional lymph nodes
N1c. Tumor deposit(s) in the subserosa, mesentery, or nonperitonealized pericolic or perirectal tissues without regional nodal metastasis
N2. Metastasis in 4 or more regional lymph nodes
N2a. Metastasis in 4-6 regional lymph nodes
N2b. Metastasis in 7 or more regional lymph nodes

M – Distant Metastasis

M0. No distant metastasis
M1. Distant metastasis
M1a. Metastasis confined to one organ or site (for example, liver, lung, ovary, nonregional node)
M1b. Metastases in more than one organ/site or the peritoneum

Stage Grouping

Stage	T	N	M
0	Tis	N0	M0
I	T1	N0	M0
	T2	N0	M0
IIA	T3	N0	M0
IIB	T4a	N0	M0
IIC	T4b	N0	M0
IIIA	T1-T2	N1/N1c	M0
	T1	N2a	M0
IIIB	T3-T4a	N1/N1c	M0
	T2-T3	N2a	M0
	T1-T2	N2b	M0
IIIC	T4a	N2a	M0
	T3-T4a	N2b	M0
	T4b	N1-N2	M0
IVA	Any T	Any N	M1a
IVB	Any T	Any N	M1b

[1] Tis includes cancer cells confined within the glandular basement membrane (intraepithelial) or mucosal lamina propria (intramucosal) with no extension through the muscularis mucosae into the submucosa.
[2] Direct invasion in T4 includes invasion of other organs or other segments of the colorectum as a result of direct extension through the serosa, as confirmed on microscopic examination (for example, invasion of the sigmoid colon by a carcinoma of the cecum) or, for cancers in a retroperitoneal or subperitoneal location, direct invasion of other organs or structures by virtue of extension beyond the muscularis propria (that is, a tumor on the posterior wall of the descending colon invading the left kidney or lateral abdominal wall; or a mid or distal rectal cancer with invasion of prostate, seminal vesicles, cervix, or vagina).
[3] Tumor that is adherent to other organs or structures, grossly, is classified cT4b. However, if no tumor is present in the adhesion, microscopically, the classification should be pT1-4a depending on the anatomical depth of wall invasion. The V and L classifications should be used to identify the presence or absence of vascular or lymphatic invasion, whereas the PN site-specific factor should be used for perineural invasion.
[4] A satellite peritumoral nodule in the pericolorectal adipose tissue of a primary carcinoma without histologic evidence of residual lymph node in the nodule may represent discontinuous spread, venous invasion with extravascular spread (V1/2), or a totally replaced lymph node (N1/2). Replaced nodes should be counted separately as positive nodes in the N category, whereas discontinuous spread or venous invasion should be classified and counted in the Site-Specific Factor category Tumor Deposits (TD).

Appendix F: EORTC QLQ C30, CR29

GERMAN

EORTC QLQ-C30 (version 3.0)

Wir sind an einigen Angaben interessiert, die Sie und Ihre Gesundheit betreffen. Bitte beantworten Sie die folgenden Fragen selbst, indem Sie die Zahl ankreuzen, die am besten auf Sie zutrifft. Es gibt keine "richtigen" oder "falschen" Antworten. Ihre Angaben werden streng vertraulich behandelt.

Bitte tragen Sie Ihre Initialen ein: |_|_|_|_|

Ihr Geburtstag (Tag, Monat, Jahr): |_|_|_|_|_|_|_|_|

Das heutige Datum (Tag, Monat, Jahr): 31|_|_|_|_|_|_|_|_|

	Überhaupt nicht	Wenig	Mäßig	Sehr
1. Bereitet es Ihnen Schwierigkeiten sich körperlich anzustrengen (z.B. eine schwere Einkaufstasche oder einen Koffer zu tragen?)	1	2	3	4
2. Bereitet es Ihnen Schwierigkeiten, einen längeren Spaziergang zu machen?	1	2	3	4
3. Bereitet es Ihnen Schwierigkeiten, eine kurze Strecke außer Haus zu gehen?	1	2	3	4
4. Müssen Sie tagsüber im Bett liegen oder in einem Sessel sitzen?	1	2	3	4
5. Brauchen Sie Hilfe beim Essen, Anziehen, Waschen oder Benutzen der Toilette?	1	2	3	4

**Während der letzten Woche:**	Überhaupt nicht	Wenig	Mäßig	Sehr
6. Waren Sie bei Ihrer Arbeit oder bei anderen tagtäglichen Beschäftigungen eingeschränkt?	1	2	3	4
7. Waren Sie bei Ihren Hobbys oder anderen Freizeitbeschäftigungen eingeschränkt?	1	2	3	4
8. Waren Sie kurzatmig?	1	2	3	4
9. Hatten Sie Schmerzen?	1	2	3	4
10. Mussten Sie sich ausruhen?	1	2	3	4
11. Hatten Sie Schlafstörungen?	1	2	3	4
12. Fühlten Sie sich schwach?	1	2	3	4
13. Hatten Sie Appetitmangel?	1	2	3	4
14. War Ihnen übel?	1	2	3	4
15. Haben Sie erbrochen?	1	2	3	4

Bitte wenden

**Während der letzten Woche:**	**Überhaupt nicht**	**Wenig**	**Mäßig**	**Sehr**
16. Hatten Sie Verstopfung?	1	2	3	4
17. Hatten Sie Durchfall?	1	2	3	4
18. Waren Sie müde?	1	2	3	4
19. Fühlten Sie sich durch Schmerzen in Ihrem alltäglichen Leben beeinträchtigt?	1	2	3	4
20. Hatten Sie Schwierigkeiten sich auf etwas zu konzentrieren, z.B. auf das Zeitunglesen oder das Fernsehen?	1	2	3	4
21. Fühlten Sie sich angespannt?	1	2	3	4
22. Haben Sie sich Sorgen gemacht?	1	2	3	4
23. Waren Sie reizbar?	1	2	3	4
24. Fühlten Sie sich niedergeschlagen?	1	2	3	4
25. Hatten Sie Schwierigkeiten, sich an Dinge zu erinnern?	1	2	3	4
26. Hat Ihr körperlicher Zustand oder Ihre medizinische Behandlung Ihr Familienleben beeinträchtigt?	1	2	3	4
27. Hat Ihr körperlicher Zustand oder Ihre medizinische Behandlung Ihr Zusammensein oder Ihre gemeinsamen Unternehmungen mit anderen Menschen beeinträchtigt?	1	2	3	4
28. Hat Ihr körperlicher Zustand oder Ihre medizinische Behandlung für Sie finanzielle Schwierigkeiten mit sich gebracht?	1	2	3	4

Bitte kreuzen Sie bei den folgenden Fragen die Zahl zwischen 1 und 7 an, die am besten auf Sie zutrifft

29. Wie würden Sie insgesamt Ihren Gesundheitszustand während der letzten Woche einschätzen?

1	2	3	4	5	6	7
sehr schlecht						ausgezeichnet

30. Wie würden Sie insgesamt Ihre Lebensqualität während der letzten Woche einschätzen?

1	2	3	4	5	6	7
sehr schlecht						ausgezeichnet

© Copyright 1995 EORTC Study Group on Quality of Life. Alle Rechte vorbehalten. Version 3.0

EORTC QLQ – CR29

Patienten berichten manchmal die nachfolgend beschriebenen Symptome oder Probleme. Bitte beschreiben Sie, wie stark Sie diese Symptome oder Probleme während der letzten Woche empfunden haben. Kreuzen Sie bitte die Zahl an, die am ehesten auf Sie zutrifft.

	Während der letzten Woche:	Überhaupt nicht	Wenig	Mäßig	Sehr
31.	Mussten Sie tagsüber häufig Wasser lassen?	1	2	3	4
32.	Mussten Sie nachts häufig Wasser lassen?	1	2	3	4
33.	Kam es bei Ihnen zu unwillkürlichem Harnabgang?	1	2	3	4
34.	Hatten Sie Schmerzen beim Wasserlassen?	1	2	3	4
35.	Hatten Sie Bauchschmerzen?	1	2	3	4
36.	Hatten Sie Schmerzen im Gesäß/Analbereich/Rektum?	1	2	3	4
37.	Hatten Sie das Gefühl, dass Ihr Bauch gebläht ist?	1	2	3	4
38.	Hatten Sie Blut im Stuhl?	1	2	3	4
39.	Befand sich Schleim in Ihrem Stuhlgang?	1	2	3	4

	Während der letzten Woche:	Überhaupt nicht	Wenig	Mäßig	Sehr
40.	Hatten Sie einen trockenen Mund?	1	2	3	4
41.	Hatten Sie aufgrund Ihrer Behandlung Haarausfall?	1	2	3	4
42	Hatten Sie Probleme mit Ihrem Geschmackssinn?	1	2	3	4
43.	Waren Sie wegen Ihres zukünftigen Gesundheitszustandes besorgt?	1	2	3	4
44.	Haben Sie sich Sorgen über Ihr Gewicht gemacht?	1	2	3	4
45.	Fühlten Sie sich wegen Ihrer Erkrankung oder Behandlung körperlich weniger anziehend?	1	2	3	4
46.	Fühlten Sie sich wegen Ihrer Erkrankung oder Behandlung weniger weiblich/männlich?	1	2	3	4
47.	Waren Sie mit Ihrem Körper unzufrieden?	1	2	3	4
48.	Haben Sie ein Stoma (künstlicher Darmausgang)? (Zutreffendes bitte ankreuzen)	ja		nein	

Fortsetzung auf der nächsten Seite

Während der letzten Woche:

	Überhaupt nicht	Wenig	Mäßig	Sehr
**Beantworten Sie die folgenden Fragen bitte NUR, WENN SIE EINEN STOMABEUTEL TRAGEN. Fahren Sie ansonsten weiter unten fort:**				
49. Hatten Sie unfreiwillige Darmgasentweichungen/Flatulenzen aus Ihrem Stomabeutel?	1	2	3	4
50. Hatten Sie ungewollte Stuhlabgänge aus Ihrem Stomabeutel?	1	2	3	4
51. War die Haut um Ihr Stoma wund?	1	2	3	4
52. Gab es im Tagesverlauf häufige Beutelwechsel?	1	2	3	4
53. Fanden während der Nacht häufige Beutelwechsel statt?	1	2	3	4
54. War es Ihnen peinlich, ein Stoma zu haben?	1	2	3	4
55. Hatten Sie Probleme mit Ihrer Stomapflege?	1	2	3	4

**Beantworten Sie die folgenden Fragen NUR, WENN SIE KEINEN STOMABEUTEL TRAGEN:**				
49. Hatten Sie unfreiwillige Darmgasentweichungen/Flatulenzen aus Ihrem Darmausgang?	1	2	3	4
50. Hatten Sie ungewollte Stuhlabgänge aus Ihrem Darmausgang?	1	2	3	4
51. Hatten Sie wunde Haut in Ihrem Analbereich?	1	2	3	4
52. Gab es im Tagesverlauf häufige Stuhlgänge?	1	2	3	4
53. Gab es während der Nacht häufige Stuhlgänge?	1	2	3	4
54. Waren Ihnen die Stuhlgänge peinlich?	1	2	3	4

Während der letzten 4 Wochen:

	Überhaupt nicht	Wenig	Mäßig	Sehr
**Nur für Männer:**				
56. Wie sehr waren Sie an Sexualität interessiert?	1	2	3	4
57. Hatten Sie Schwierigkeiten, eine Erektion zu bekommen oder zu halten?	1	2	3	4

**Nur für Frauen:**				
58. Wie sehr waren Sie an Sexualität interessiert?	1	2	3	4
59. Verspürten Sie Schmerzen oder Beschwerden während des Geschlechtsverkehrs?	1	2	3	4

ENGLISH

EORTC QLQ-C30 (version 3)

We are interested in some things about you and your health. Please answer all of the questions yourself by circling the number that best applies to you. There are no "right" or "wrong" answers. The information that you provide will remain strictly confidential.

Please fill in your initials: |__|__|__|__|

Your birthdate (Day, Month, Year): |__|__|__|__|__|__|__|__|

Today's date (Day, Month, Year): 31 |__|__|__|__|__|__|__|__|

	Not at All	A Little	Quite a Bit	Very Much
1. Do you have any trouble doing strenuous activities, like carrying a heavy shopping bag or a suitcase?	1	2	3	4
2. Do you have any trouble taking a long walk?	1	2	3	4
3. Do you have any trouble taking a short walk outside of the house?	1	2	3	4
4. Do you need to stay in bed or a chair during the day?	1	2	3	4
5. Do you need help with eating, dressing, washing yourself or using the toilet?	1	2	3	4

**During the past week:**	Not at All	A Little	Quite a Bit	Very Much
6. Were you limited in doing either your work or other daily activities?	1	2	3	4
7. Were you limited in pursuing your hobbies or other leisure time activities?	1	2	3	4
8. Were you short of breath?	1	2	3	4
9. Have you had pain?	1	2	3	4
10. Did you need to rest?	1	2	3	4
11. Have you had trouble sleeping?	1	2	3	4
12. Have you felt weak?	1	2	3	4
13. Have you lacked appetite?	1	2	3	4
14. Have you felt nauseated?	1	2	3	4
15. Have you vomited?	1	2	3	4
16. Have you been constipated?	1	2	3	4

Please go on to the next page

ENGLISH

During the past week:	Not at All	A Little	Quite a Bit	Very Much
17. Have you had diarrhea?	1	2	3	4
18. Were you tired?	1	2	3	4
19. Did pain interfere with your daily activities?	1	2	3	4
20. Have you had difficulty in concentrating on things, like reading a newspaper or watching television?	1	2	3	4
21. Did you feel tense?	1	2	3	4
22. Did you worry?	1	2	3	4
23. Did you feel irritable?	1	2	3	4
24. Did you feel depressed?	1	2	3	4
25. Have you had difficulty remembering things?	1	2	3	4
26. Has your physical condition or medical treatment interfered with your family life?	1	2	3	4
27. Has your physical condition or medical treatment interfered with your social activities?	1	2	3	4
28. Has your physical condition or medical treatment caused you financial difficulties?	1	2	3	4

For the following questions please circle the number between 1 and 7 that best applies to you

29. How would you rate your overall health during the past week?

1	2	3	4	5	6	7
Very poor						Excellent

30. How would you rate your overall quality of life during the past week?

1	2	3	4	5	6	7
Very poor						Excellent

© Copyright 1995 EORTC Quality of Life Group. All rights reserved. Version 3.0

ENGLISH

EORTC QLQ – CR29

Patients sometimes report that they have the following symptoms or problems. Please indicate the extent to which you have experienced these symptoms or problems during the past week. Please answer by circling the number that best applies to you.

During the past week:	Not at All	A Little	Quite a Bit	Very Much
31. Did you urinate frequently during the day?	1	2	3	4
32. Did you urinate frequently during the night?	1	2	3	4
33. Have you had any unintentional release (leakage) of urine?	1	2	3	4
34. Did you have pain when you urinated?	1	2	3	4
35. Did you have abdominal pain?	1	2	3	4
36. Did you have pain in your buttocks/anal area/rectum?	1	2	3	4
37. Did you have a bloated feeling in your abdomen?	1	2	3	4
38. Have you had blood in your stools?	1	2	3	4
39. Have you had mucus in your stools?	1	2	3	4
40. Did you have a dry mouth?	1	2	3	4
41. Have you lost hair as a result of your treatment?	1	2	3	4
42. Have you had problems with your sense of taste?	1	2	3	4

During the past week:	Not at All	A Little	Quite a Bit	Very Much
43. Were you worried about your health in the future?	1	2	3	4
44. Have you worried about your weight?	1	2	3	4
45. Have you felt physically less attractive as a result of your disease or treatment?	1	2	3	4
46. Have you been feeling less feminine/masculine as a result of your disease or treatment?	1	2	3	4
47. Have you been dissatisfied with your body?	1	2	3	4

48. Do you have a stoma bag (colostomy/ileostomy)?
(please circle the correct answer) **Yes** **No**

Please go on to the next page

ENGLISH

During the past week:

	Not at All	A Little	Quite a Bit	Very Much
**Answer these questions ONLY IF YOU HAVE A STOMA BAG, if not please continue below:**				
49. Have you had unintentional release of gas/flatulence from your stoma bag?	1	2	3	4
50. Have you had leakage of stools from your stoma bag?	1	2	3	4
51. Have you had sore skin around your stoma?	1	2	3	4
52. Did frequent bag changes occur during the day?	1	2	3	4
53. Did frequent bag changes occur during the night?	1	2	3	4
54. Did you feel embarrassed because of your stoma?	1	2	3	4
55. Did you have problems caring for your stoma?	1	2	3	4

**Answer these questions ONLY IF YOU DO NOT HAVE A STOMA BAG:**				
49. Have you had unintentional release of gas/flatulence from your back passage?	1	2	3	4
50. Have you had leakage of stools from your back passage?	1	2	3	4
51. Have you had sore skin around your anal area?	1	2	3	4
52. Did frequent bowel movements occur during the day?	1	2	3	4
53. Did frequent bowel movements occur during the night?	1	2	3	4
54. Did you feel embarrassed because of your bowel movement?	1	2	3	4

During the past 4 weeks:

	Not at All	A Little	Quite a Bit	Very Much
**For men only:**				
56. To what extent were you interested in sex?	1	2	3	4
57. Did you have difficulty getting or maintaining an erection?	1	2	3	4

**For women only:**				
58. To what extent were you interested in sex?	1	2	3	4
59. Did you have pain or discomfort during intercourse?	1	2	3	4

© QLQ-CR29 Copyright 2006 EORTC Quality of Life Group. All rights reserved. Version 2.1
